# Supplementary material for: An Effective and Promising Strategy for Plant Protection: Synthesis of L-Carvone-Based Thiazolinone–Hydrazone/Nanochitosan Complexes with Antifungal Activity and Sustained Releasing Performance
Source: Int J Mol Sci. 2024 Apr 23;25(9):4595. doi: 10.3390/ijms25094595 (PMC11083649; doi:10.3390/ijms25094595)
Supplement: Supplementary file 1 [file ijms-25-04595-s001.zip › ijms-2955616-supplementary.pdf]

Supplementary Material for

An Effective and Promising Strategy for Plant Protection: Synthesis of *L*-Carvone-Based Thiazolinone–Hydrazone/ Nanochitosan Complexes with Antifungal Activity and Sustained Releasing Performance

Baoyu Li [1,2], Wengui Duan [1,2,*], Guishan Lin [1,2,*], Xianli Ma [1,2], Rongzhu Wen [1,2] and Zhaolei Zhang [1,2]

1 School of Chemistry and Chemical Engineering, Guangxi University, Nanning 530004, China; baoyuli1111@163.com (B.L.); maxl0919@gxu.edu.cn (X.M.); rongzhuwen@163.com (R.W.); 2114301097@st.gxu.edu.cn (Z.Z.)

2 Guangxi Colleges and Universities Key Laboratory of Applied Chemistry Technology and Resource Development, Nanning 530004, China

* Correspondence: wgduan@gxu.edu.cn (W.D.); gslin@gxu.edu.cn (G.L.)

Table S1. Antifungal activity of compounds **4a** ~ **4y** against eight phytopathogenic fungi.

Compounds	Relative Inhibition Rates/%							
	FO	CA	PP	AS	GZ	RS	BM	CO
**4a** (Ar = Ph)	26.4	68.5	17.1	63.7	31.7	25.3	17.8	36.5
**4b** (Ar = 2-CH_3 Ph)	30.0	66.1	17.1	63.7	31.7	25.3	19.8	36.5
**4c** (Ar = 3-CH_3 Ph)	24.5	61.2	10.0	58.8	20.9	22.9	25.7	34.5
**4d** (Ar = 4-CH_3 Ph)	22.7	61.2	10.0	53.9	29.6	25.3	19.8	32.4
**4e** (Ar = 2-Cl Ph)	24.5	61.2	52.9	51.5	10.0	26.5	15.9	28.4
**4f** (Ar = 3-Cl Ph)	19.1	66.1	35.0	61.2	18.7	24.1	17.8	28.4
**4g** (Ar = 4-Cl Ph)	28.2	71.0	35.0	66.1	38.3	22.9	23.7	40.6
**4h** (Ar = 2-F Ph)	62.7	61.2	88.6	66.1	53.5	22.9	19.8	61.0
**4i** (Ar = 3-F Ph)	19.1	58.8	38.6	49.0	10.0	26.5	15.9	28.4
**4j** (Ar = 4-F Ph)	44.5	71.0	70.7	53.9	31.7	20.6	57.1	61.0
**4k** (Ar = 2-OCH_3 Ph)	26.4	66.1	35.0	66.1	20.9	21.8	25.7	36.5
**4l** (Ar = 3-OCH_3 Ph)	35.5	71.0	35.0	68.5	27.4	20.6	27.6	44.7
**4m** (Ar = 4-OCH_3 Ph)	19.1	58.8	60.0	61.2	36.1	22.9	15.9	30.4
**4n** (Ar = 2-NO_2 Ph)	24.5	68.5	52.9	58.8	10.0	20.6	19.8	28.4
**4o** (Ar = 4-NO_2 Ph)	30.0	66.1	70.7	68.5	27.4	22.9	33.5	40.6
**4p** (Ar = 3-CN Ph)	28.2	71.0	60.0	63.7	40.4	21.8	19.8	28.4
**4q** (Ar = 3-Br Ph)	30.0	68.5	10.0	66.1	36.1	22.9	21.8	32.4
**4r** (Ar = 3-OH Ph)	22.7	58.8	52.9	53.9	10.0	22.9	15.9	30.4
**4s** (Ar = 4-OCF_3 Ph)	26.4	68.5	52.9	56.3	20.9	20.6	15.9	32.4
**4t** (Ar = 3-CF_3 Ph)	24.5	61.2	70.7	61.2	27.4	22.9	19.8	36.5
**4u** (Ar = 4-$N(CH_3)_2$ Ph)	28.2	71.0	60.0	68.5	27.4	20.6	15.9	36.5
**4v** (Ar = 2, 4-OCH_3 Ph)	28.2	66.1	52.9	61.2	31.7	26.5	19.8	34.5
**4w** (Ar = 2-OH-3-OCH_3 Ph)	17.3	63.7	70.7	66.1	40.4	20.6	27.6	32.4
**4x** (Ar = α-thienyl)	22.7	66.1	70.7	58.8	31.7	25.3	19.8	28.4
**4y** (Ar = α-pyridyl)	19.1	71.0	52.9	66.1	33.9	22.9	15.9	30.4
chlorothalonil	87.2	63.0	95.8	35.5	67.9	81.2	85.0	100

FO = *Fusarium Oxysporum f. sp. Cucumerinum*; CA = *Cercospora Arachidicola*; PP = *Physalospora Piricola*; AS = *Alternaria Solani*; GZ = *Gibberella Zeae*; RS = *Rhzioeotnia Solani*; BM = *Bipolaris Maydis*; CO = *Colleterichum Orbicalare.*

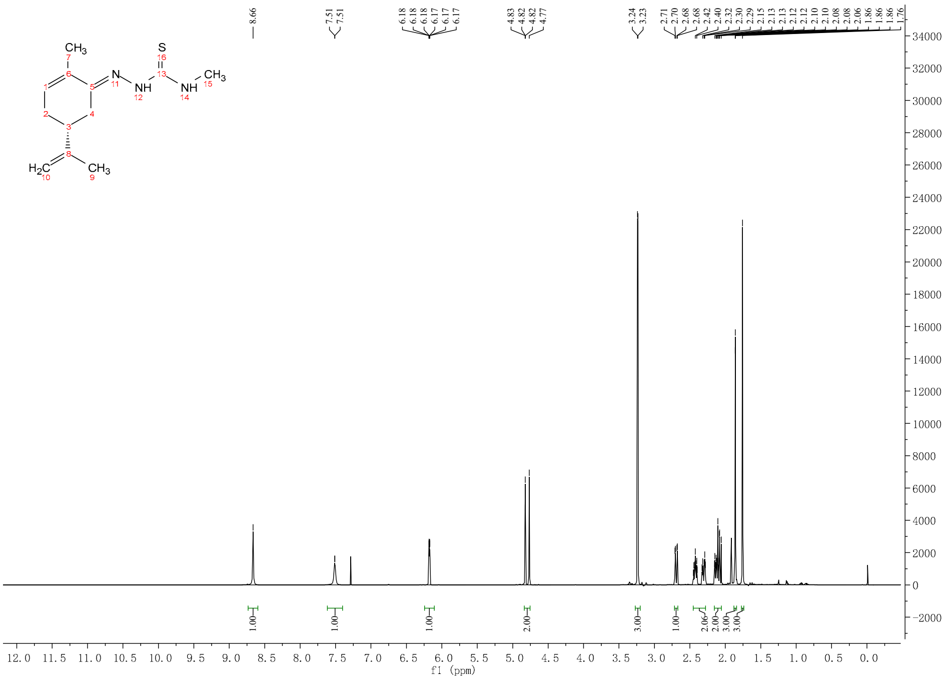

Figure S1 ^{1}H NMR spectrum of intermediate **2**.

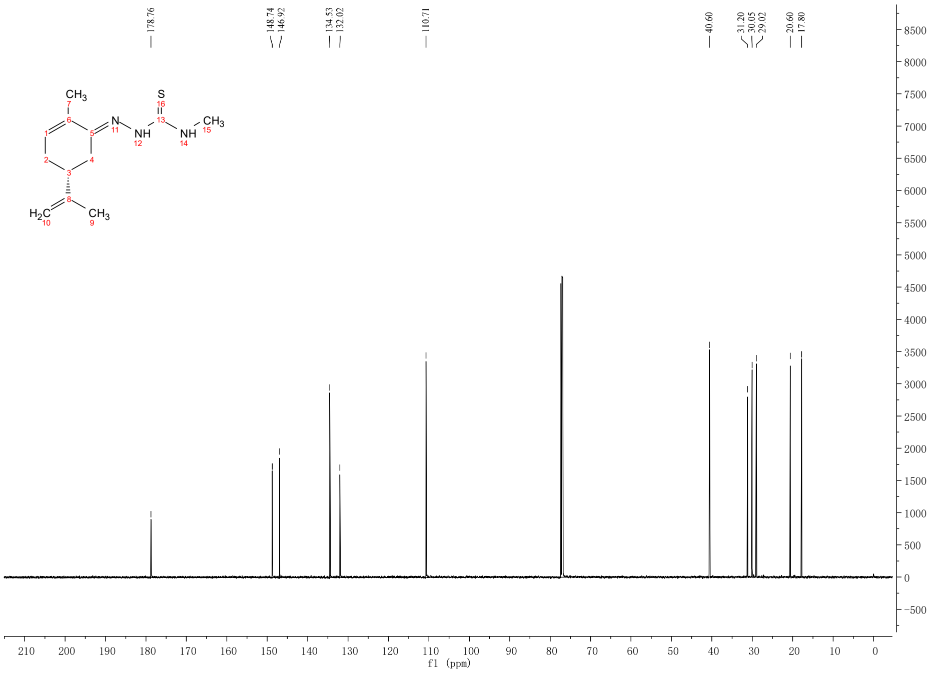

Figure S2 ^{13}C NMR spectrum of intermediate **2**.

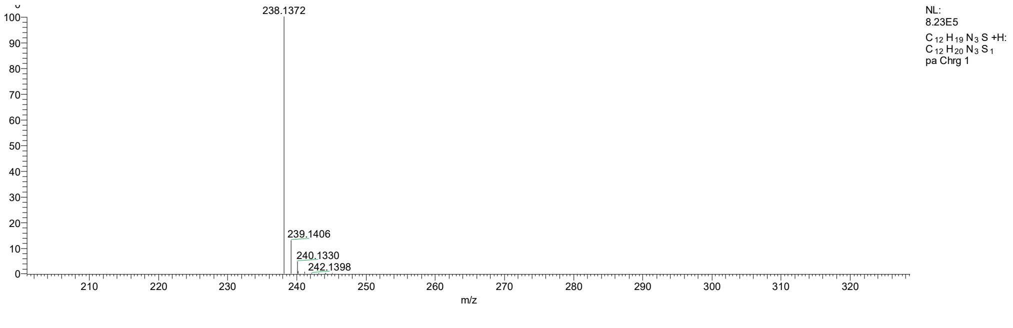

Figure S3 HRMS spectrum of intermediate **2**.

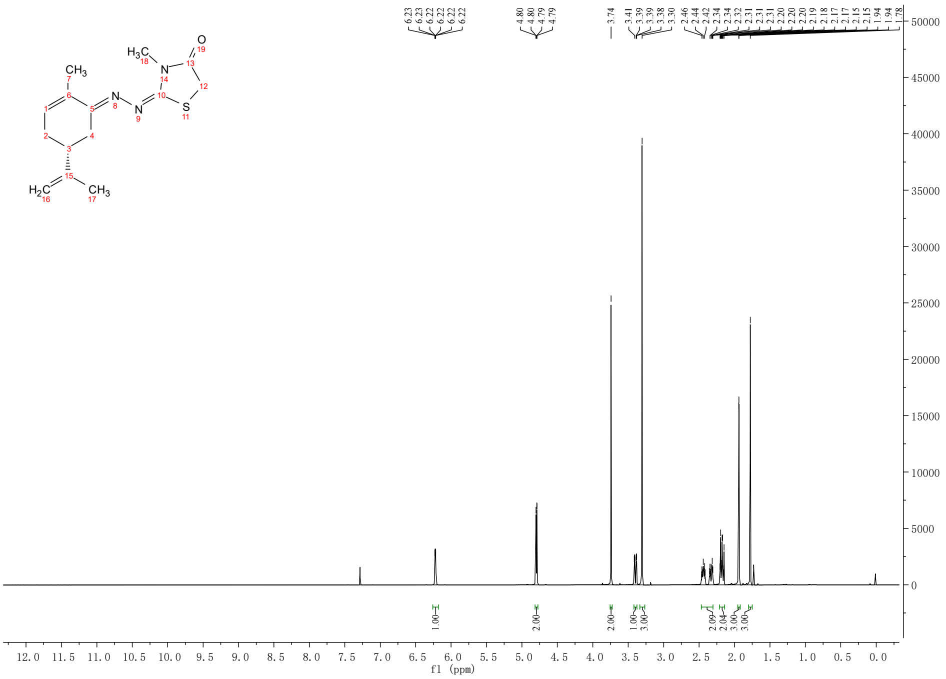

Figure S4 ^{1}H NMR spectrum of intermediate **3**.

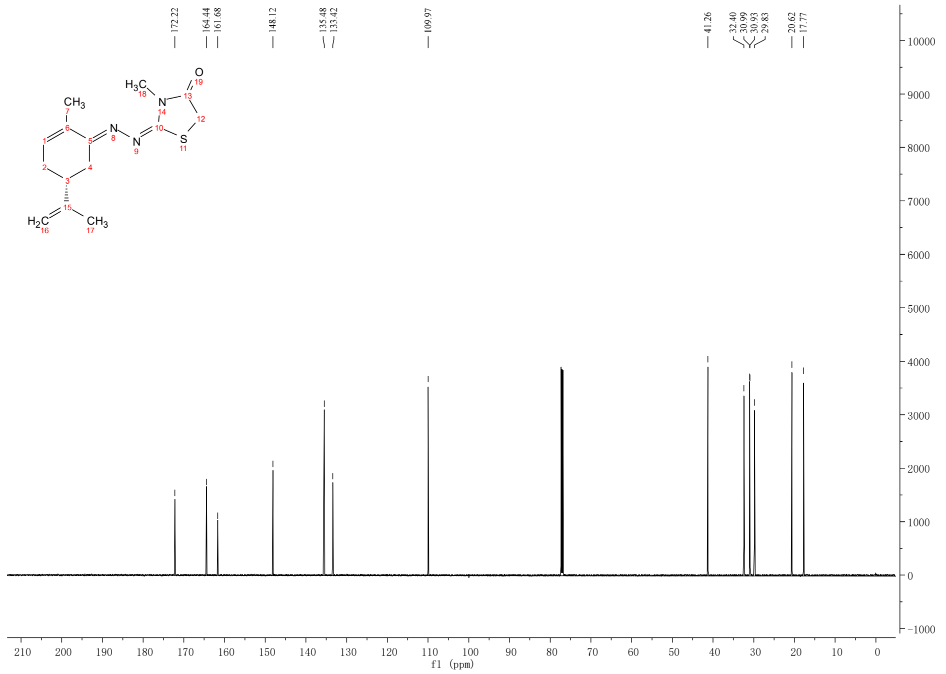

Figure S5 ^{13}C NMR spectrum of intermediate **3**.

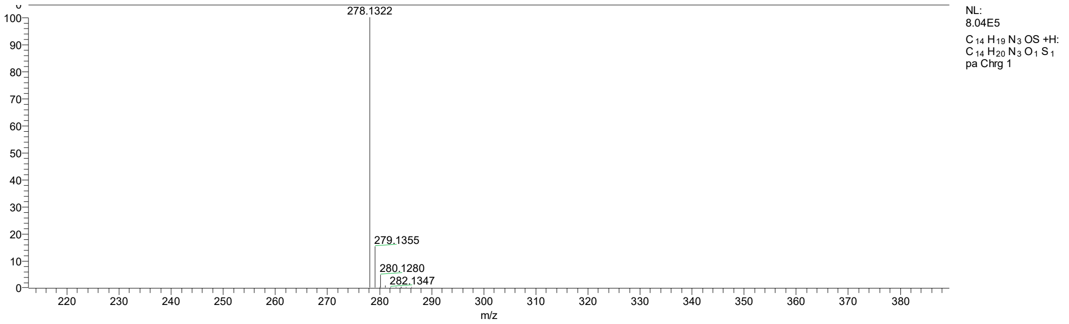

Figure S6 HRMS spectrum of intermediate **3**.

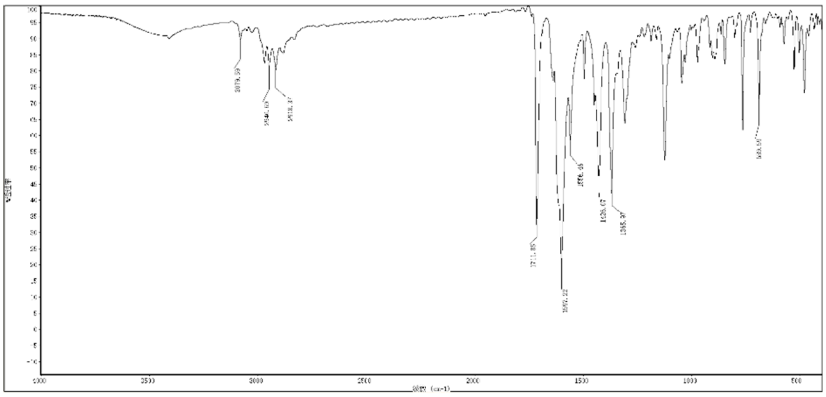

Figure S7 FT-IR spectrum of compound **4a**.

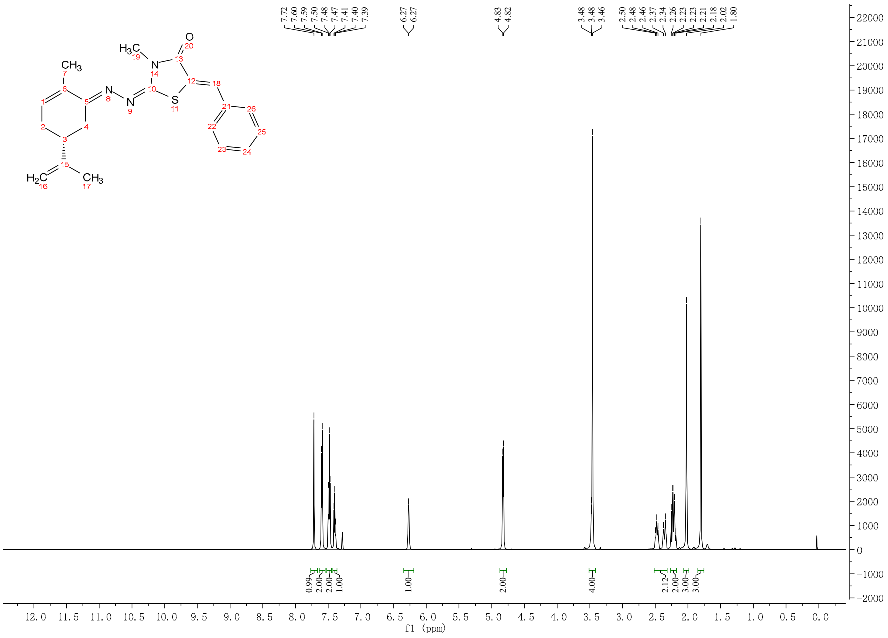

Figure S8 ^{1}H NMR spectrum of compound **4a**.

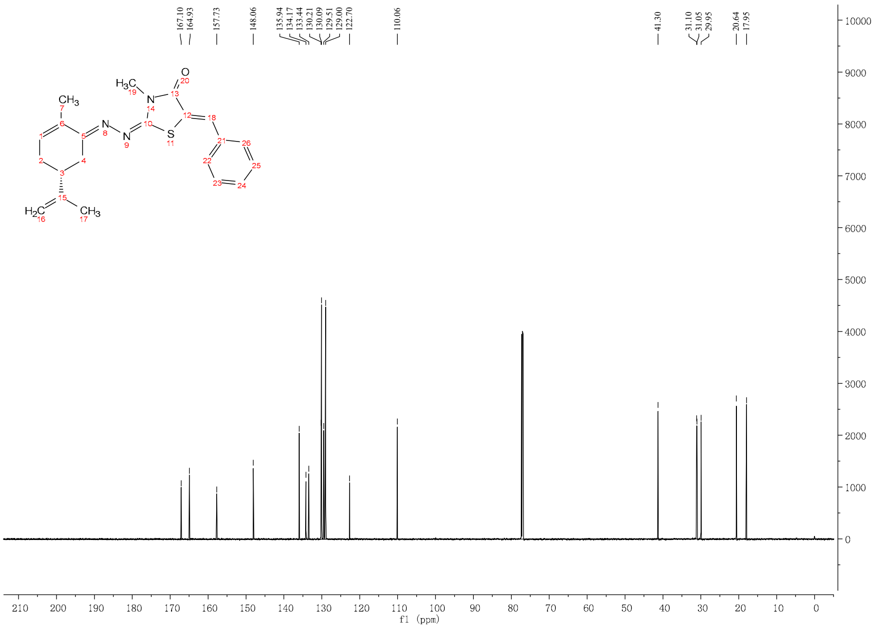

Figure S9 ^{13}C NMR spectrum of compound **4a**.

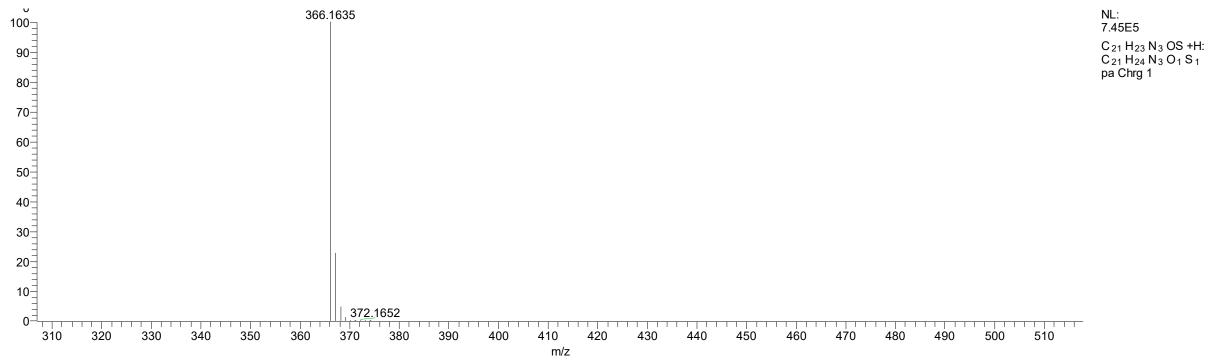

Figure S10 HRMS spectrum of compound **4a**.

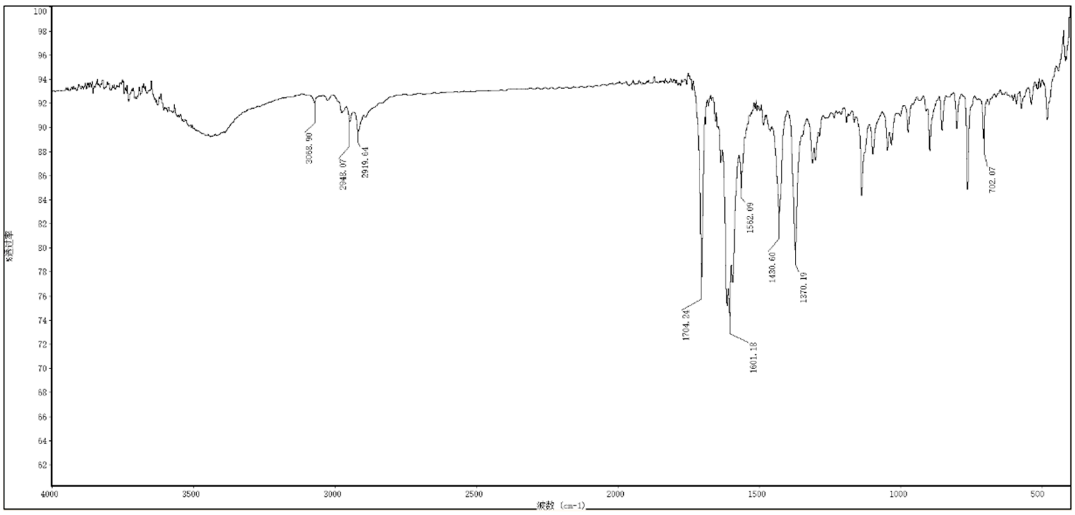

Figure S11 IR spectrum of compound **4b**.

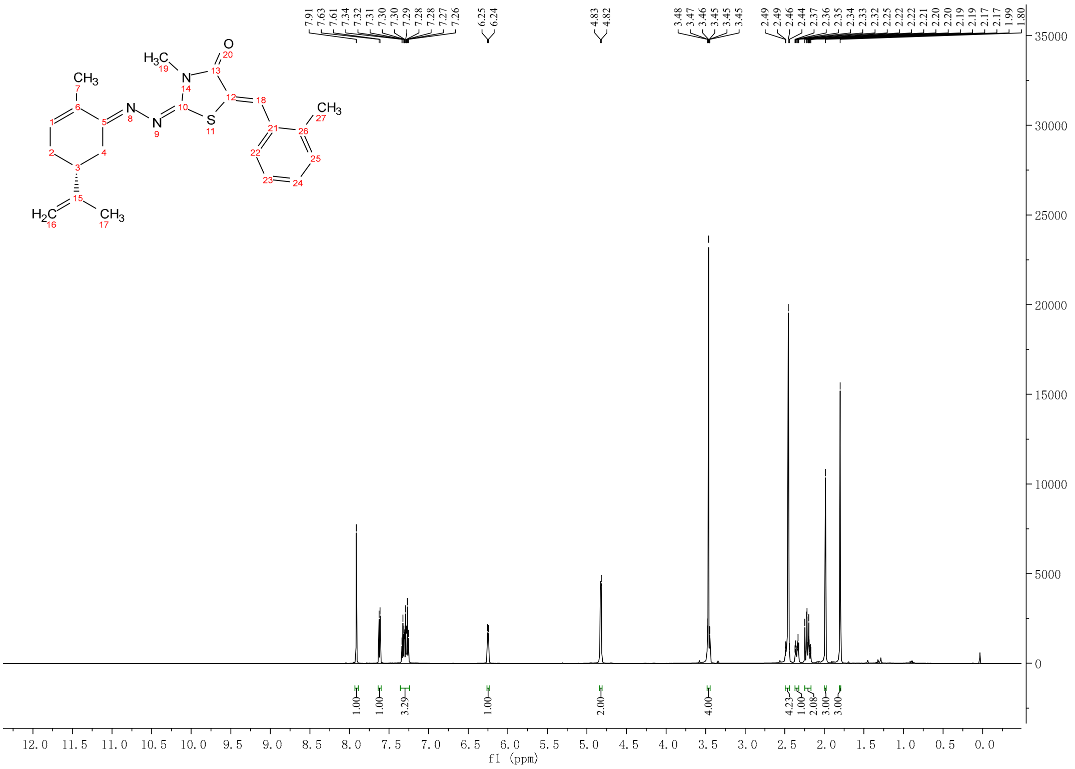

Figure S12 ^{1}H NMR spectrum of compound **4b**.

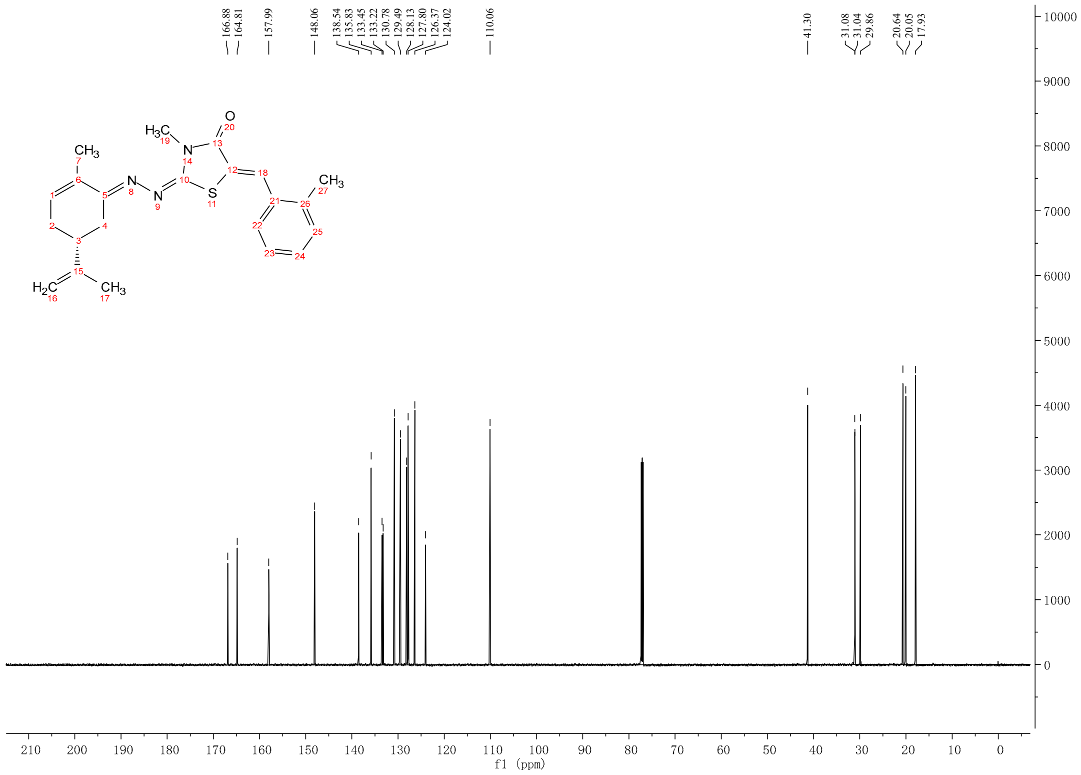

Figure S13 ^{13}C NMR spectrum of compound **4b**.

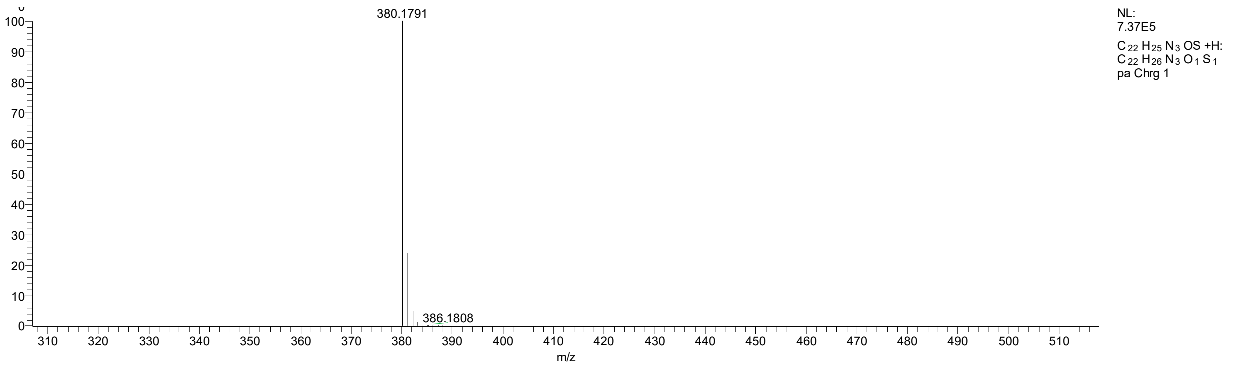

Figure S14 HRMS spectrum of compound **4b**.

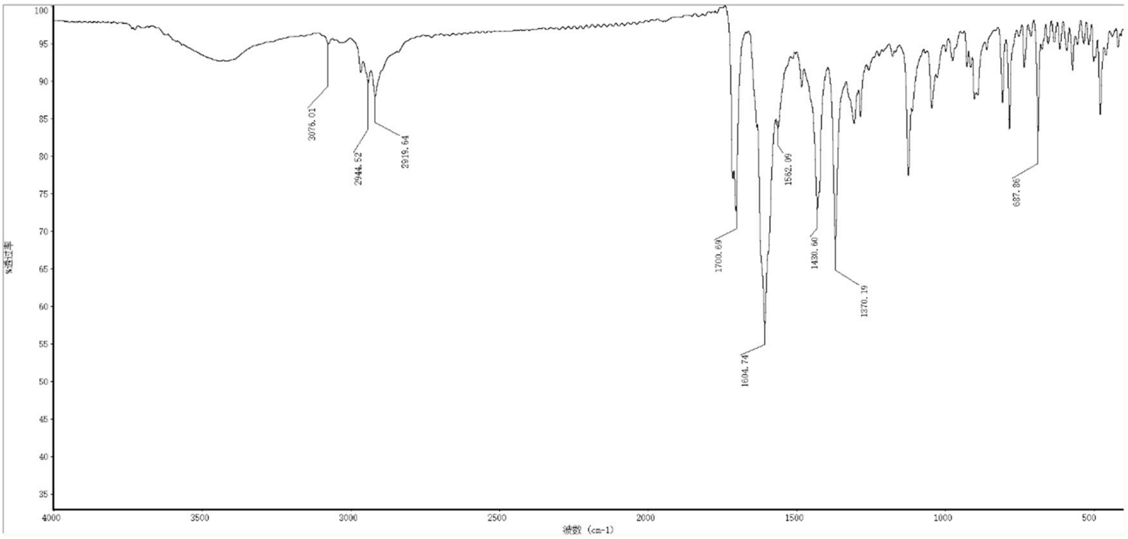

Figure S15 IR spectrum of compound **4c**.

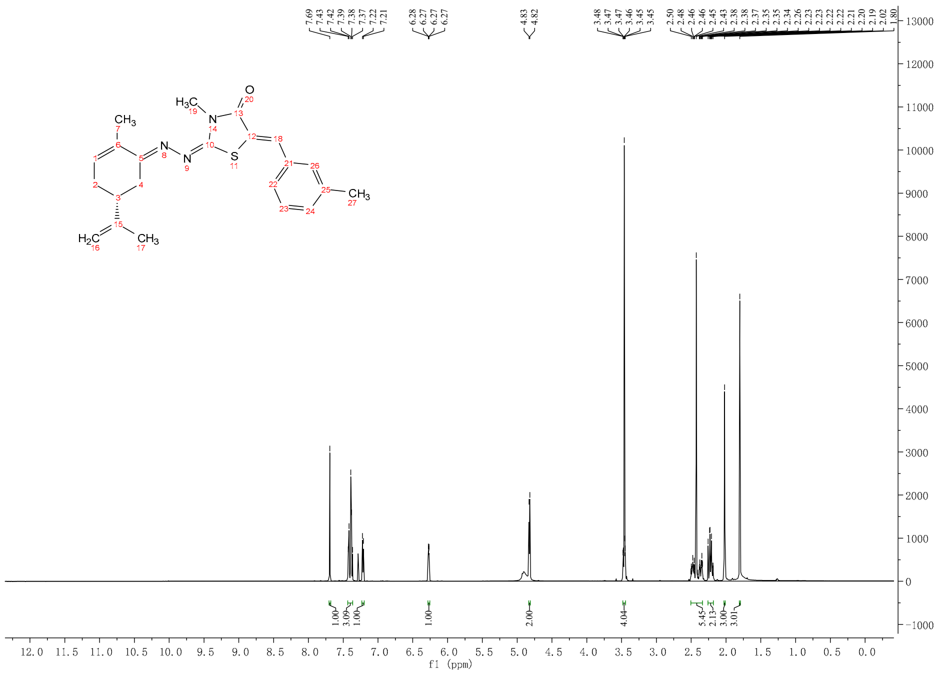

Figure S16 ^{1}H NMR spectrum of compound **4c**.

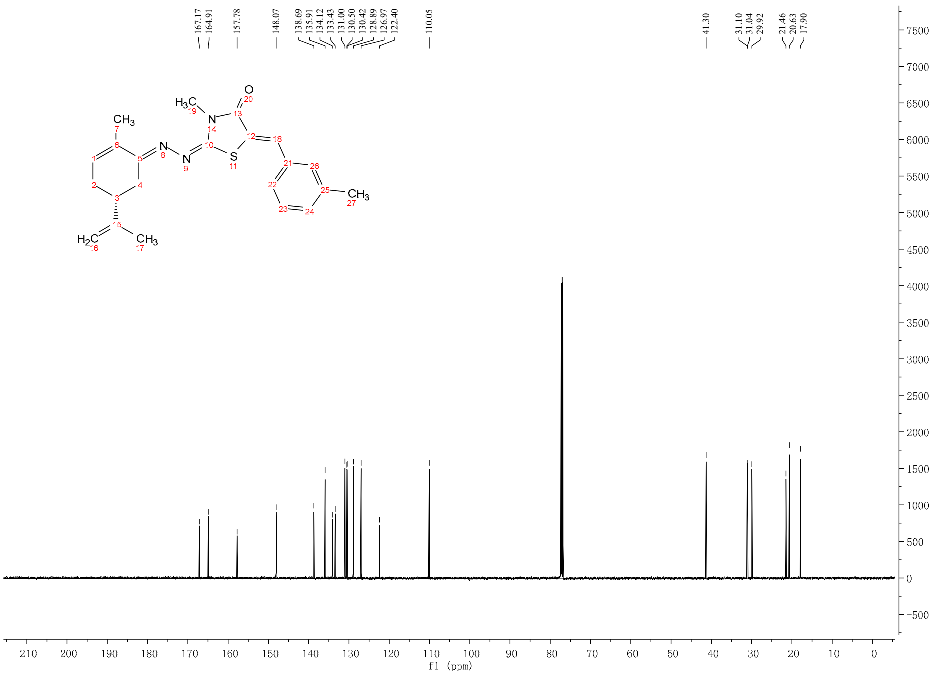

Figure S17 ^{13}C NMR spectrum of compound **4c**.

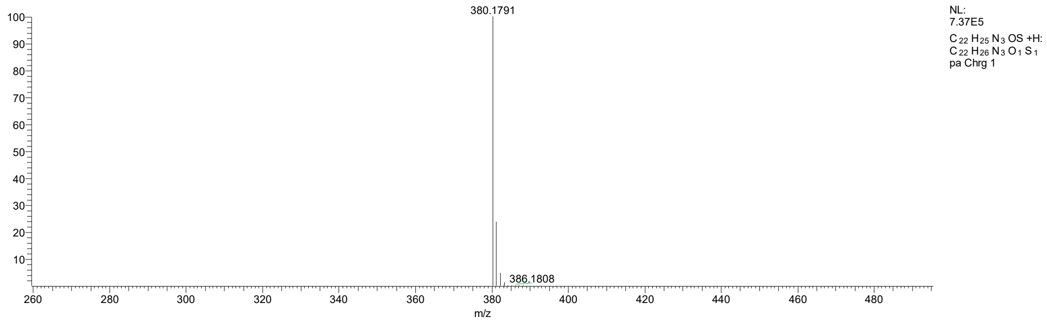

Figure S18 HRMS spectrum of compound **4c**.

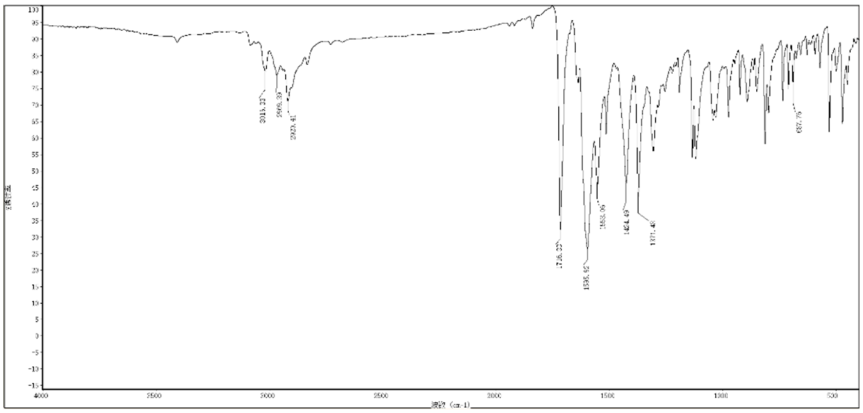

Figure S19 IR spectrum of compound **4d**.

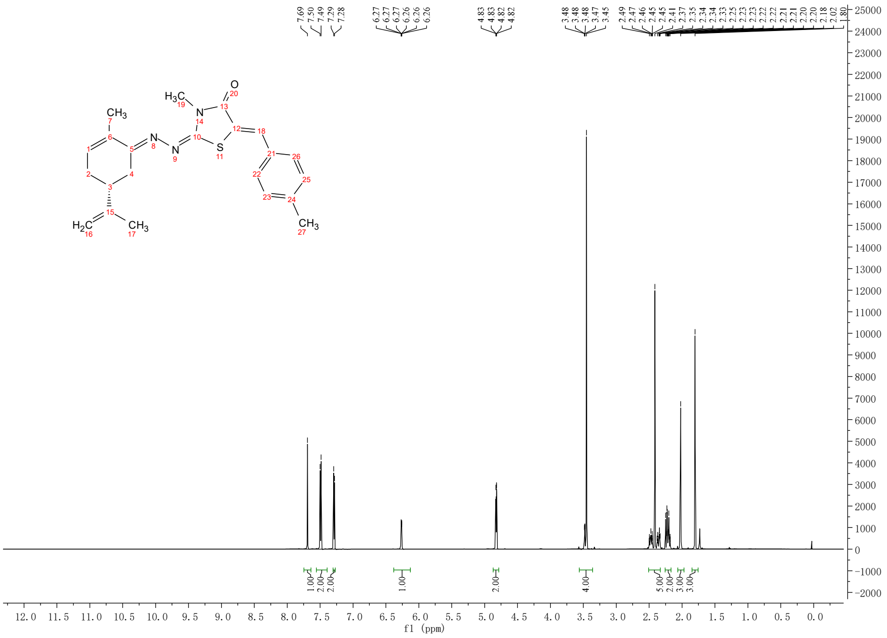

Figure S20 ^{1}H NMR spectrum of compound **4d**.

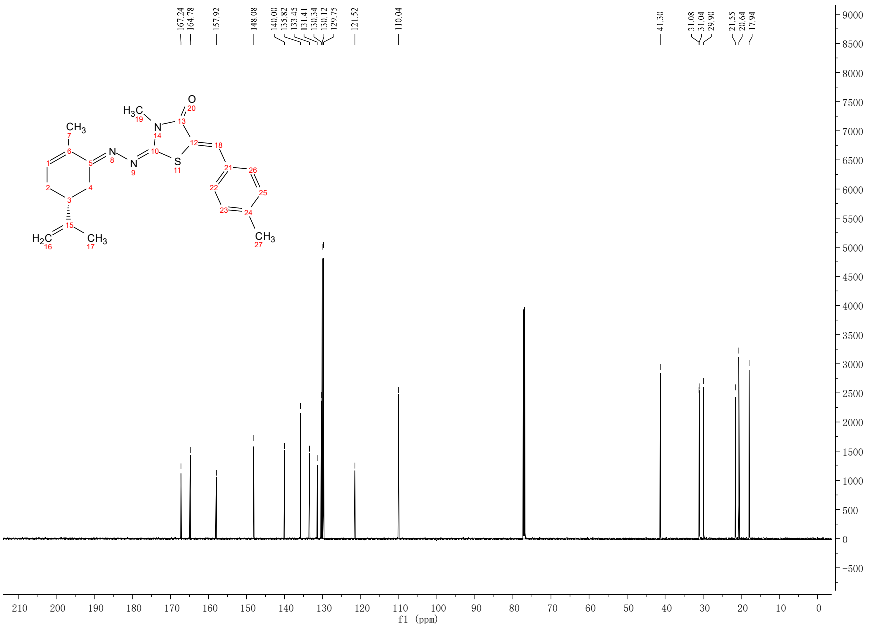

Figure S21 ^{13}C NMR spectrum of compound **4d**.

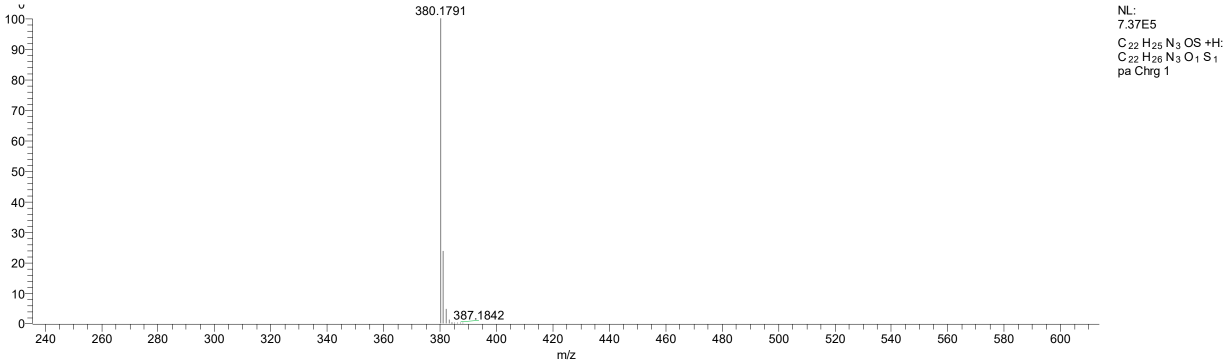

Figure S22 HRMS spectrum of compound **4d**.

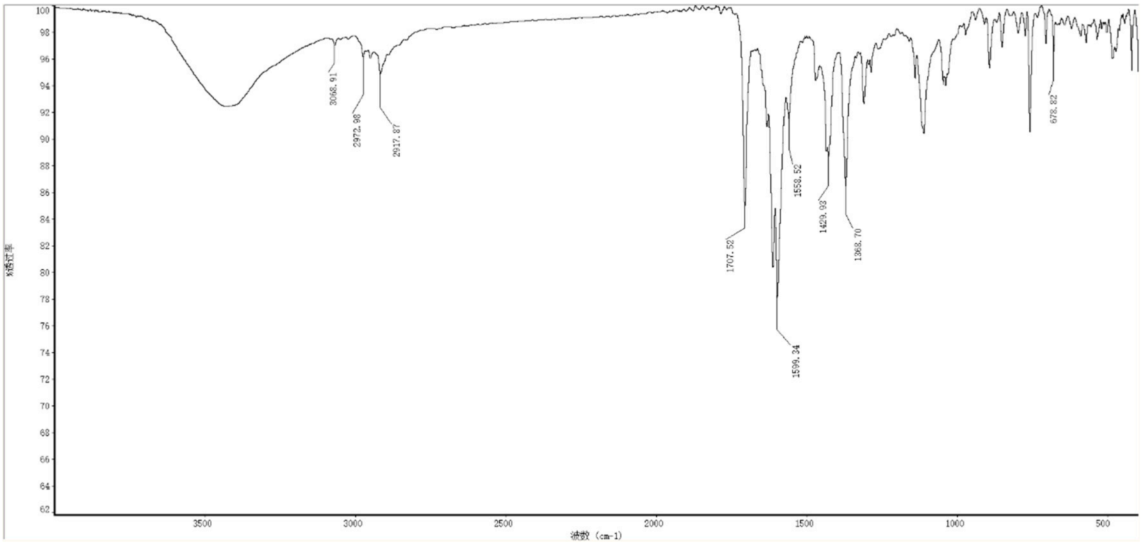

Figure S23 IR spectrum of compound **4e**.

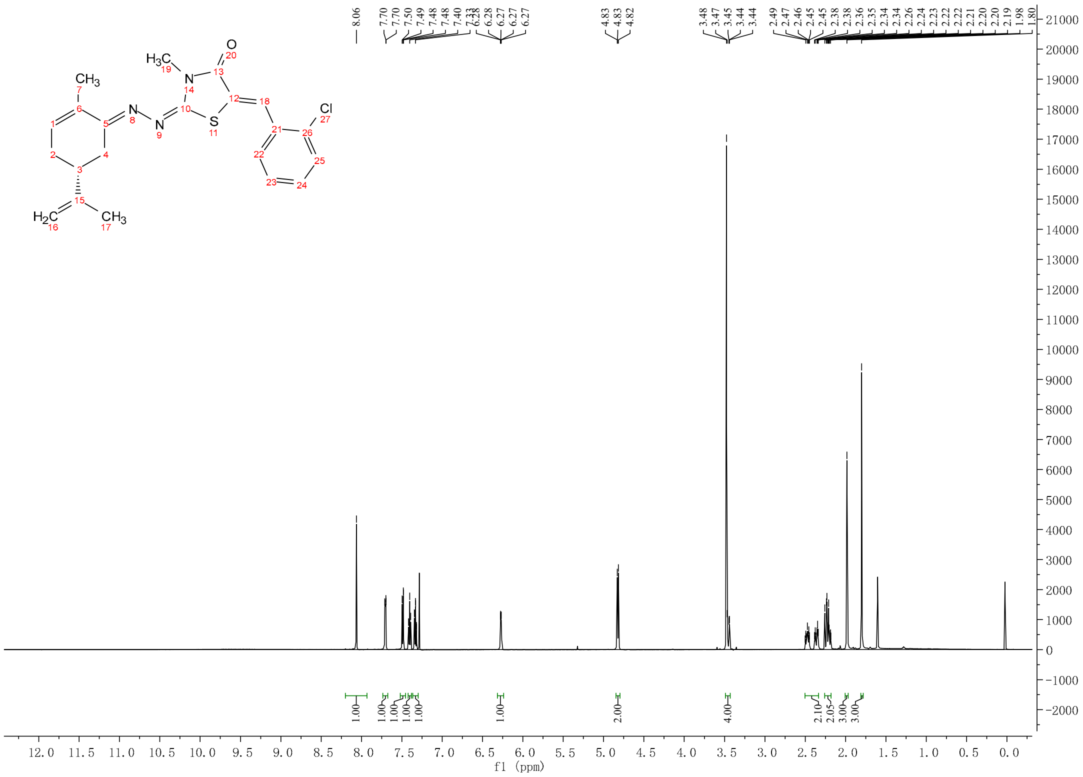

Figure S24 ^{1}H NMR spectrum of compound **4e**.

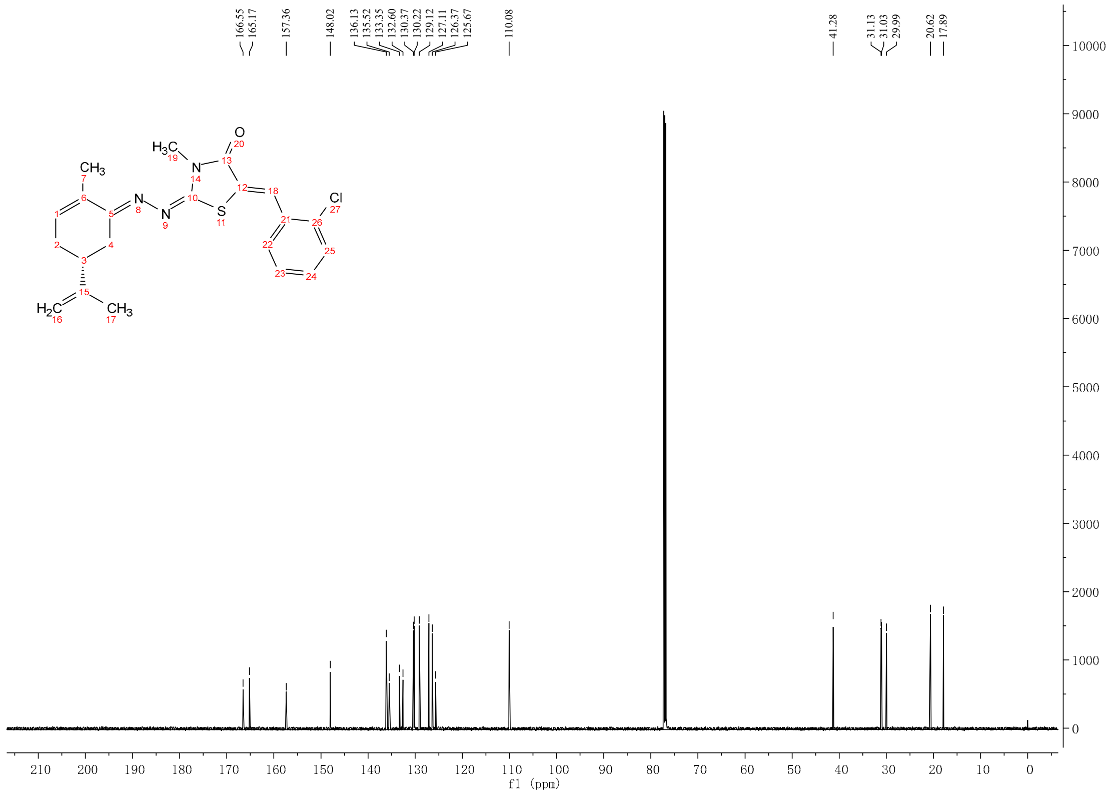

Figure S25 ^{13}C NMR spectrum of compound **4e**.

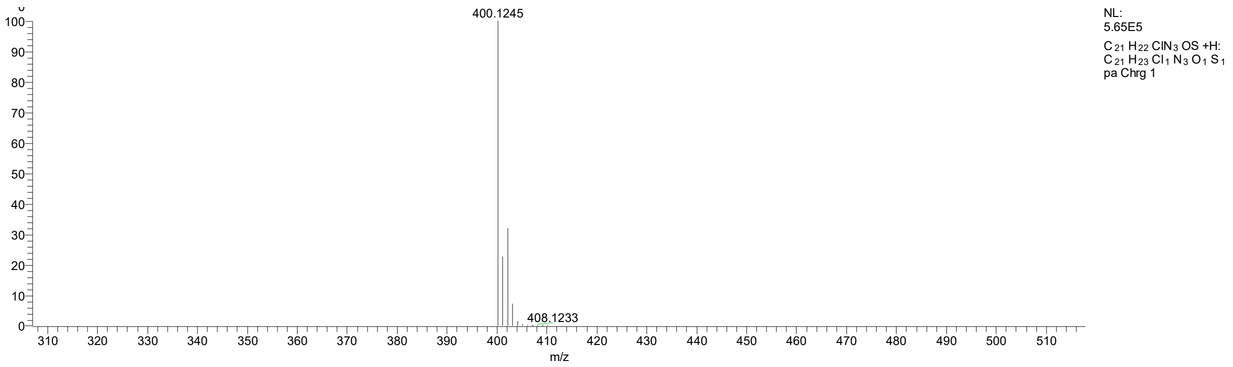

Figure S26 HRMS spectrum of compound **4e**.

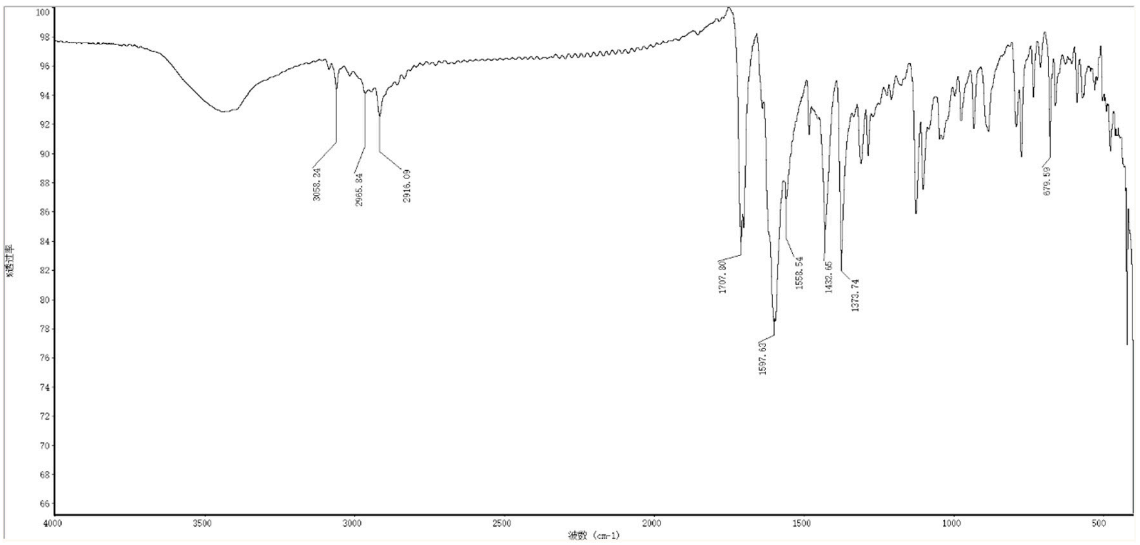

Figure S27 IR spectrum of compound **4f**.

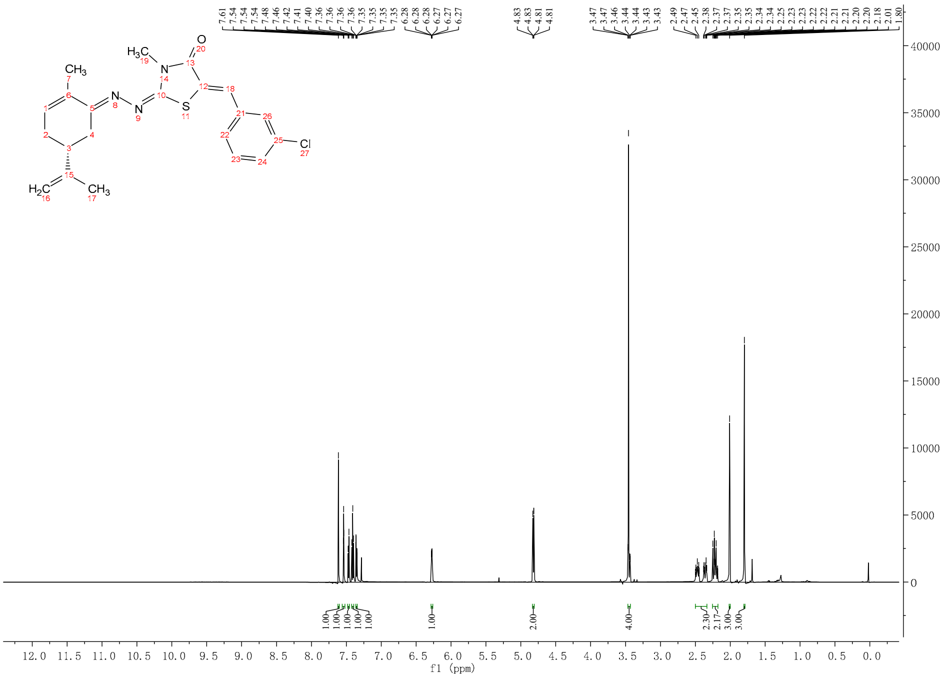

Figure S28 ^{1}H NMR spectrum of compound **4f**.

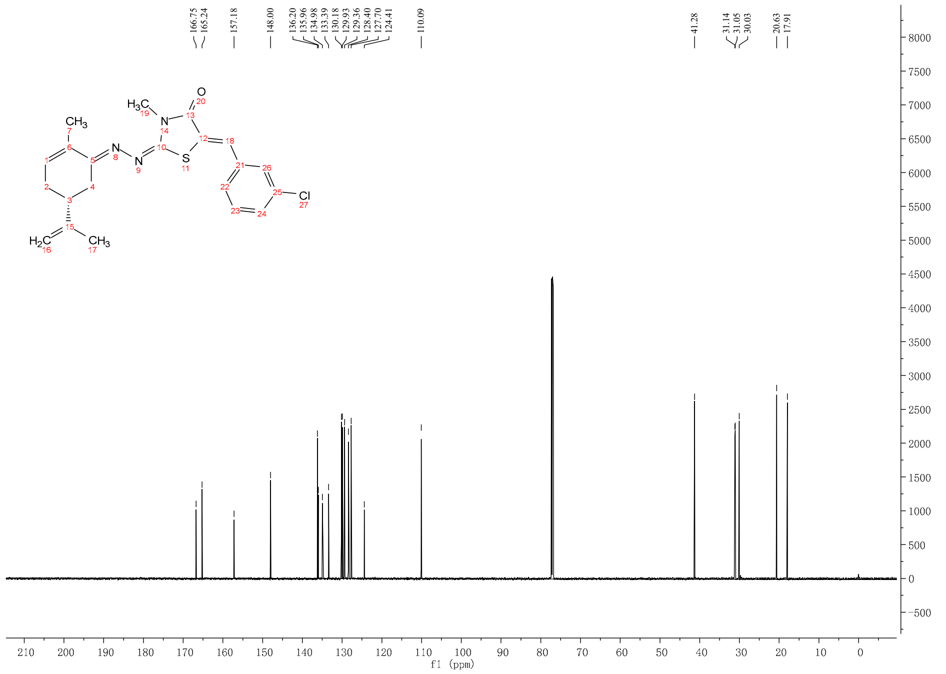

Figure S29 ^{13}C NMR spectrum of compound **4f**.

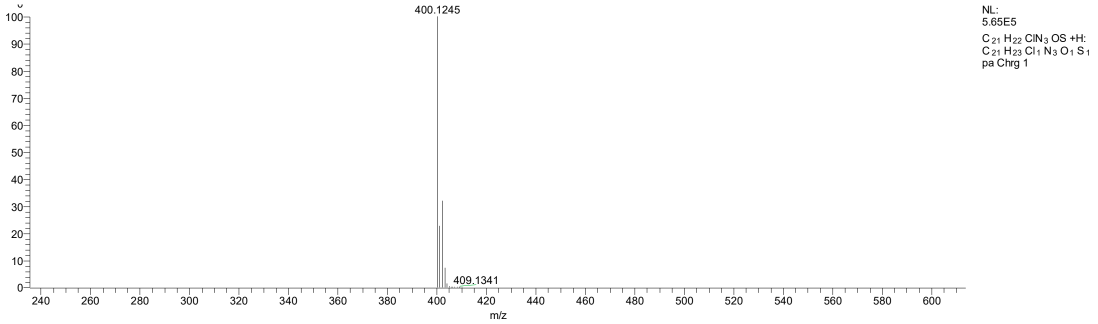

Figure S30 HRMS spectrum of compound **4f**.

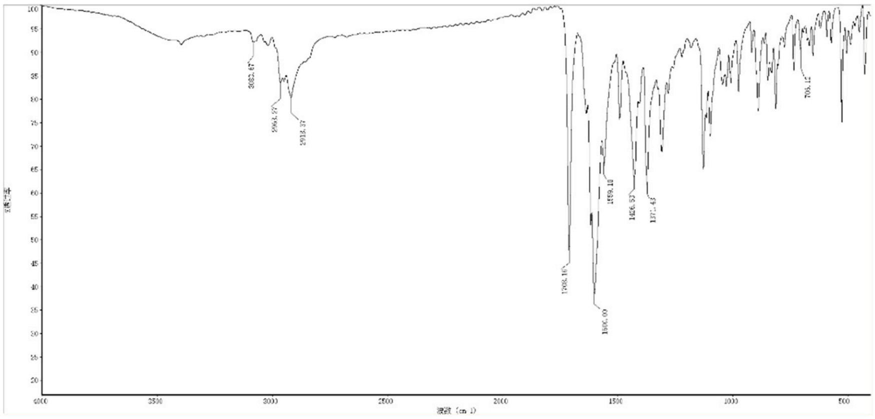

Figure S31 IR spectrum of compound **4g**.

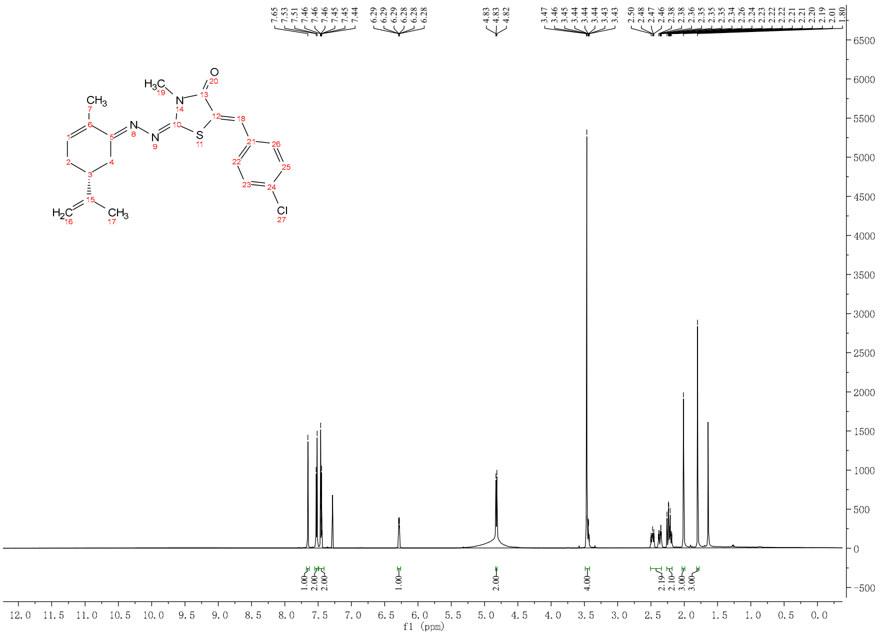

Figure S32 ^{1}H NMR spectrum of compound **4g**.

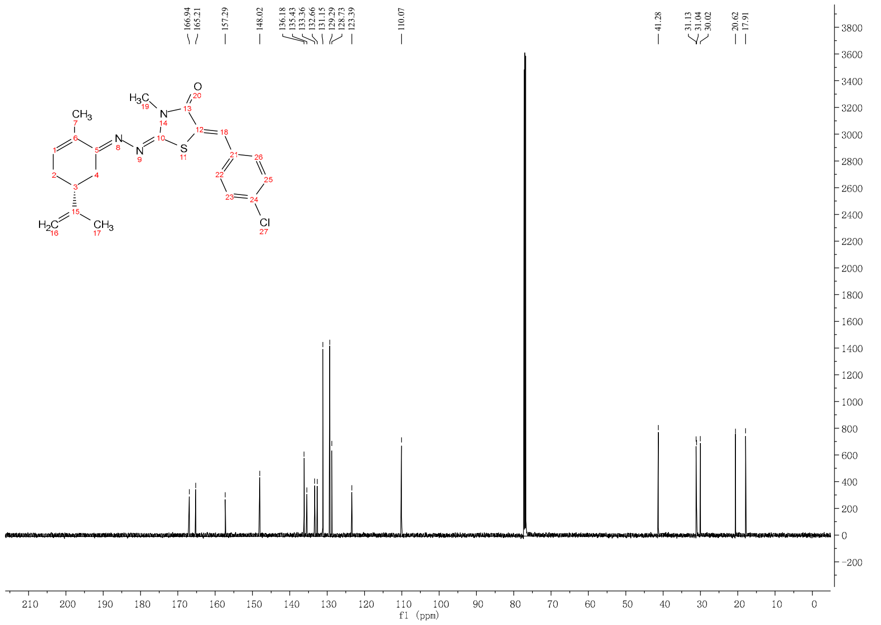

Figure S33 ^{13}C NMR spectrum of compound **4g**.

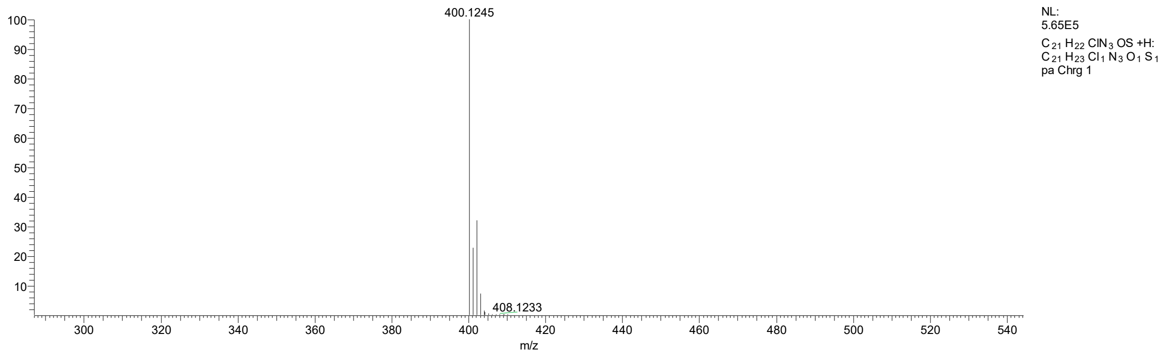

Figure S34 HRMS spectrum of compound **4g**.

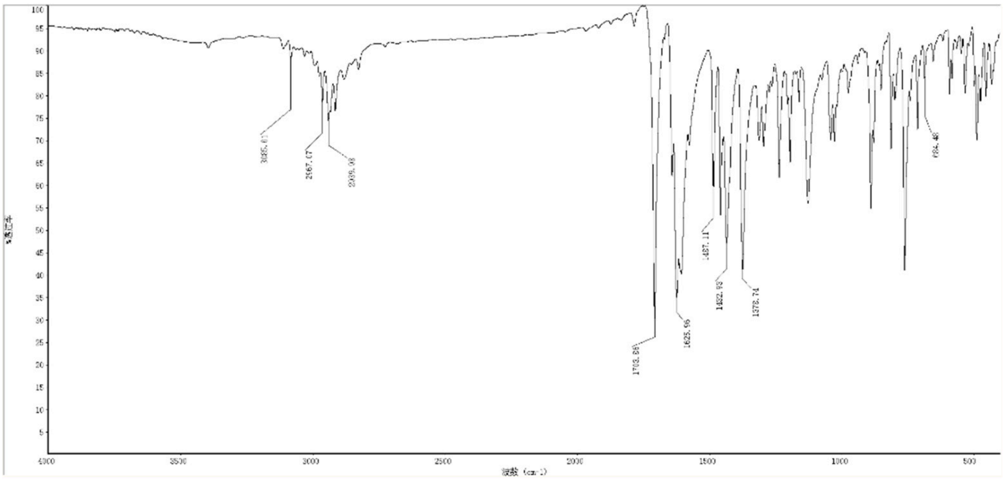

Figure S35 IR spectrum of compound **4h**.

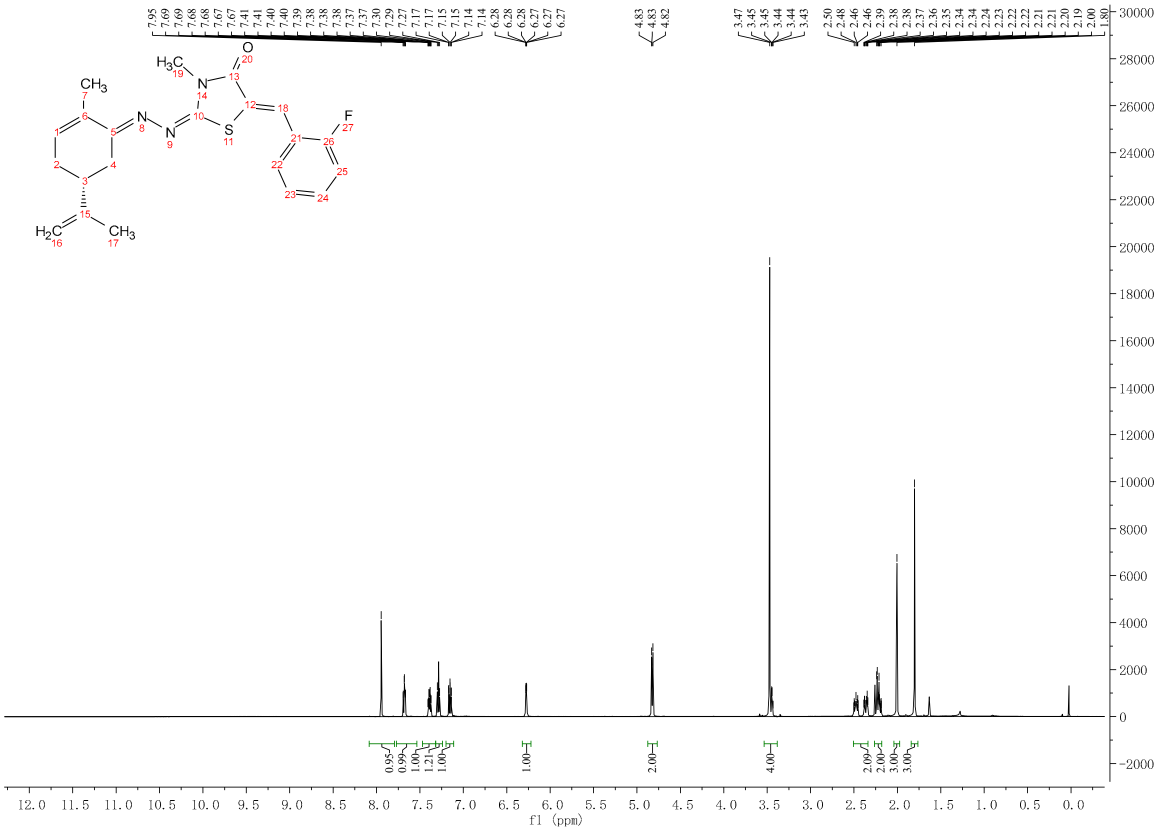

Figure S36 ^{1}H NMR spectrum of compound **4h**.

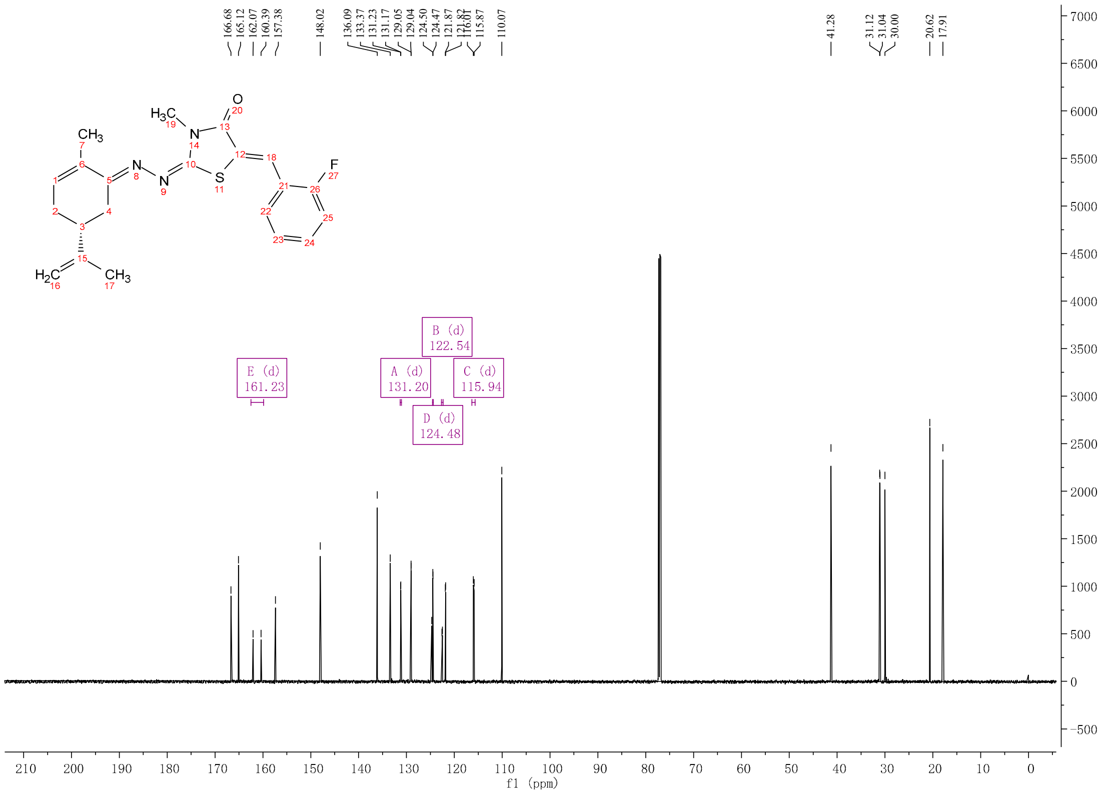

Figure S37 ^{13}C NMR spectrum of compound **4h**.

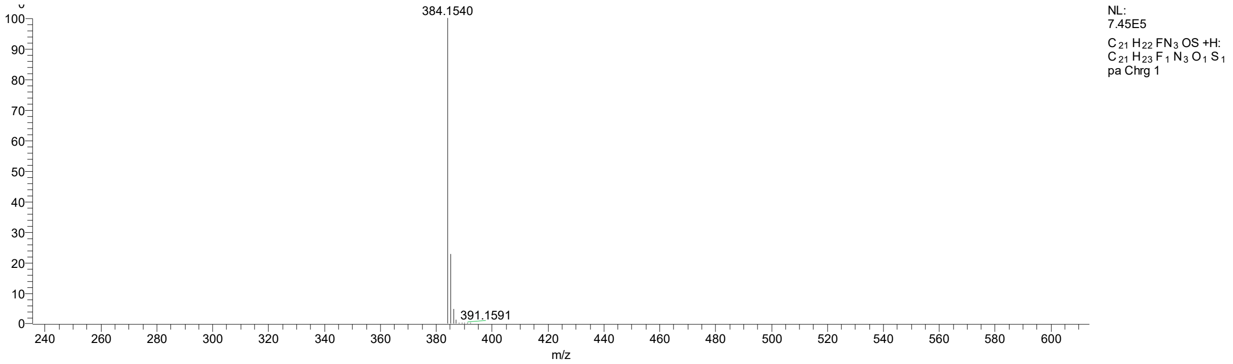

Figure S38 HRMS spectrum of compound **4h**.

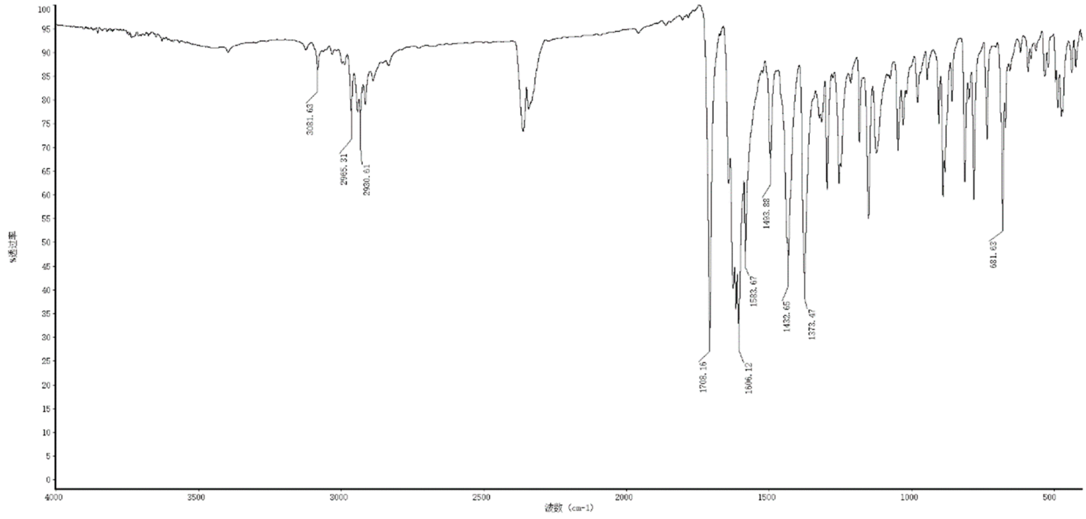

Figure S39 IR spectrum of compound **4i**.

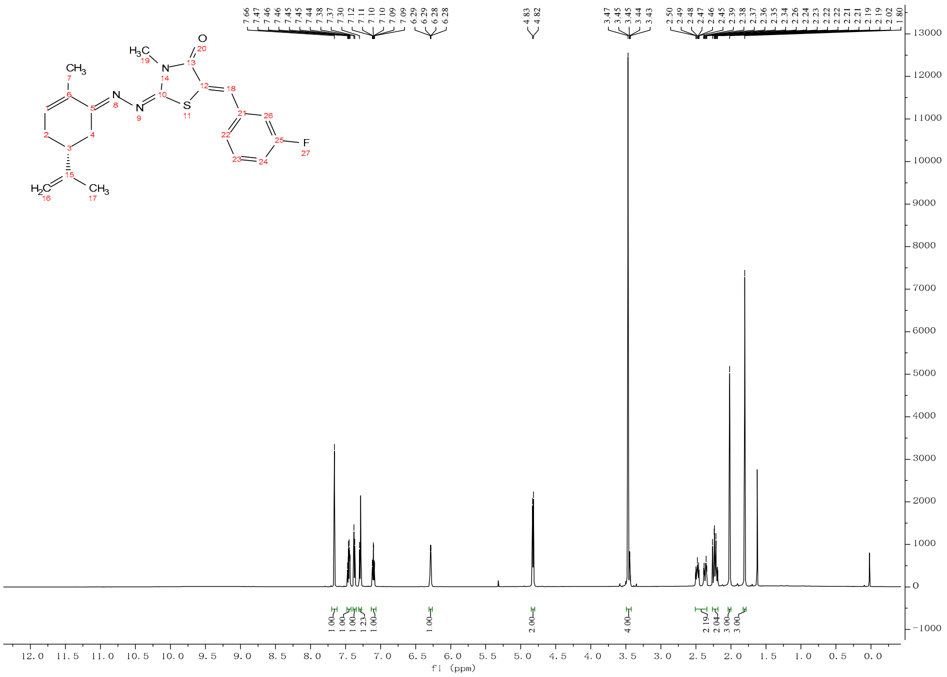

Figure S40 ^{1}H NMR spectrum of compound **4i**.

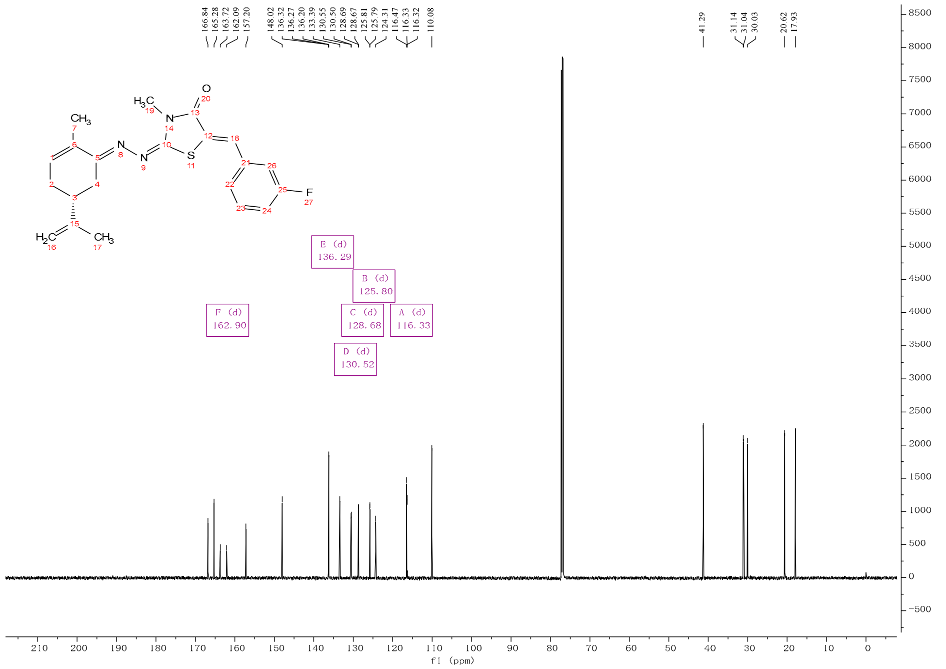

Figure S41 ^{13}C NMR spectrum of compound **4i**.

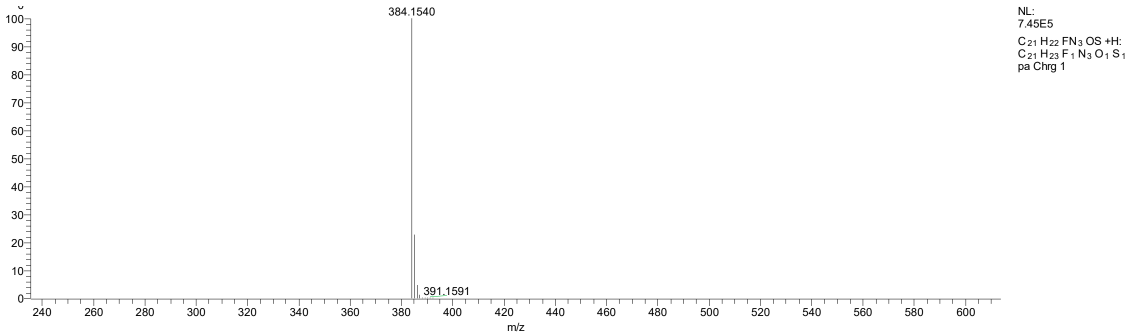

Figure S42 HRMS spectrum of compound **4i**.

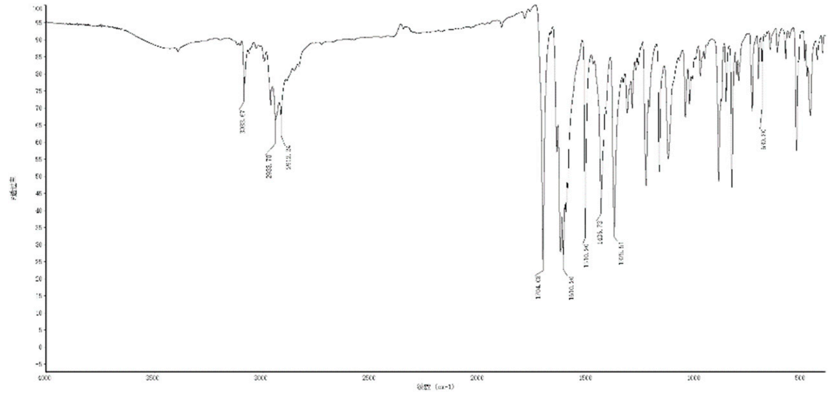

Figure S43 IR spectrum of compound **4j**.

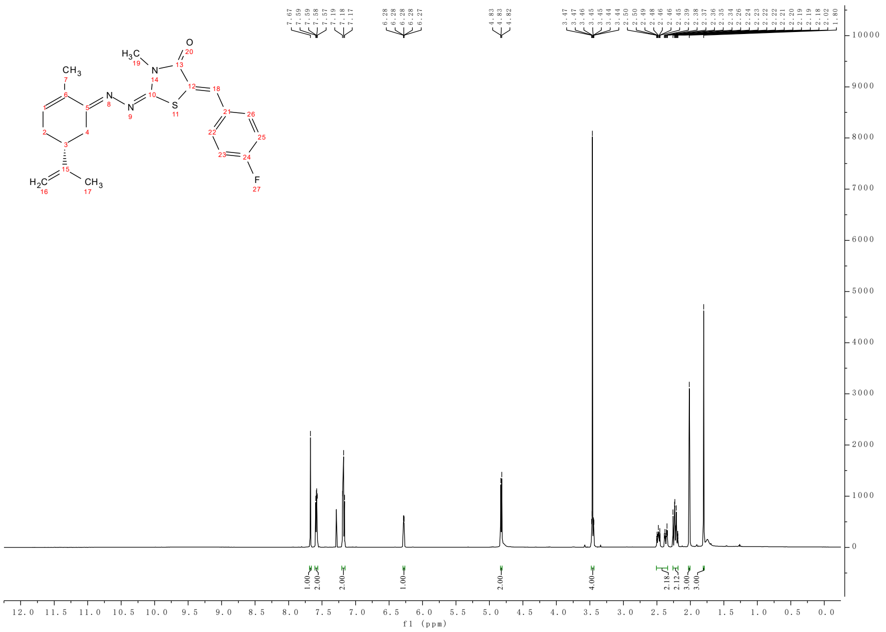

Figure S44 ^{1}H NMR spectrum of compound **4j**.

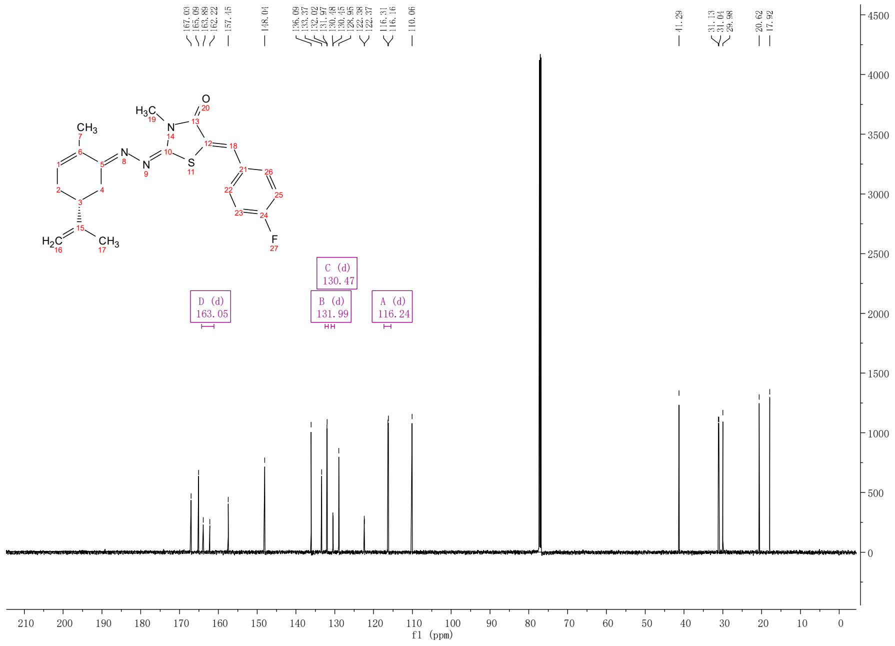

Figure S45 ^{13}C NMR spectrum of compound **4j**.

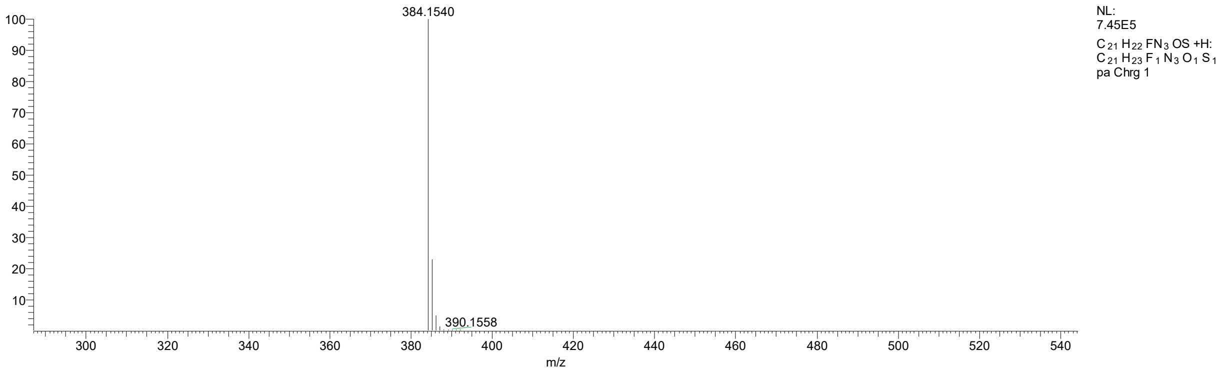

Figure S46 HRMS spectrum of compound **4j**.

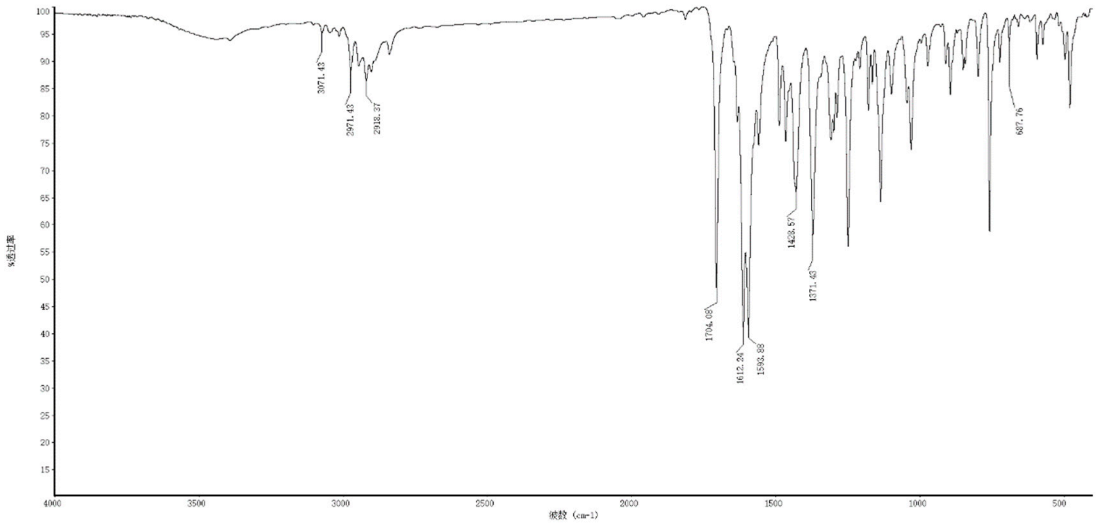

Figure S47 IR spectrum of compound **4k**.

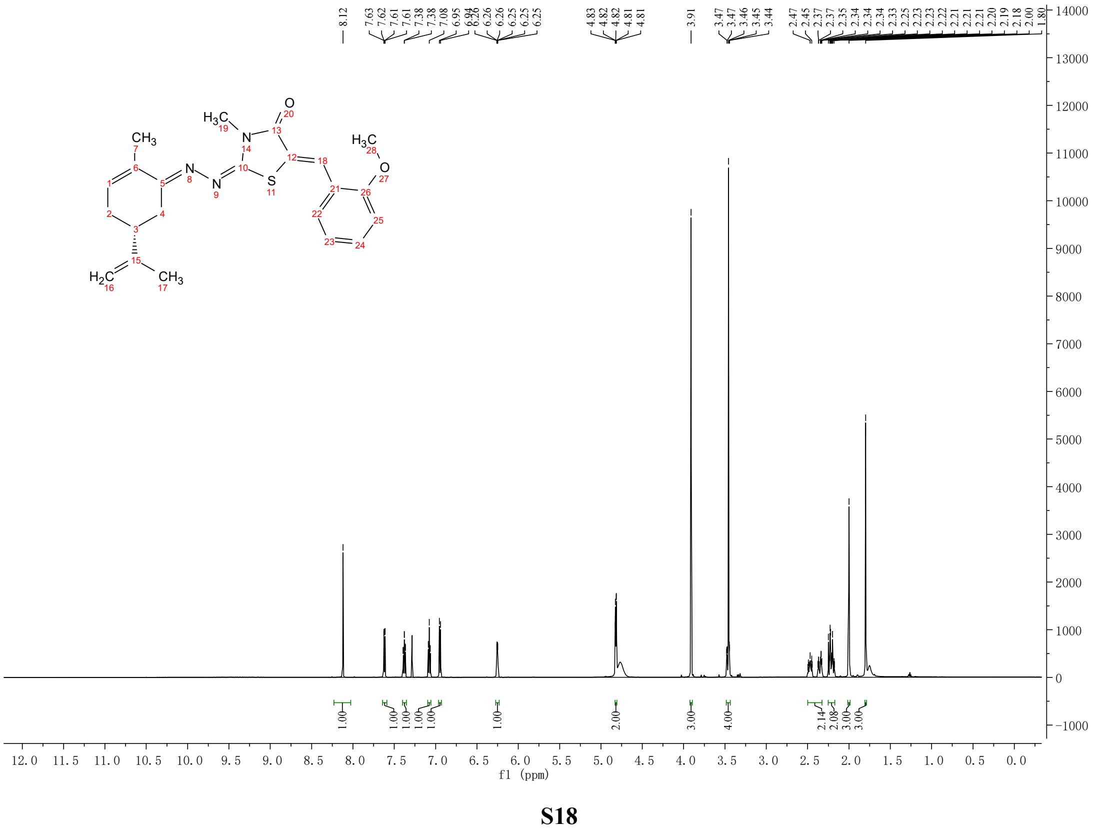

Figure S48 ^{1}H NMR spectrum of compound **4k**.

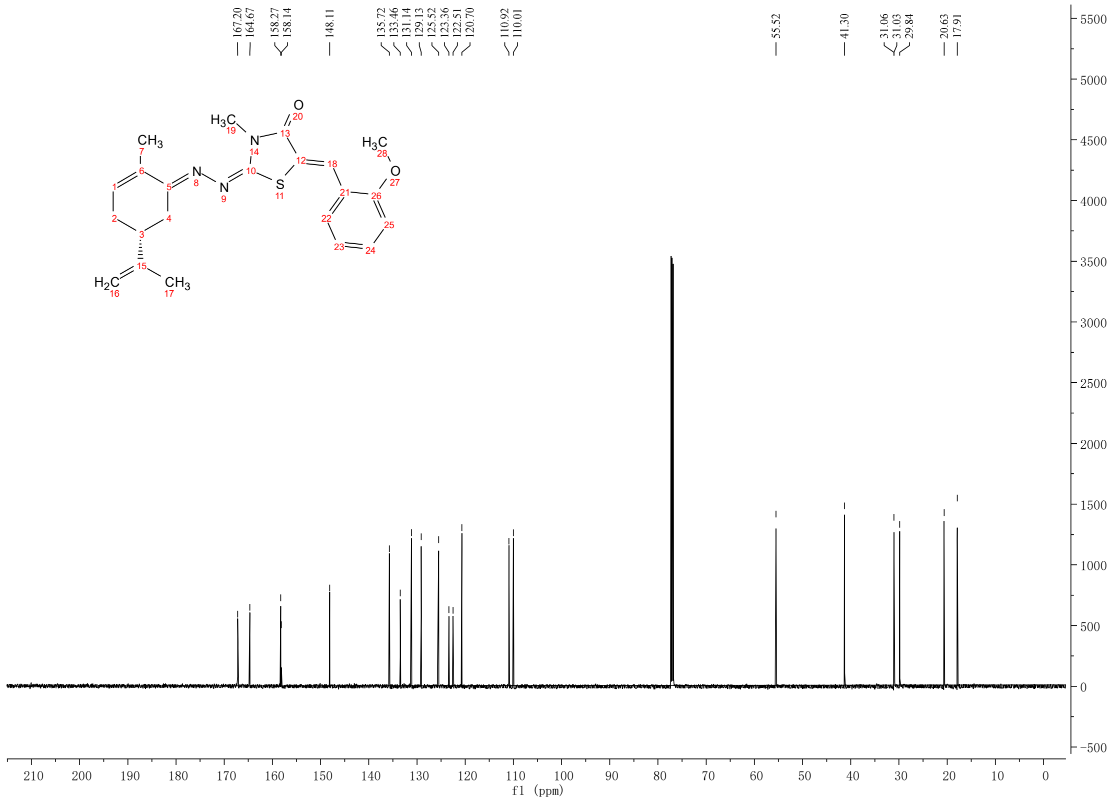

Figure S49 ^{13}C NMR spectrum of compound **4k**.

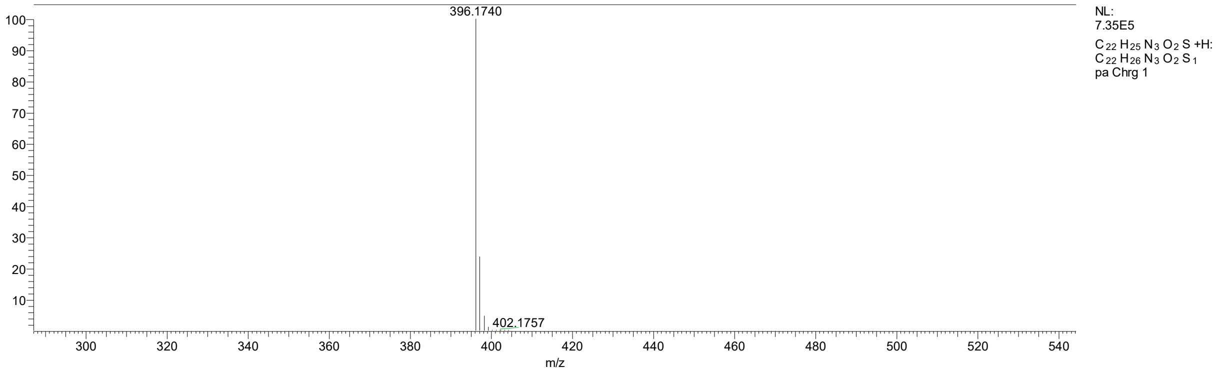

Figure S50 HRMS spectrum of compound **4k**.

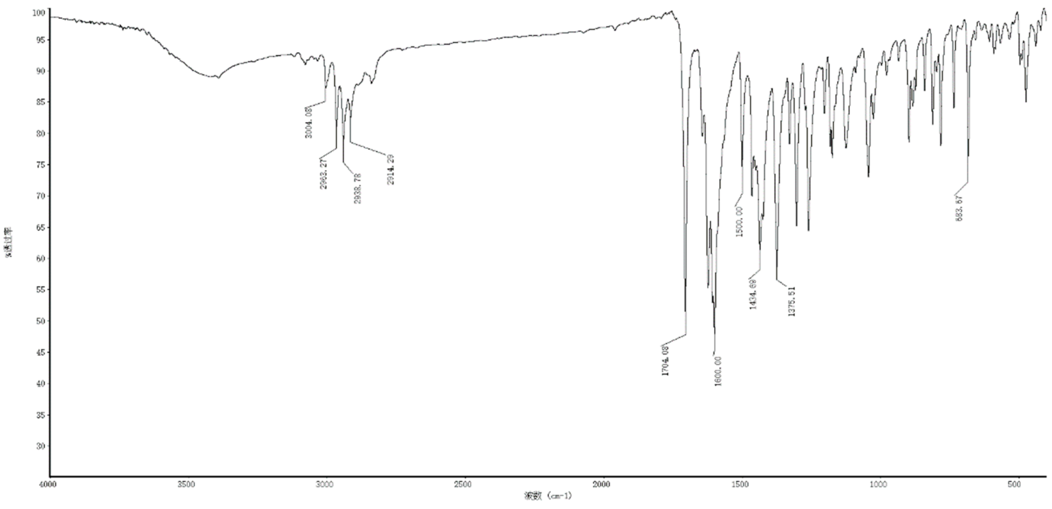

Figure S51 IR spectrum of compound **4l**.

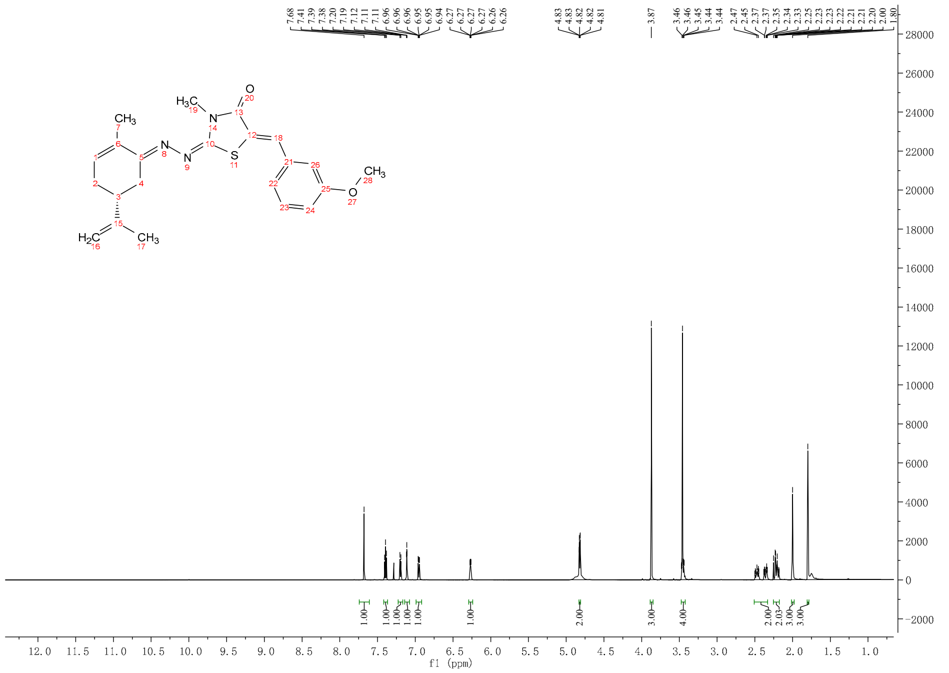

Figure S52 ^{1}H NMR spectrum of compound **4l**.

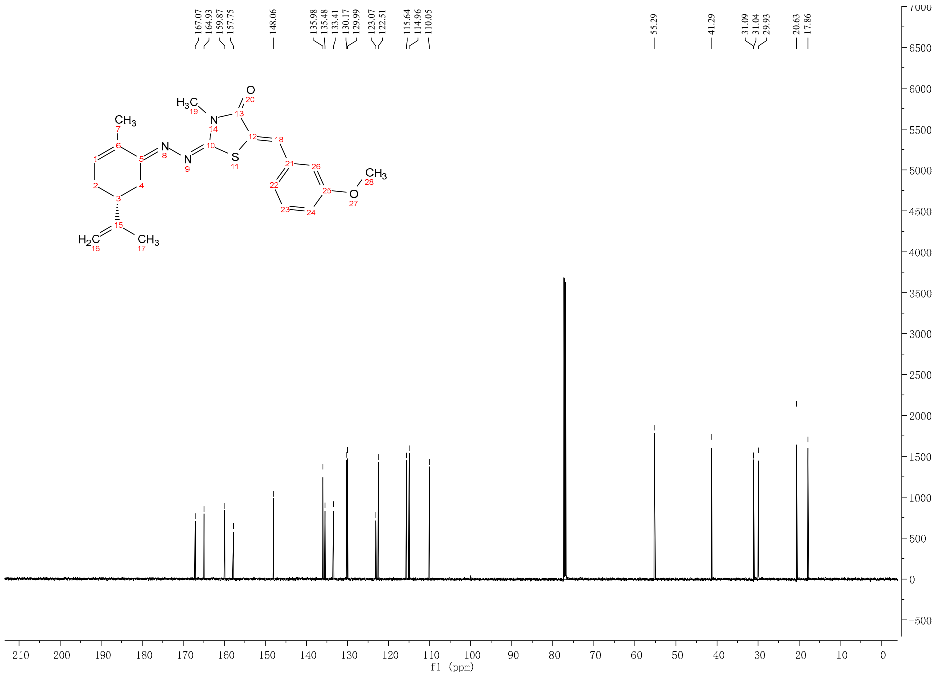

Figure S53 ^{13}C NMR spectrum of compound **4l**.

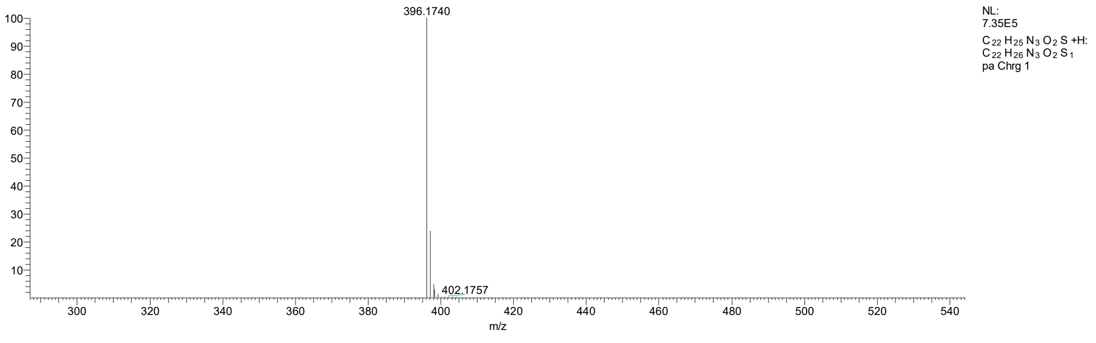

Figure S54 HRMS spectrum of compound **4l**.

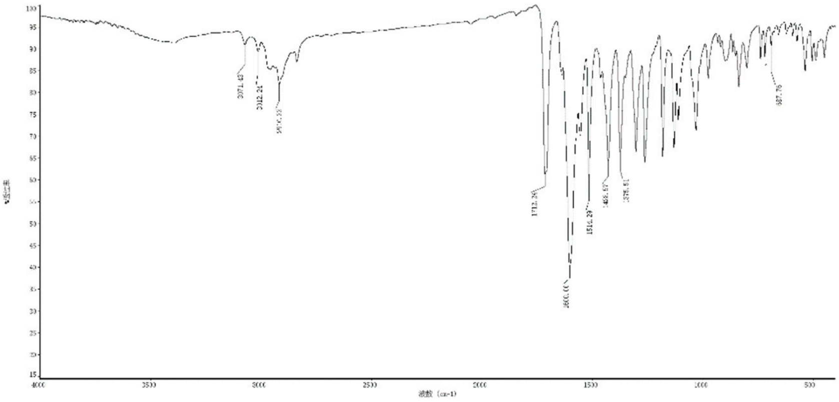

Figure S55 IR spectrum of compound **4m**.

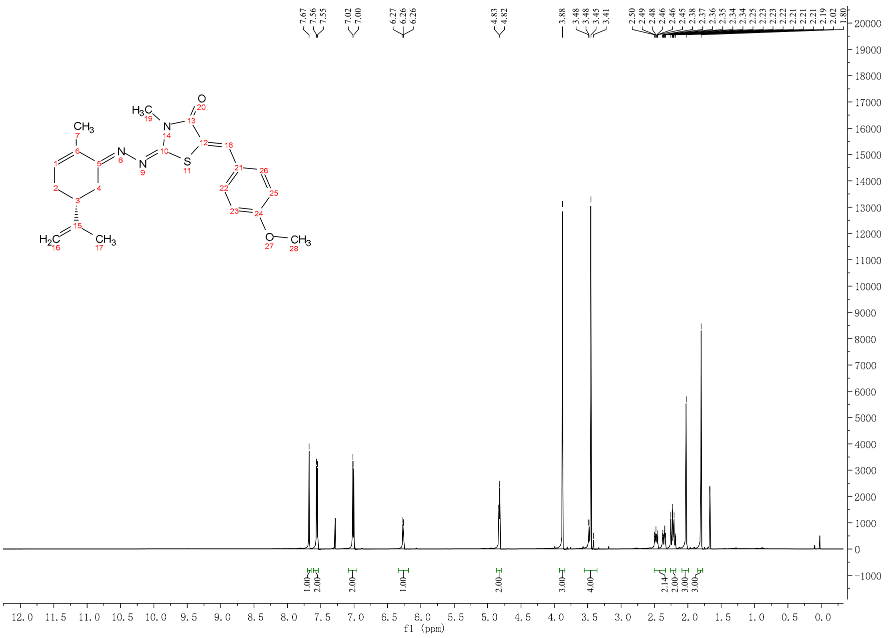

Figure S56 ^{1}H NMR spectrum of compound **4m**.

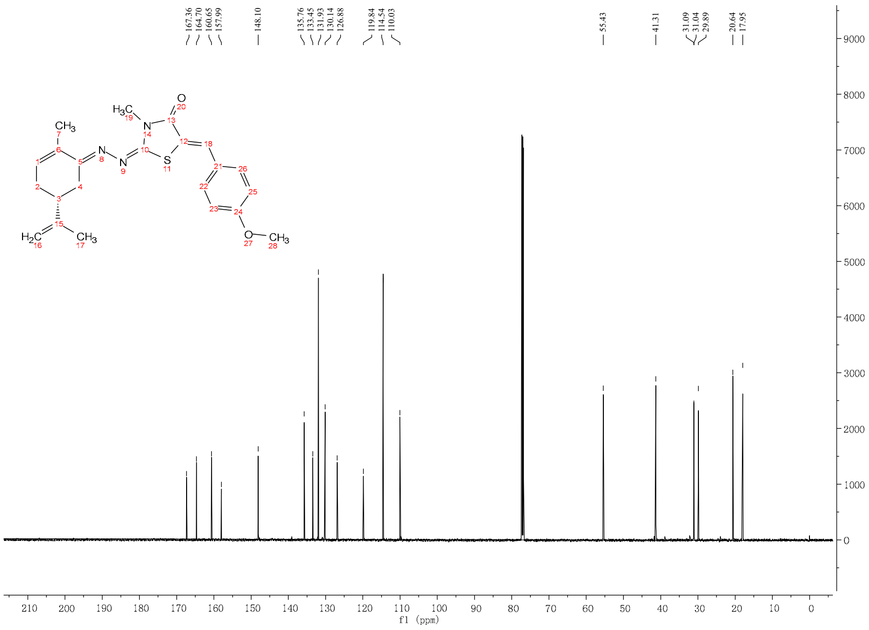

Figure S57 ^{13}C NMR spectrum of compound **4m**.

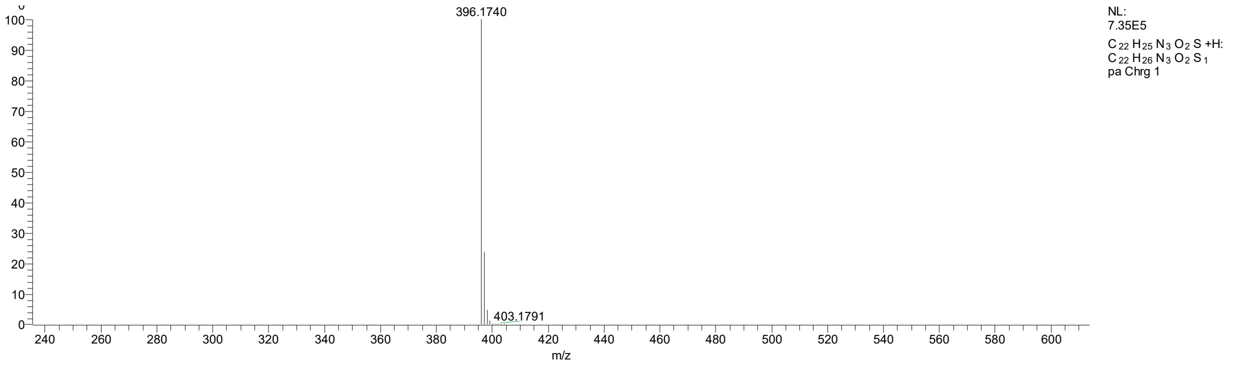

Figure S58 HRMS spectrum of compound **4m**.

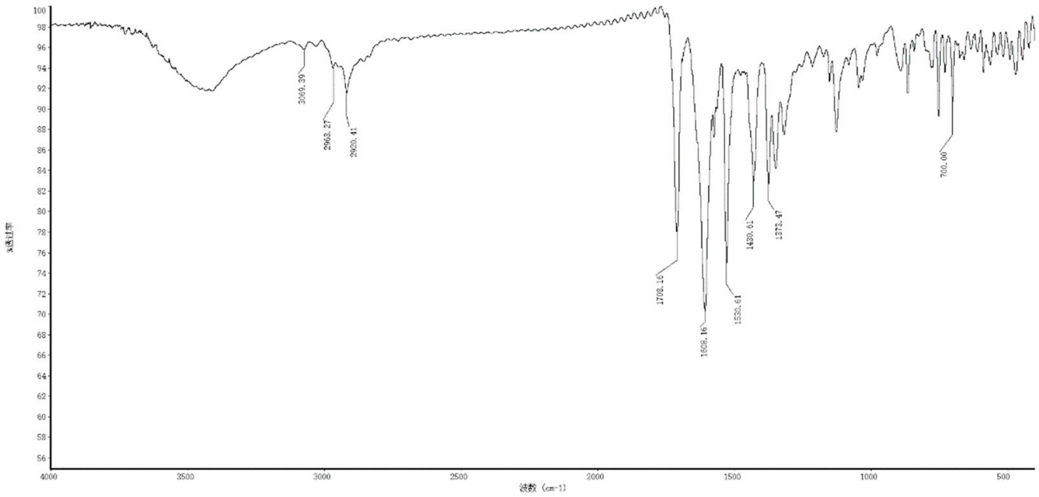

Figure S59 IR spectrum of compound **4n**.

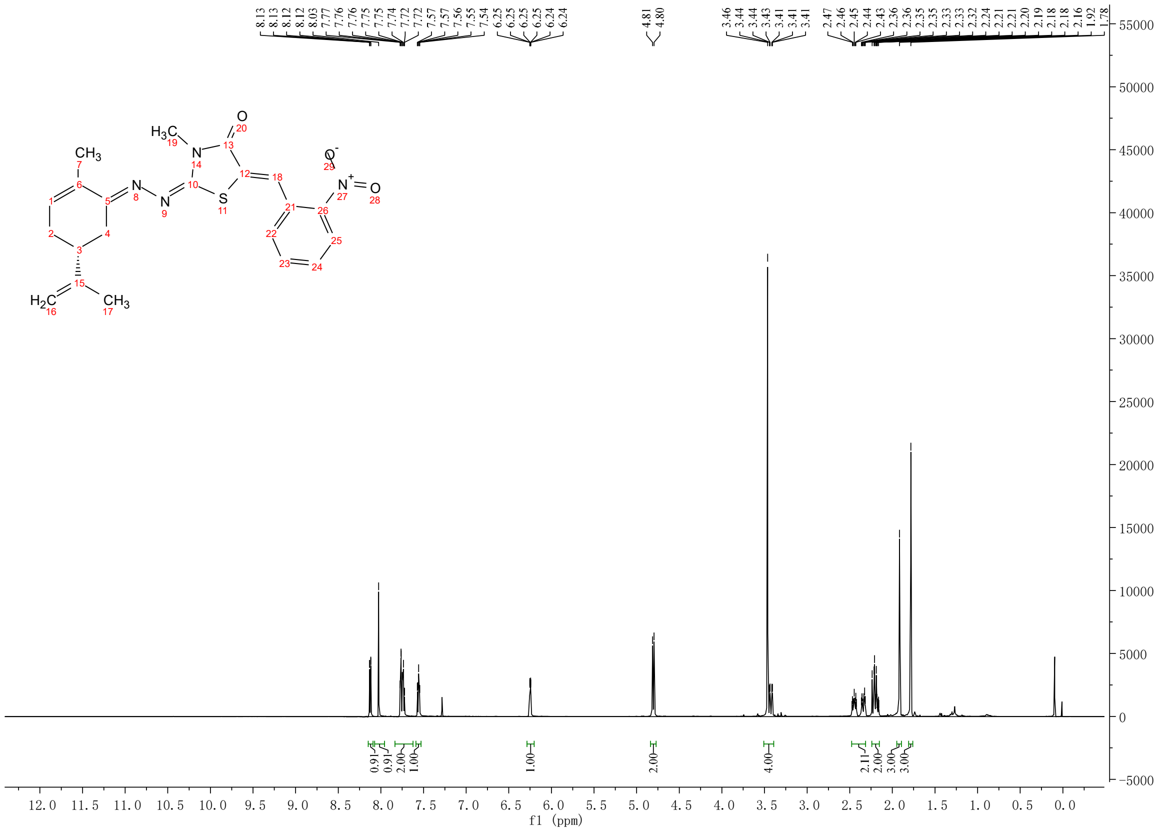

Figure S60 ^{1}H NMR spectrum of compound **4n**.

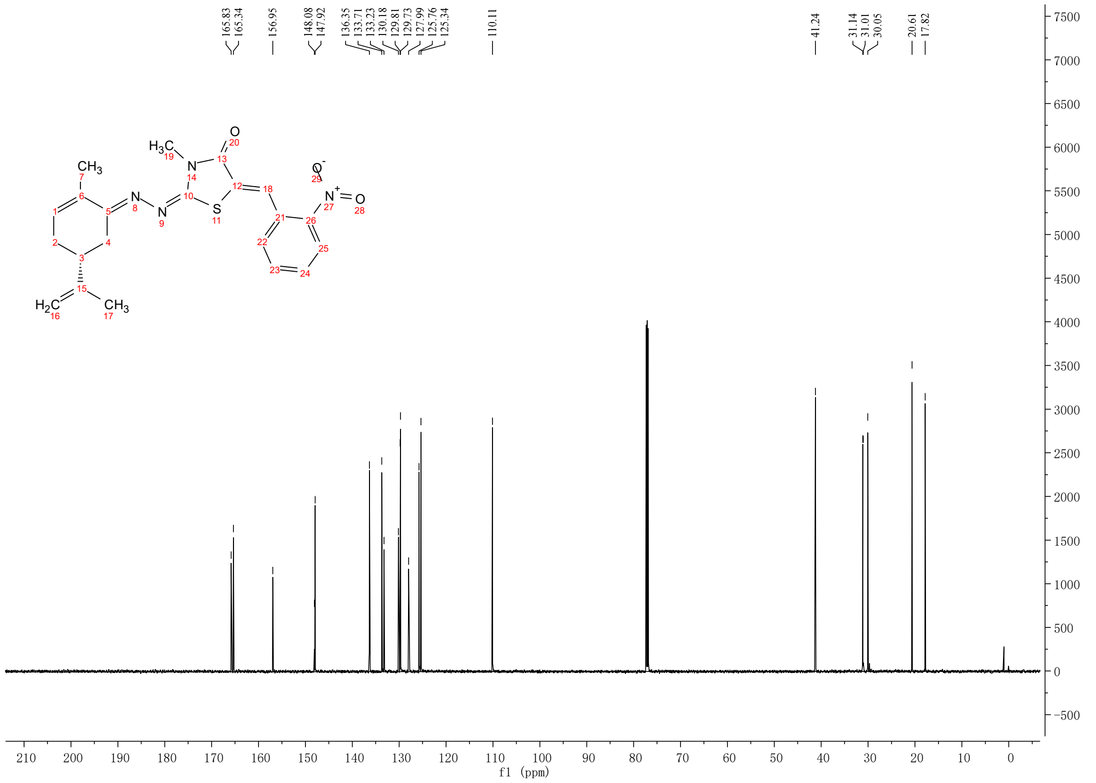

Figure S61 ^{13}C NMR spectrum of compound **4n**.

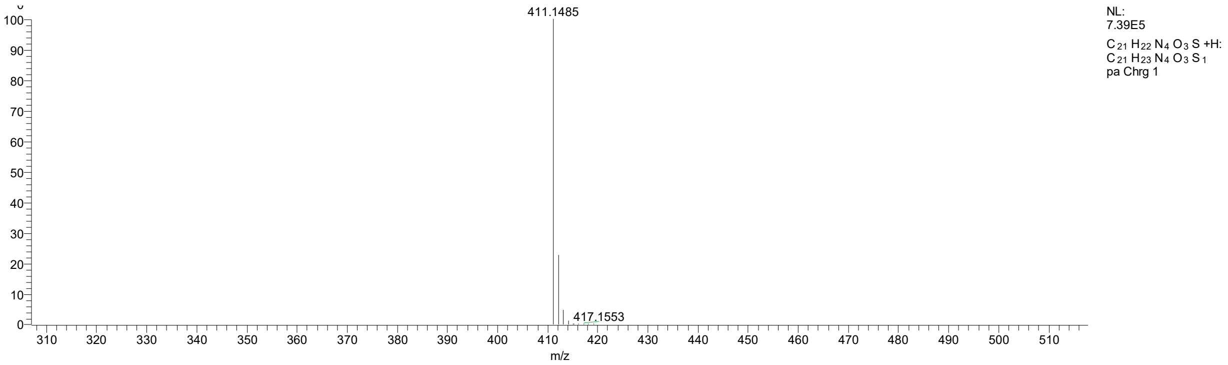

Figure S62 HRMS spectrum of compound **4n**.

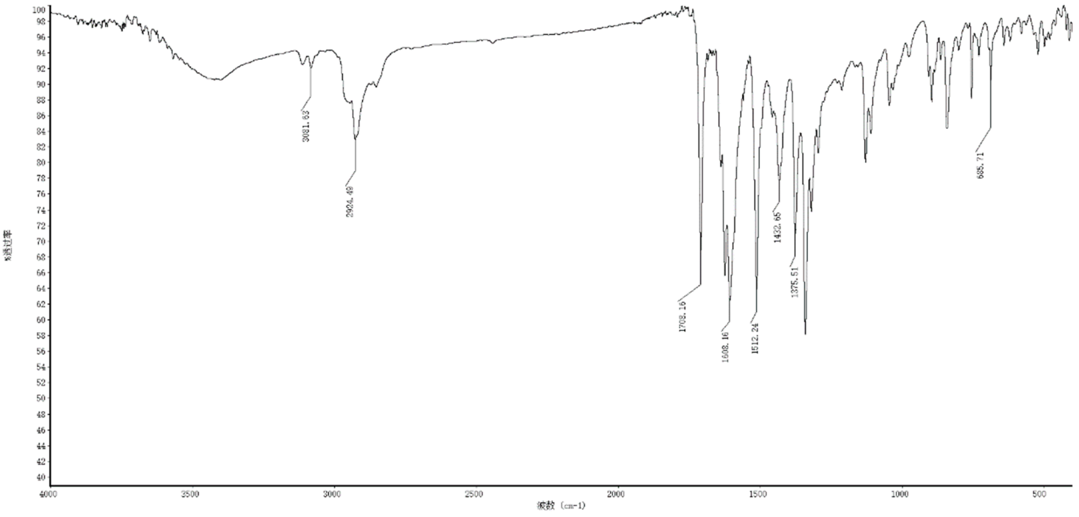

Figure S63 IR spectrum of compound **4o**.

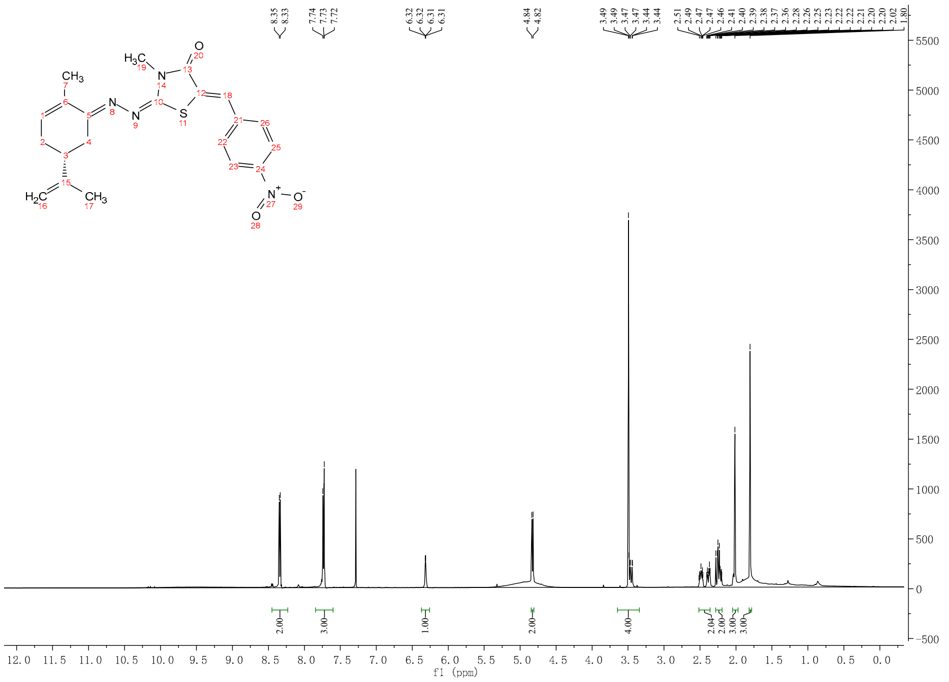

Figure S64 ^{1}H NMR spectrum of compound **4o**.

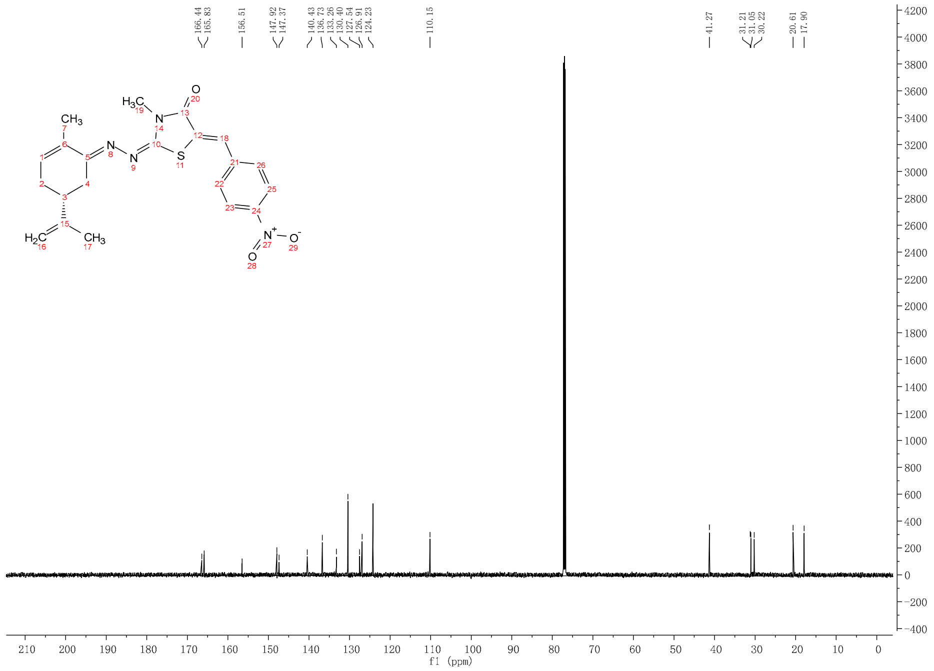

Figure S65 ^{13}C NMR spectrum of compound **4o**.

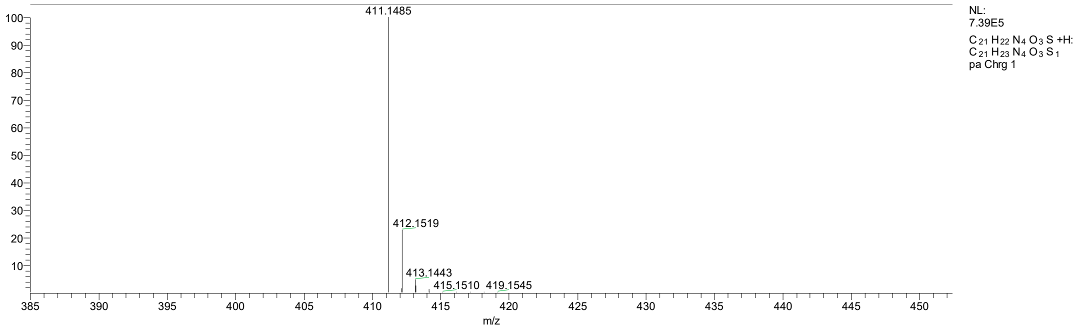

Figure S66 HRMS spectrum of compound **4o**.

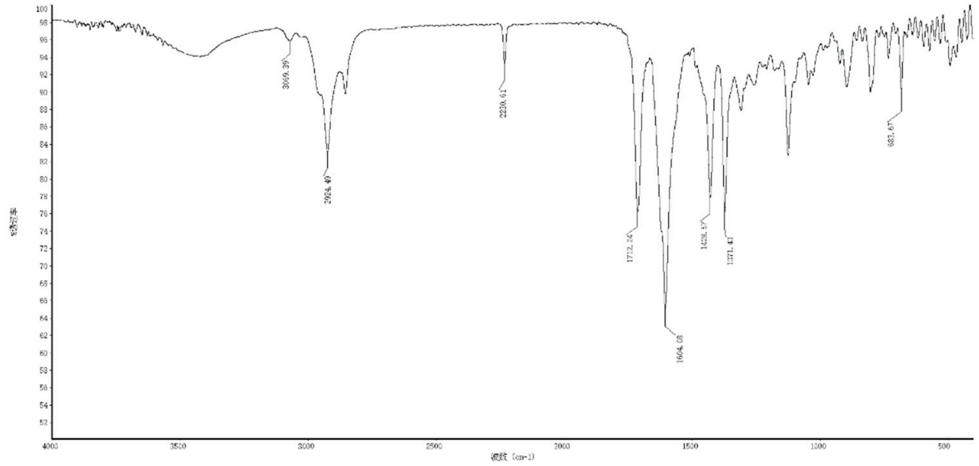

Figure S67 IR spectrum of compound **4p**.

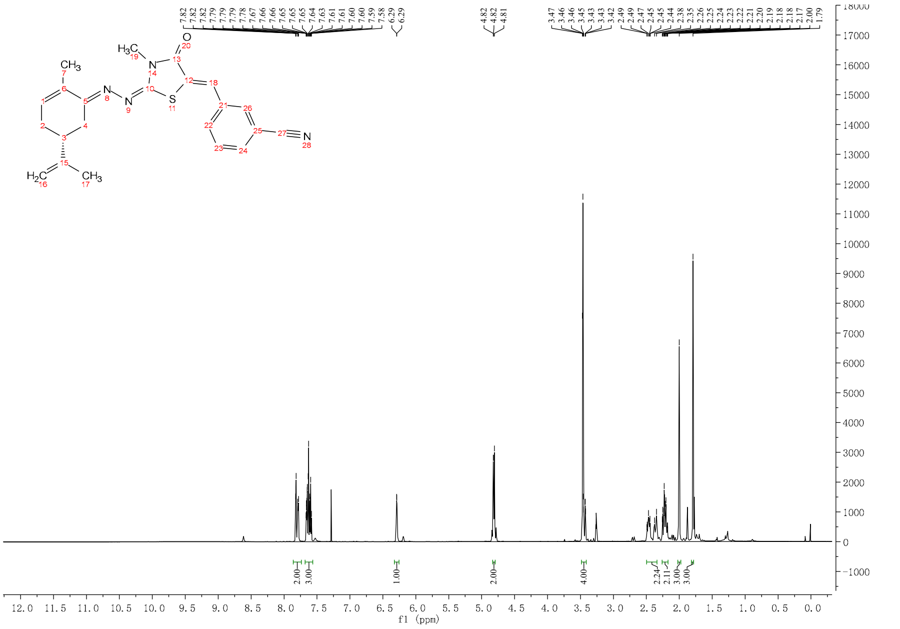

Figure S68 ^{1}H NMR spectrum of compound **4p**.

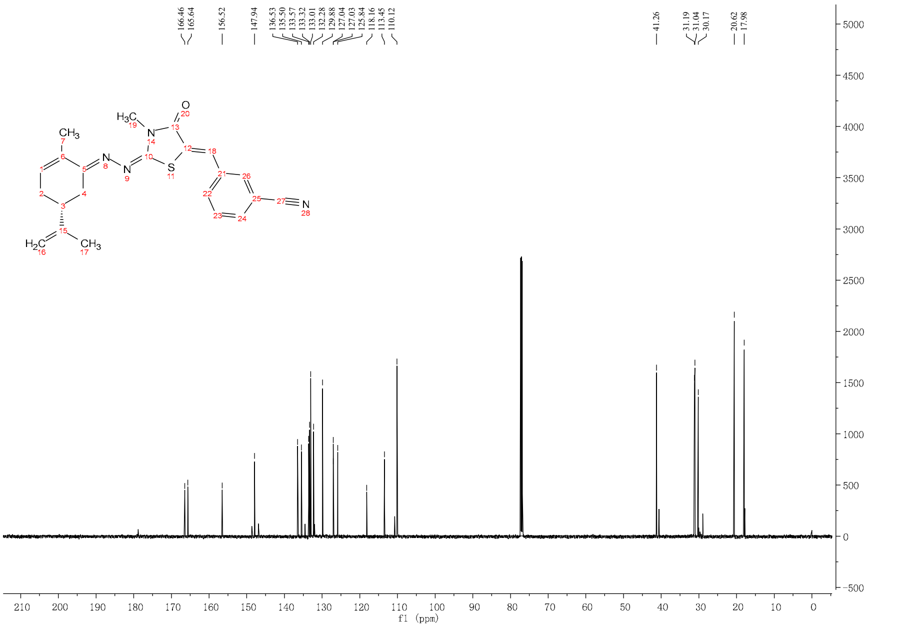

Figure S69 ^{13}C NMR spectrum of compound **4p**.

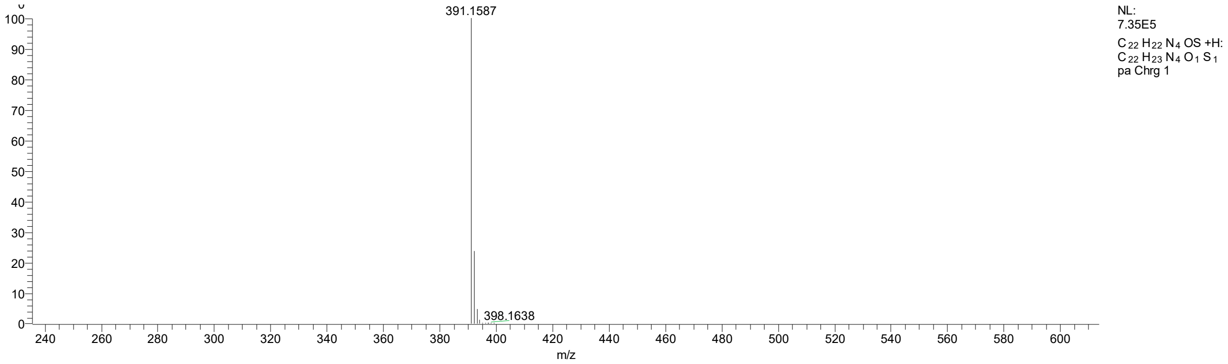

Figure S70 HRMS spectrum of compound **4p**.

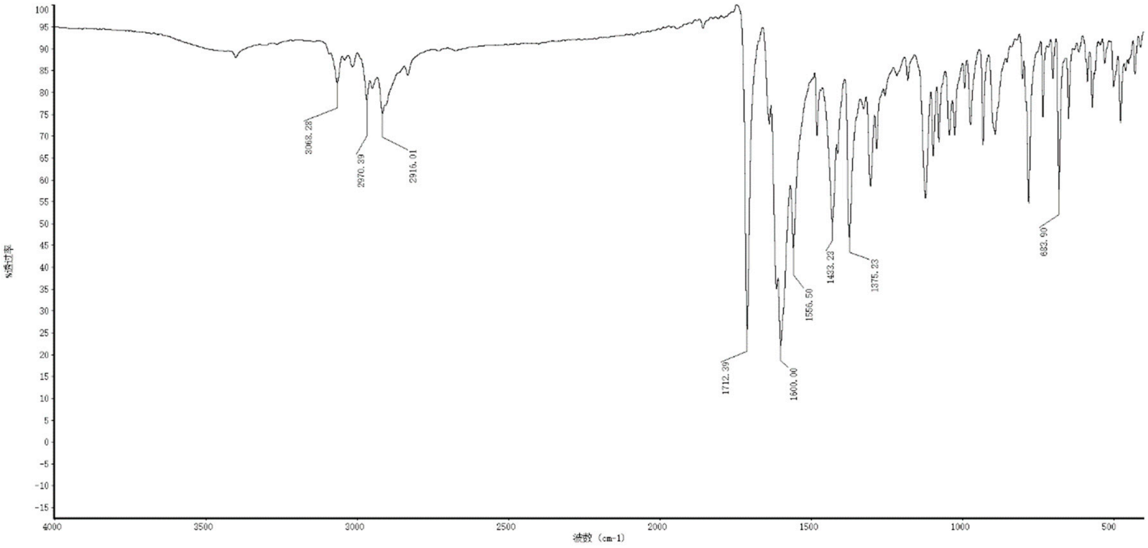

Figure S71 IR spectrum of compound **4q**.

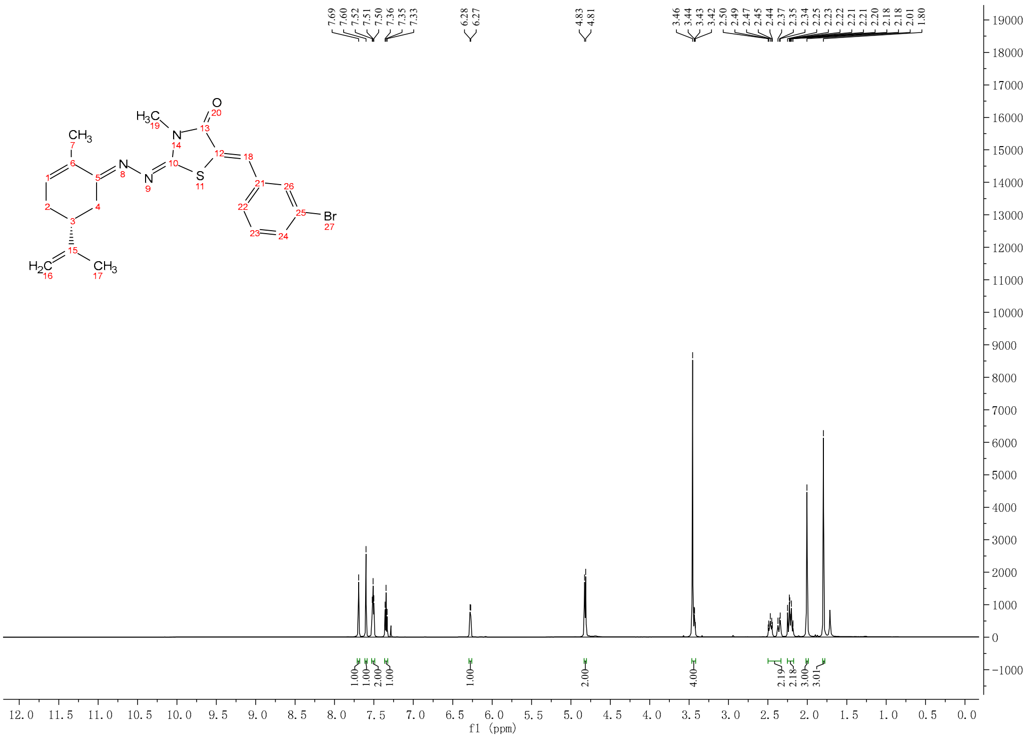

Figure S72 ^{1}H NMR spectrum of compound **4q**.

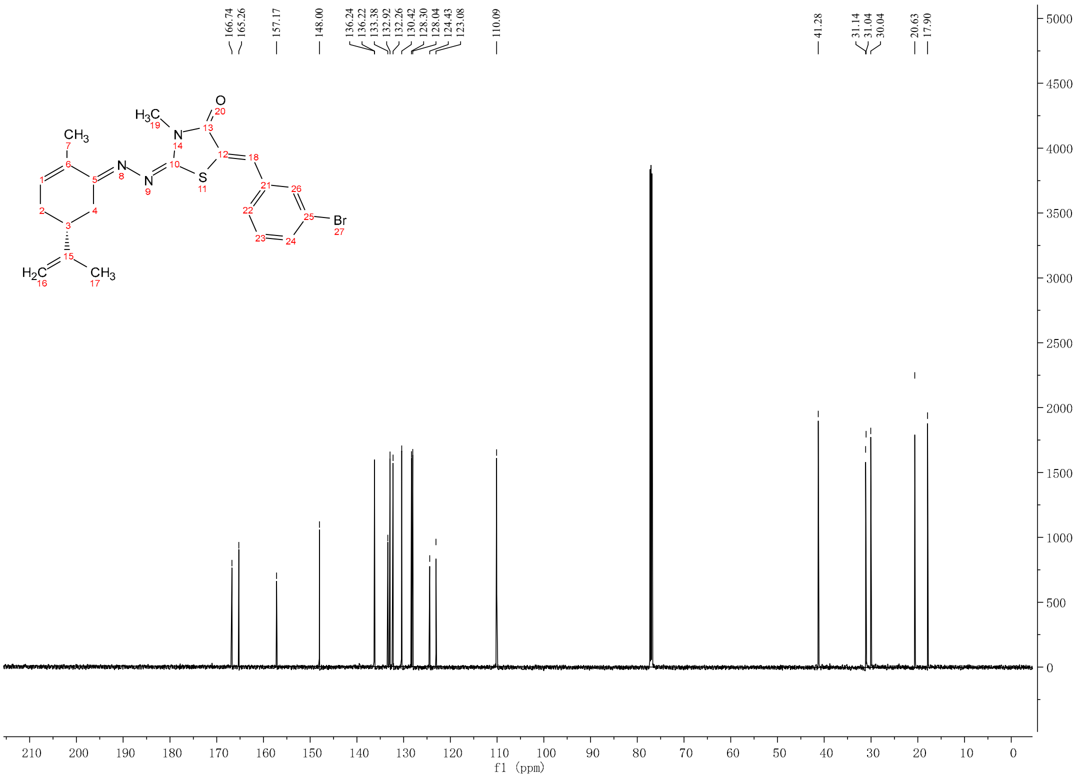

Figure S73 ^{13}C NMR spectrum of compound **4q**.

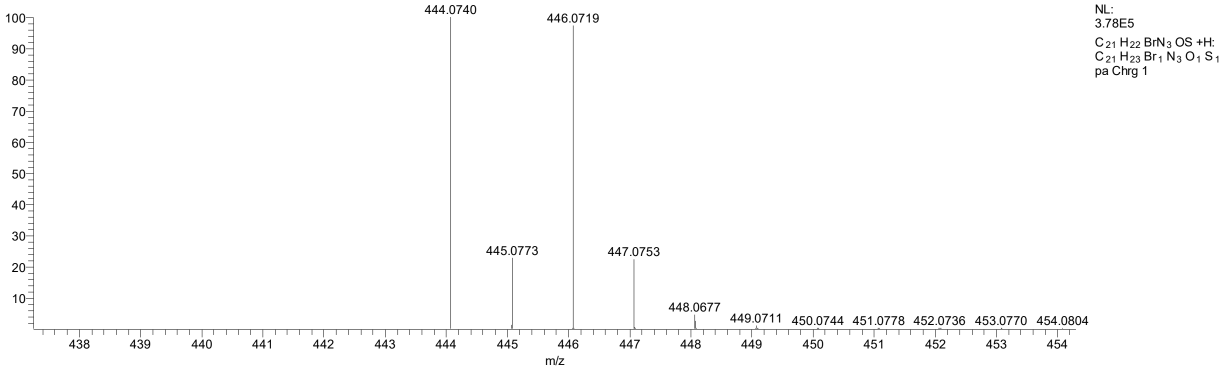

Figure S74 HRMS spectrum of compound **4q**.

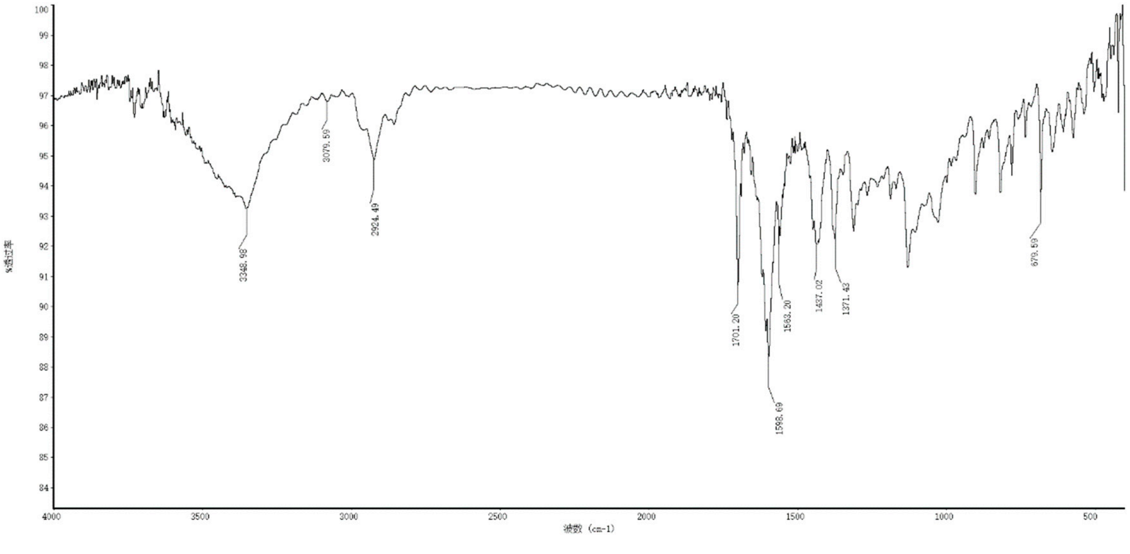

Figure S75 IR spectrum of compound **4r**.

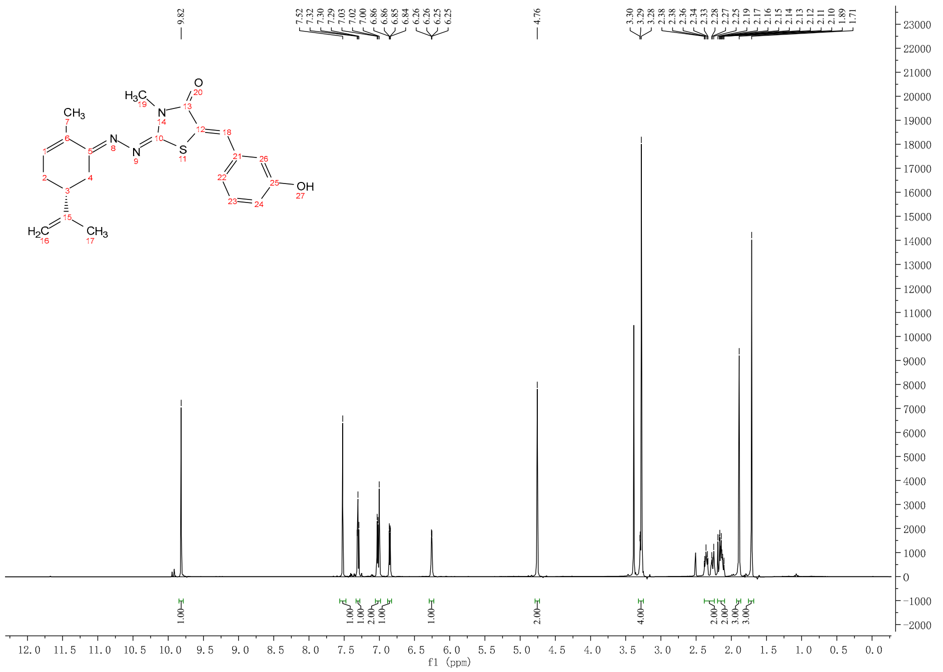

Figure S76 ^{1}H NMR spectrum of compound **4r**.

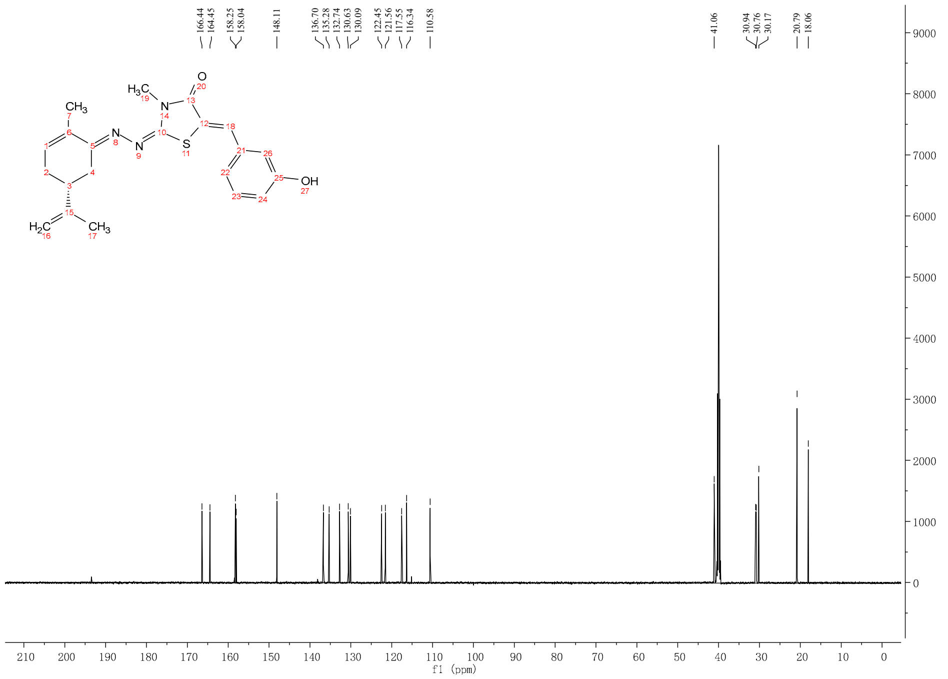

Figure S77 ^{13}C NMR spectrum of compound **4r**.

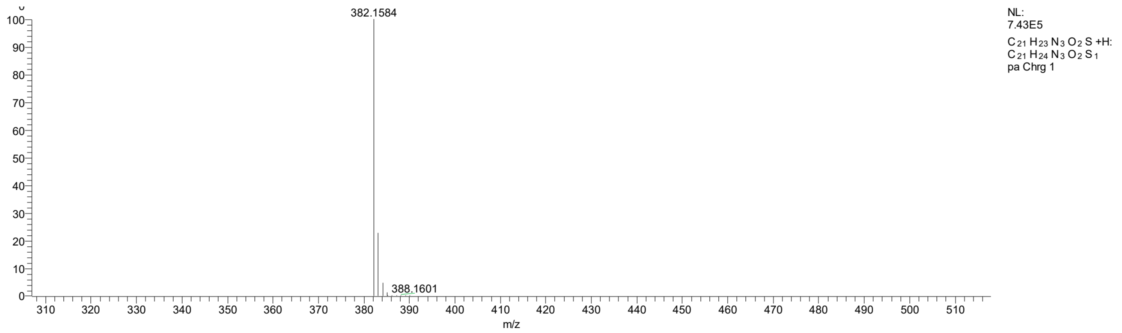

Figure S78 HRMS spectrum of compound **4r**.

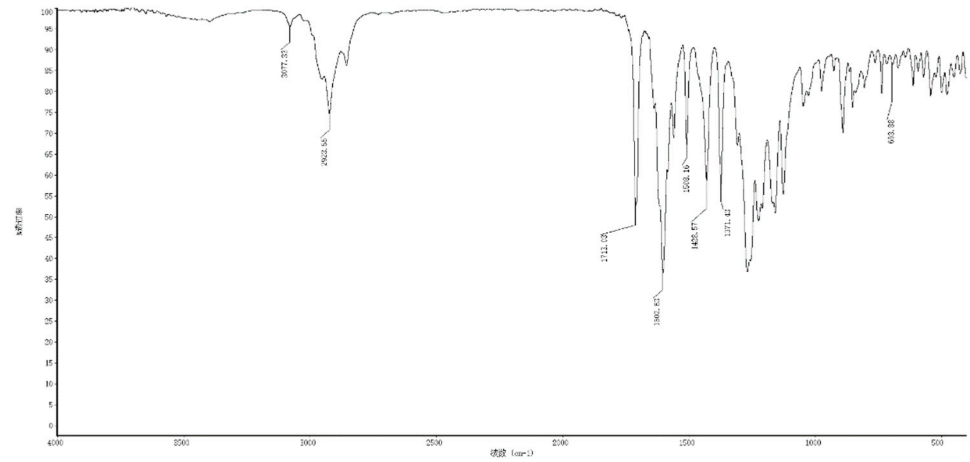

Figure S79 IR spectrum of compound **4s**.

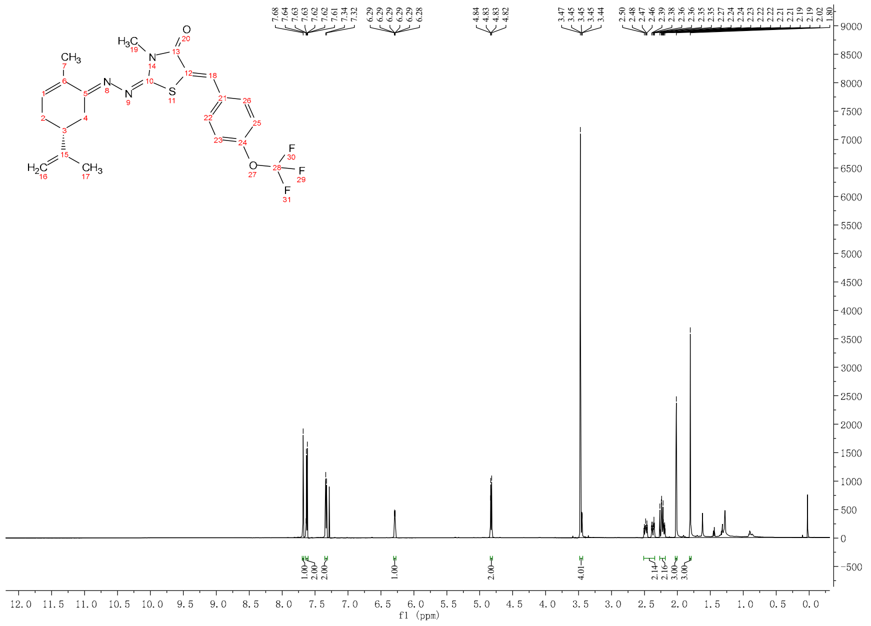

Figure S80 ^{1}H NMR spectrum of compound **4s**.

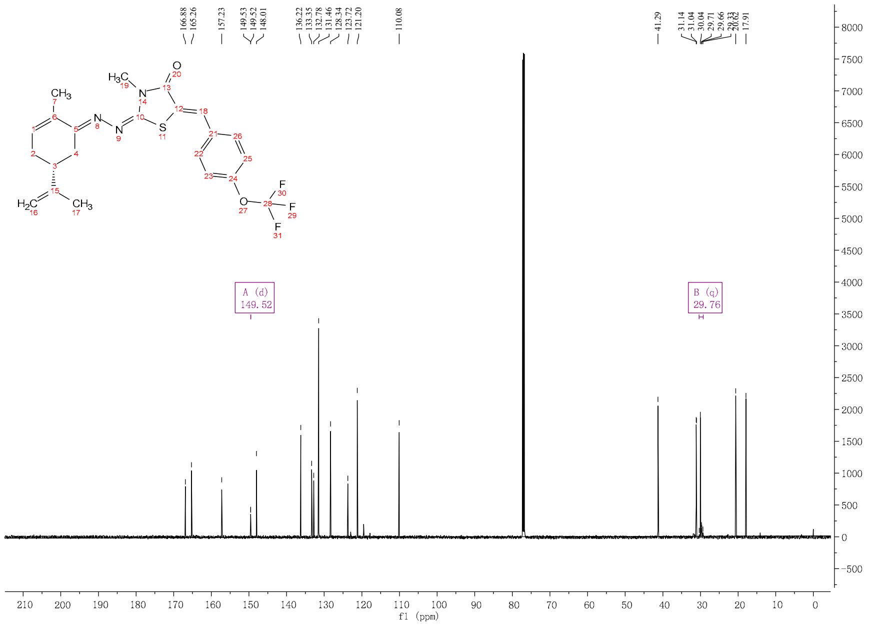

Figure S81 ^{13}C NMR spectrum of compound **4s**.

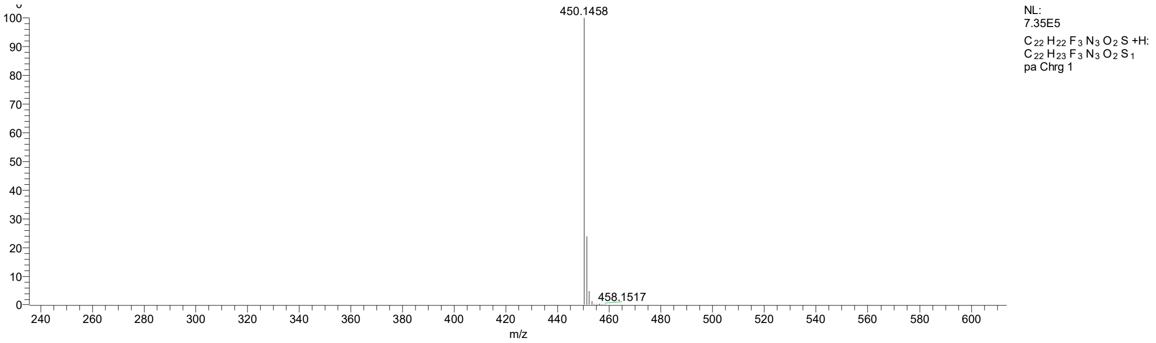

Figure S82 HRMS spectrum of compound **4s**.

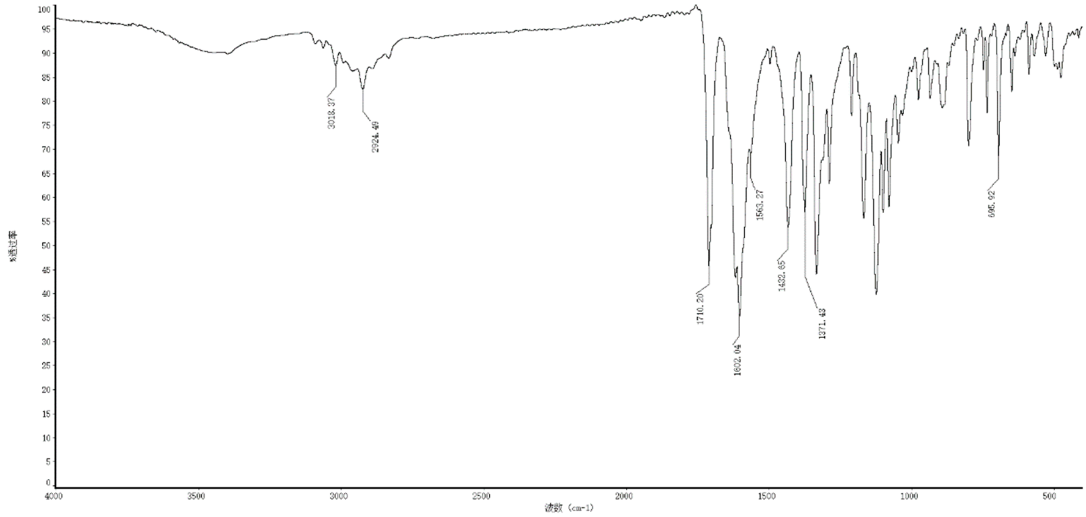

Figure S83 IR spectrum of compound **4t**.

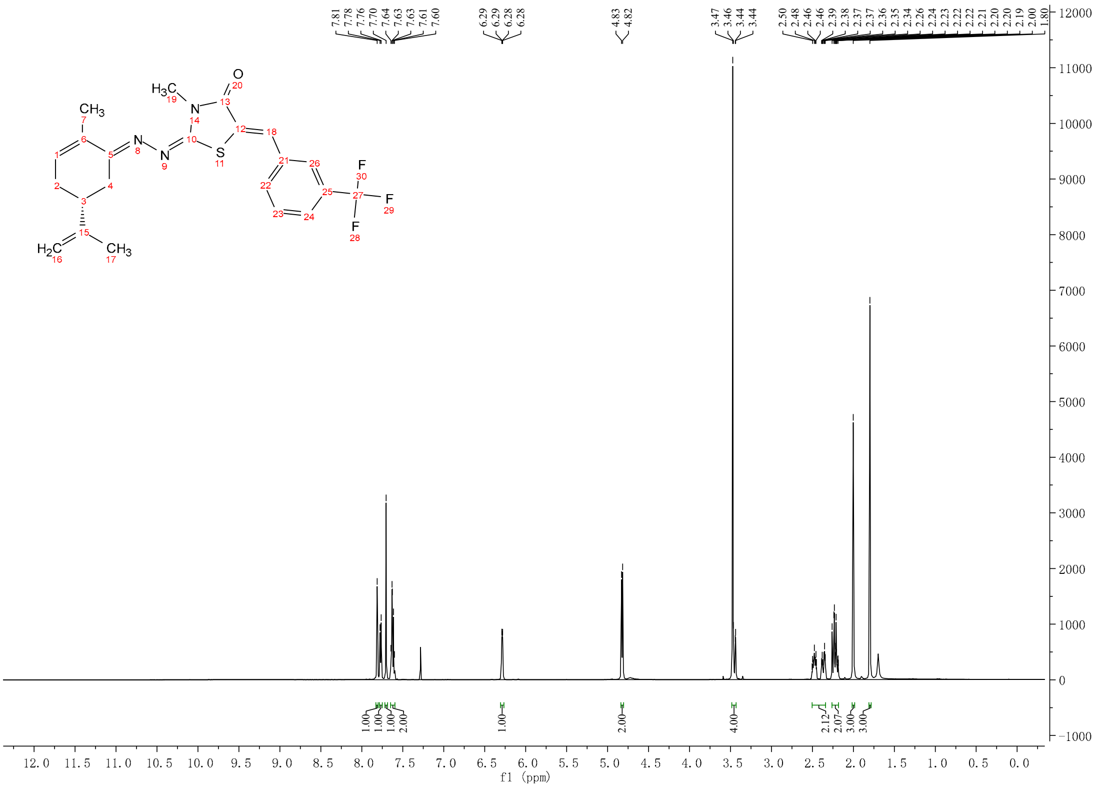

Figure S84 ^{1}H NMR spectrum of compound **4t**.

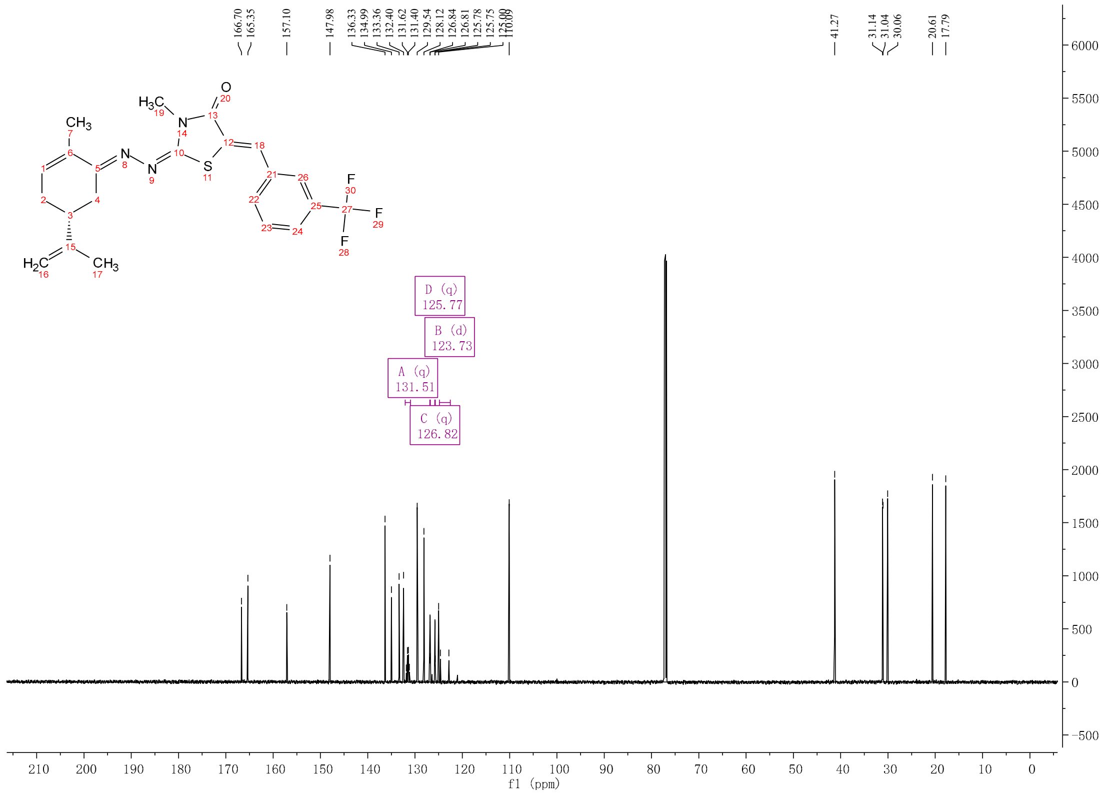

Figure S85 ^{13}C NMR spectrum of compound **4t**.

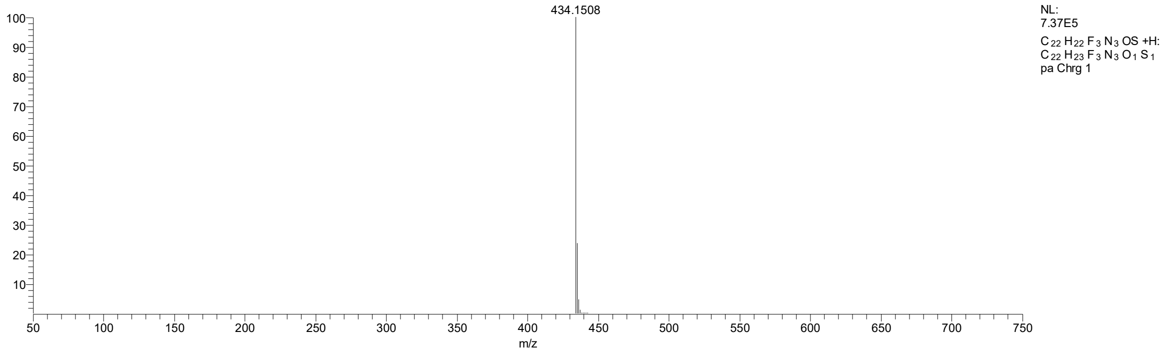

Figure S86 HRMS spectrum of compound **4t**.

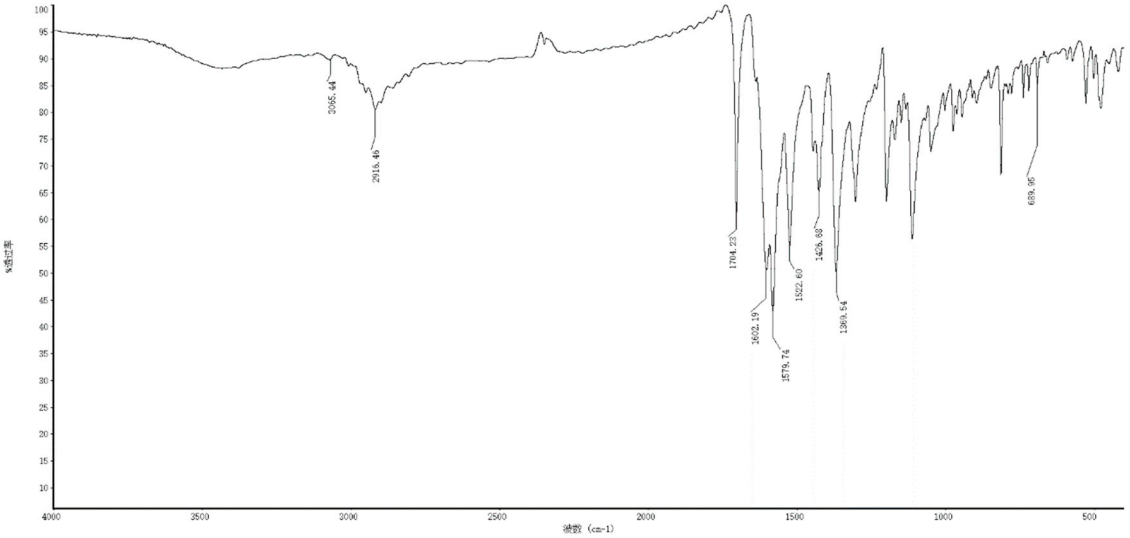

Figure S87 IR spectrum of compound **4u**.

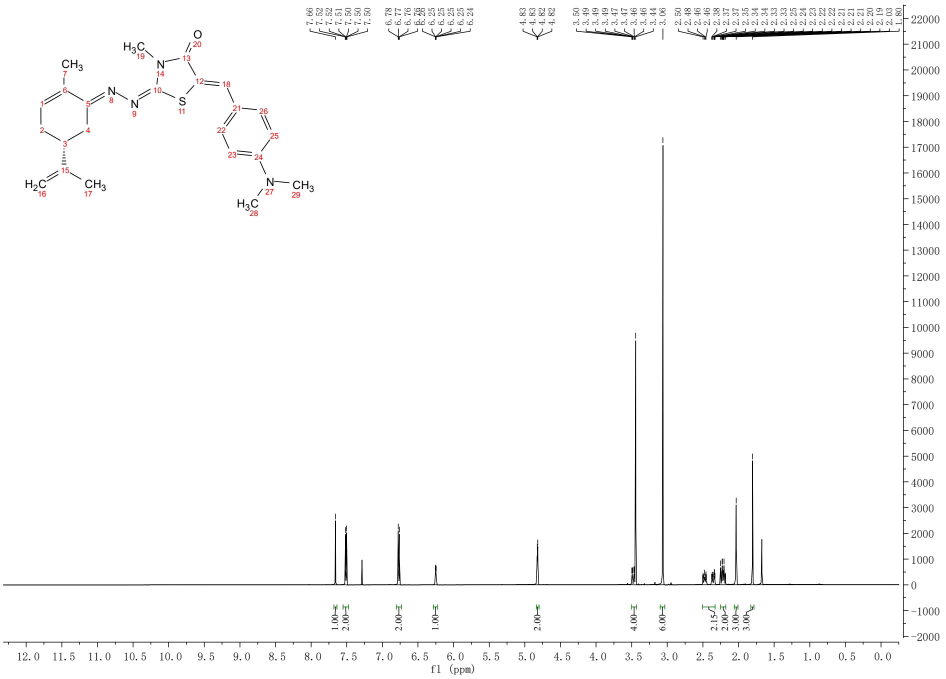

Figure S88 ^{1}H NMR spectrum of compound **4u**.

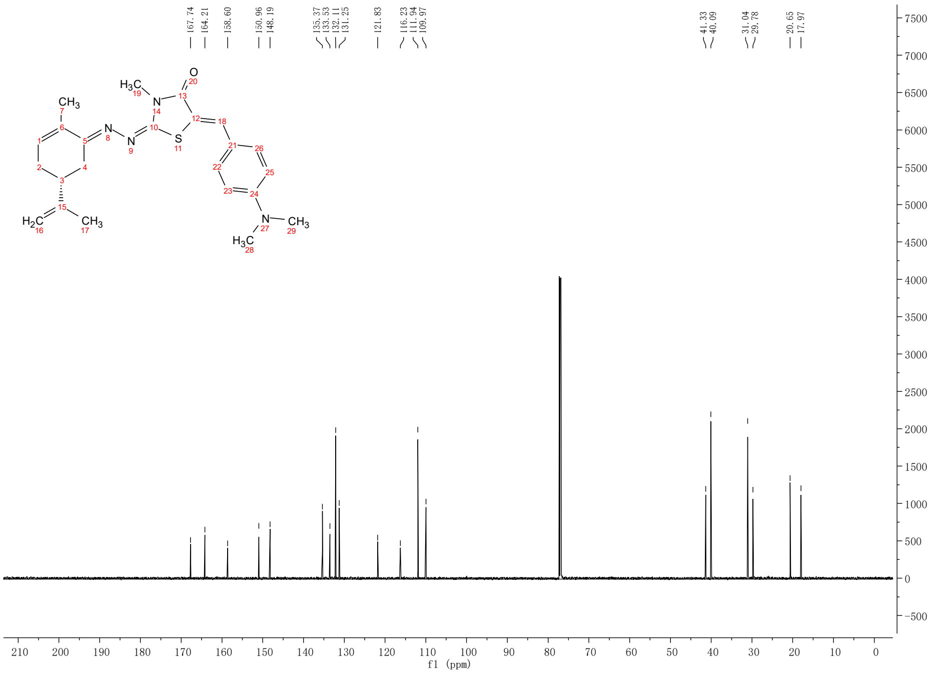

Figure S89 ^{13}C NMR spectrum of compound **4u**.

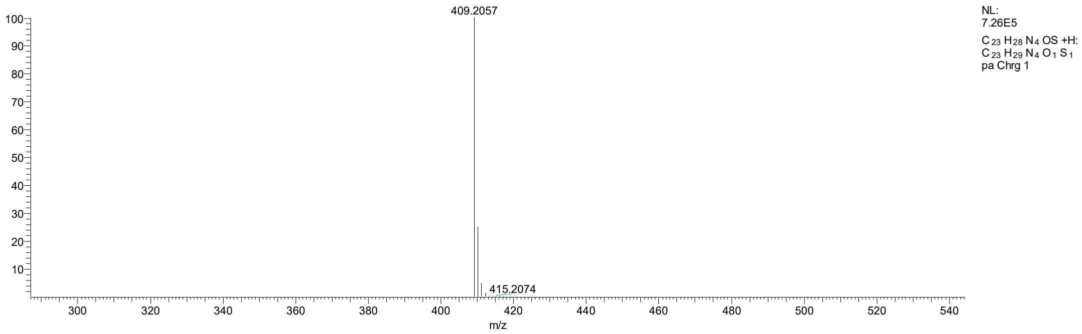

Figure S90 HRMS spectrum of compound **4u**.

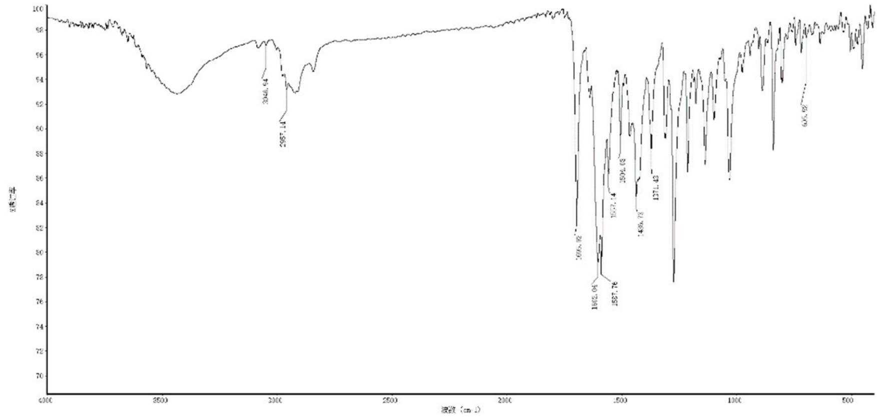

Figure S91 IR spectrum of compound **4v**.

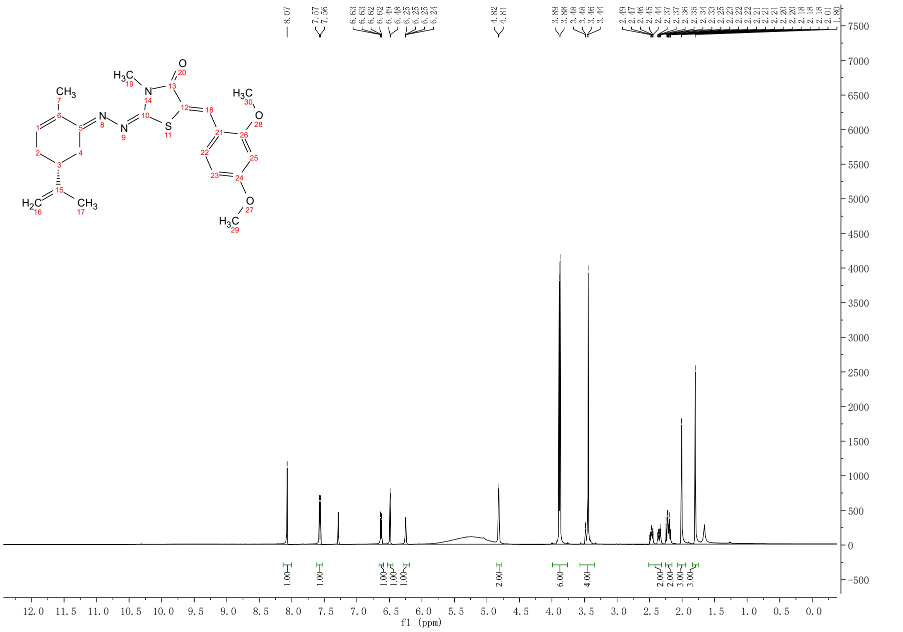

Figure S92 ^{1}H NMR spectrum of compound **4v**.

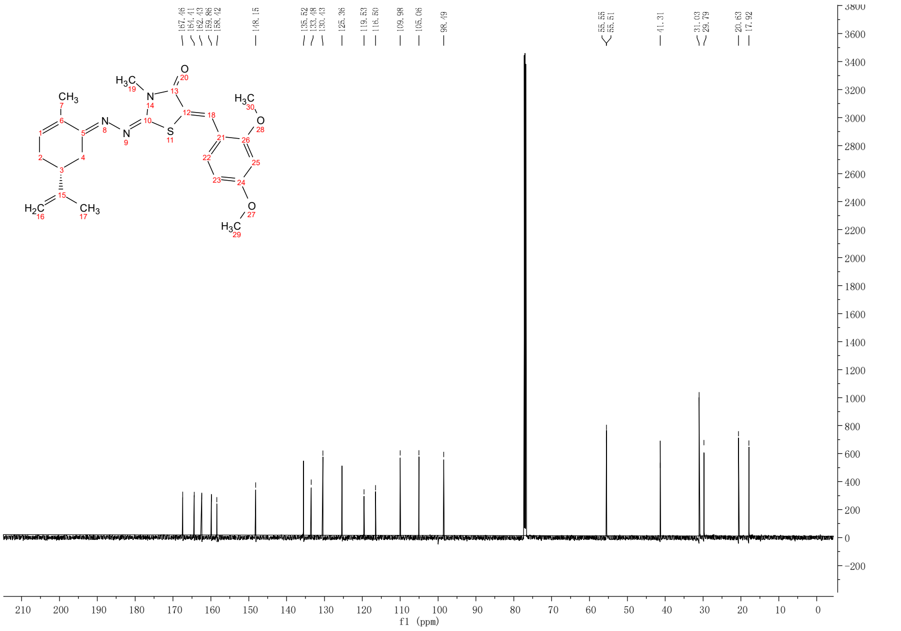

Figure S93 ^{13}C NMR spectrum of compound **4v**.

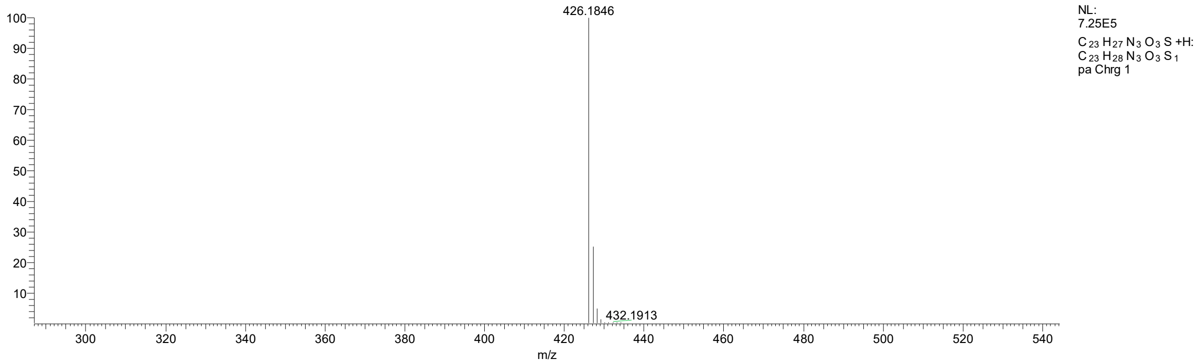

Figure S94 HRMS spectrum of compound **4v**.

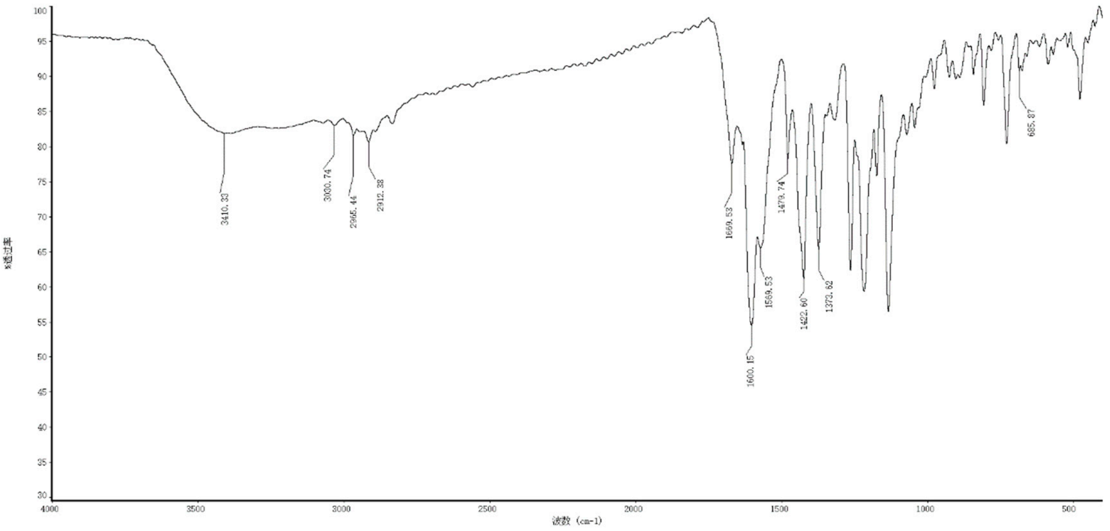

Figure S95 IR spectrum of compound **4w**.

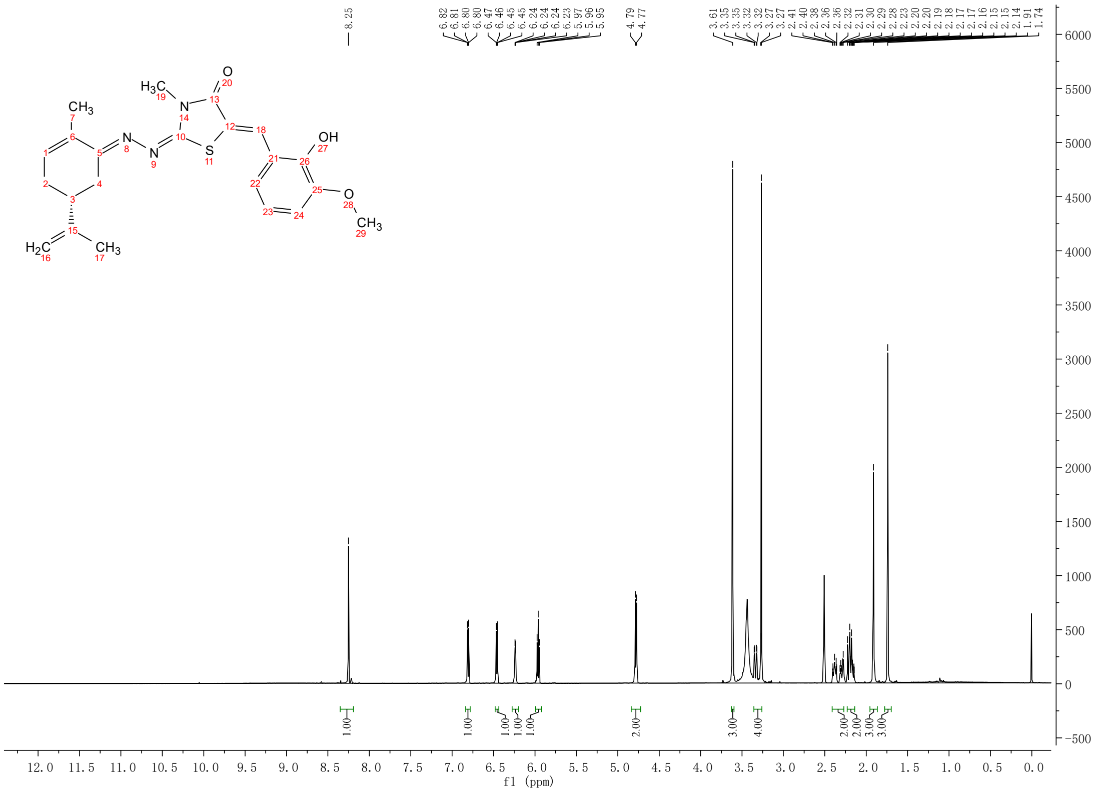

Figure S96 ^{1}H NMR spectrum of compound **4w**.

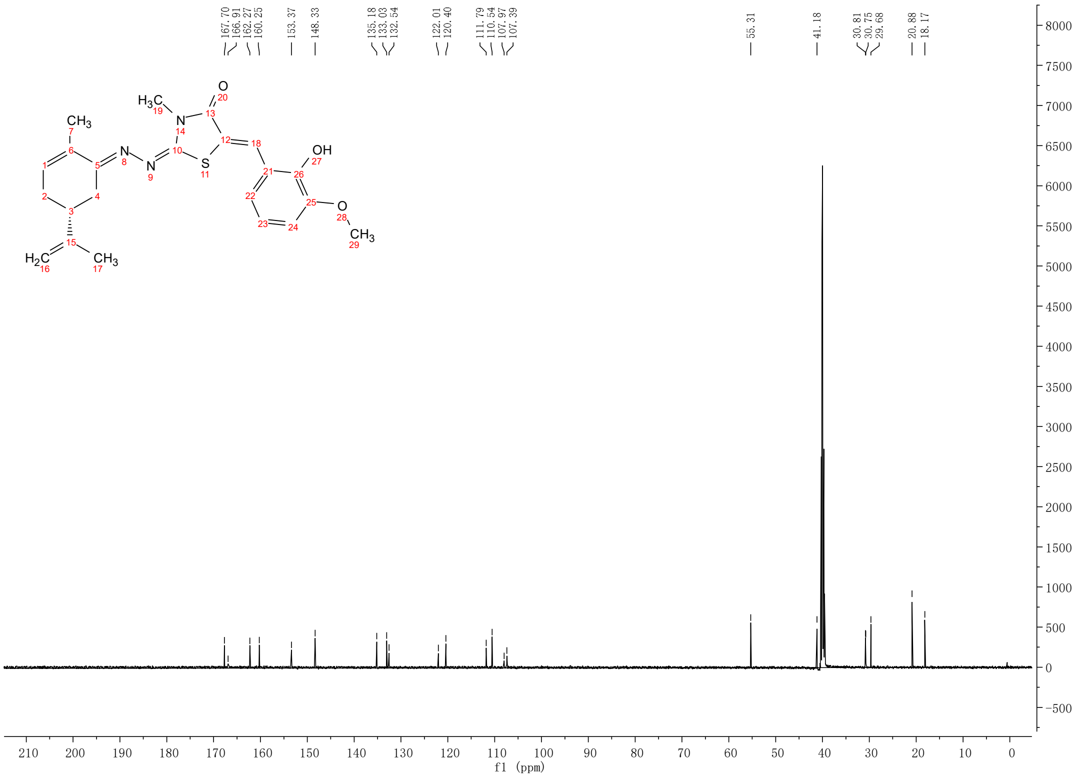

Figure S97 ^{13}C NMR spectrum of compound **4w**.

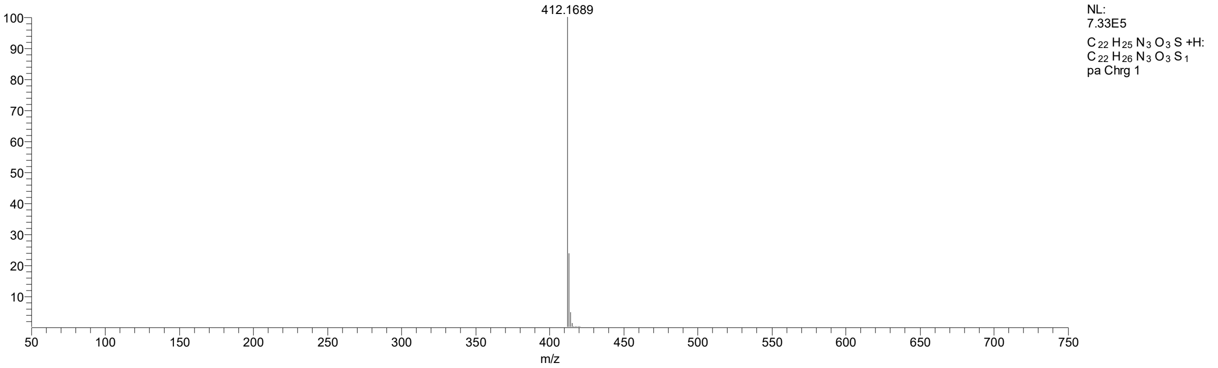

Figure S98 HRMS spectrum of compound **4w**.

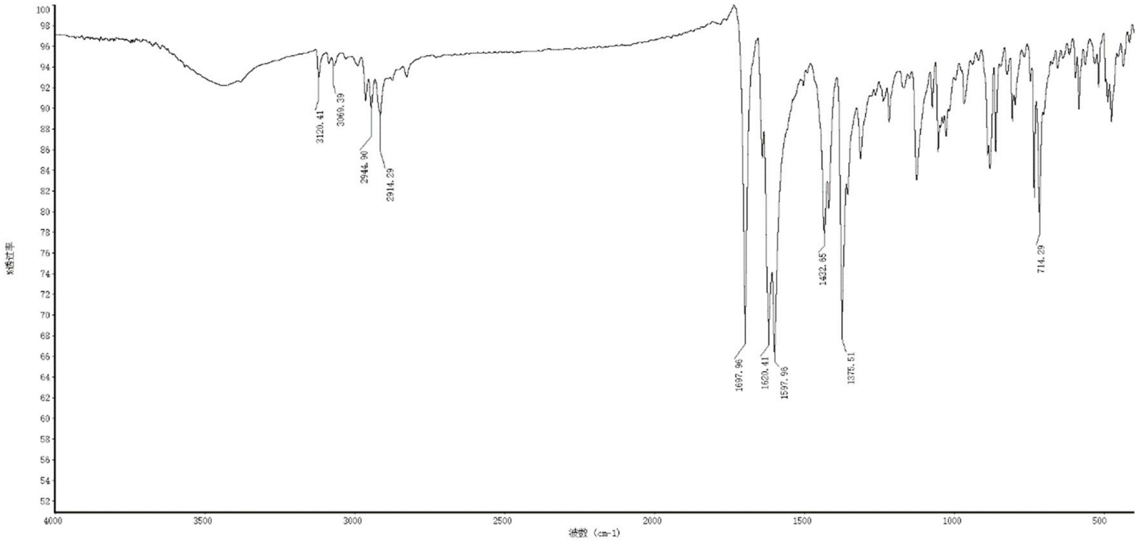

Figure S99 IR spectrum of compound **4x**.

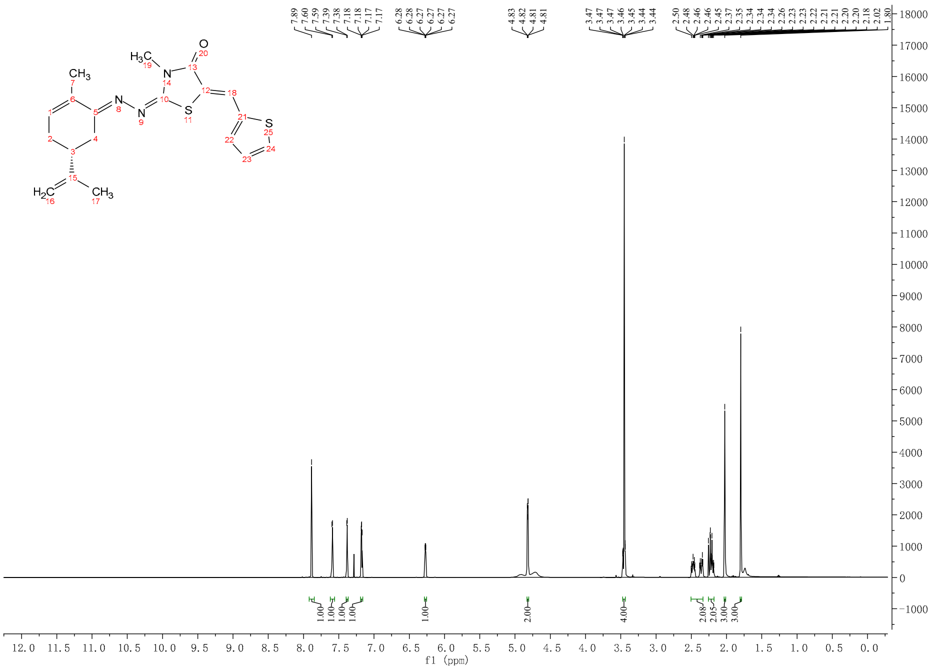

Figure S100 ^{1}H NMR spectrum of compound **4x**.

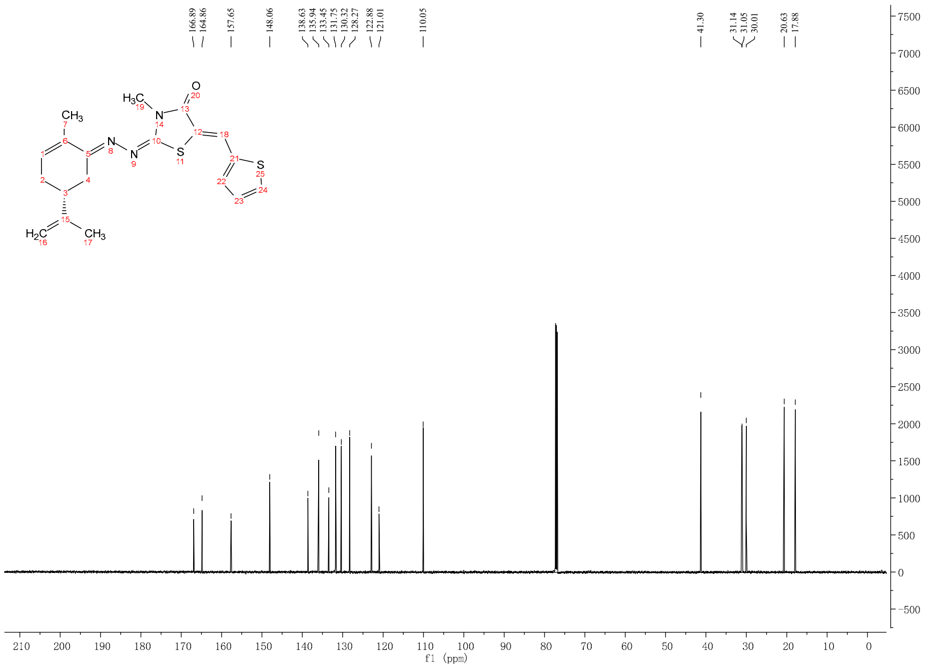

Figure S101 ^{13}C NMR spectrum of compound **4x**.

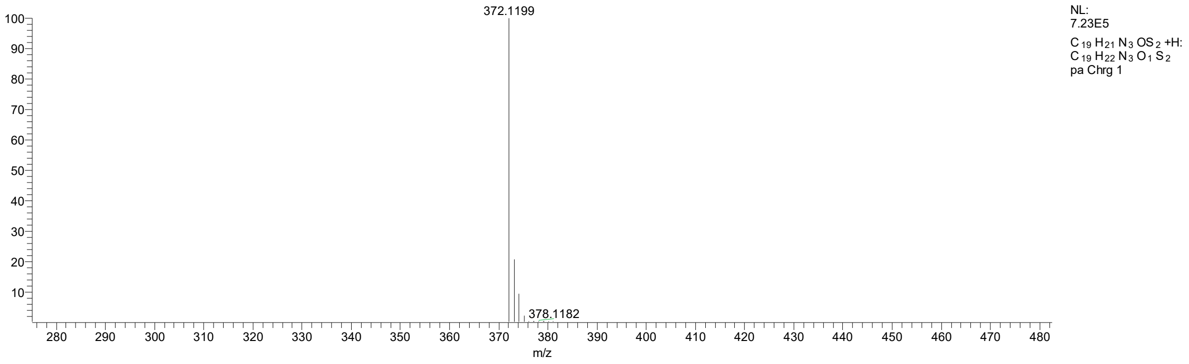

Figure S102 HRMS spectrum of compound **4x**.

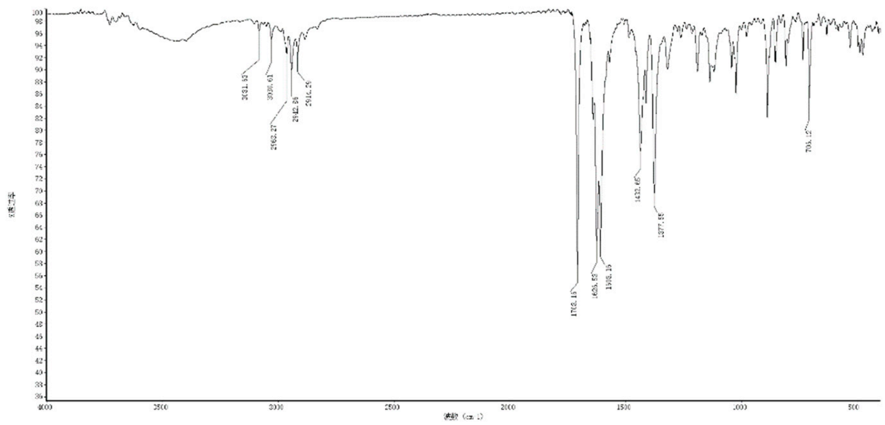

Figure S103 IR spectrum of compound **4y**.

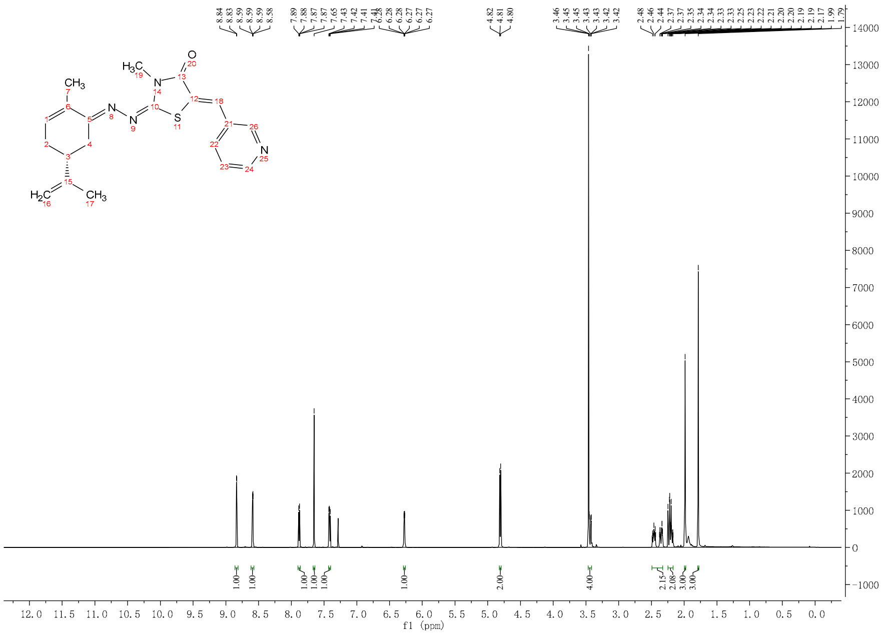

Figure S104 ^{1}H NMR spectrum of compound **4y**.

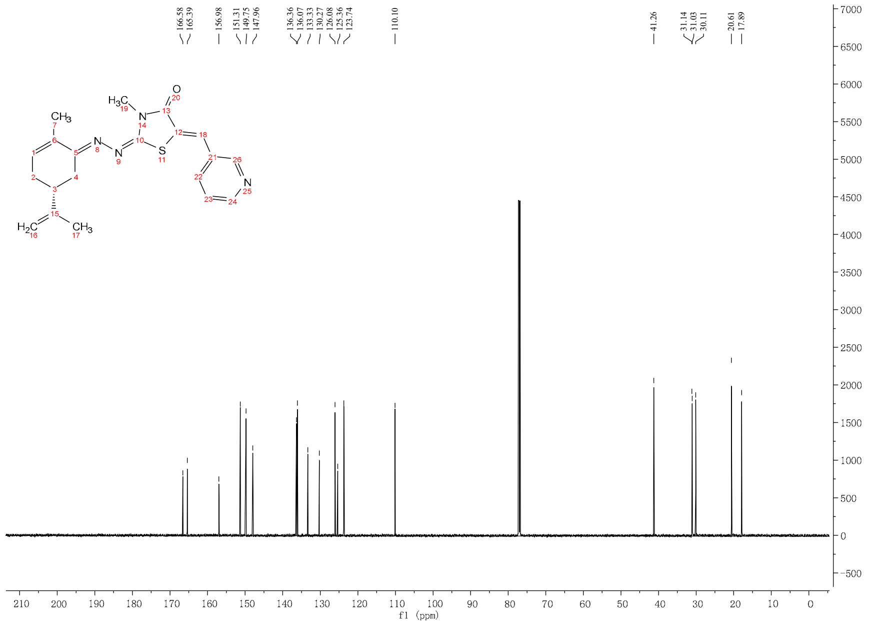

Figure S105 ^{13}C NMR spectrum of compound **4y**.

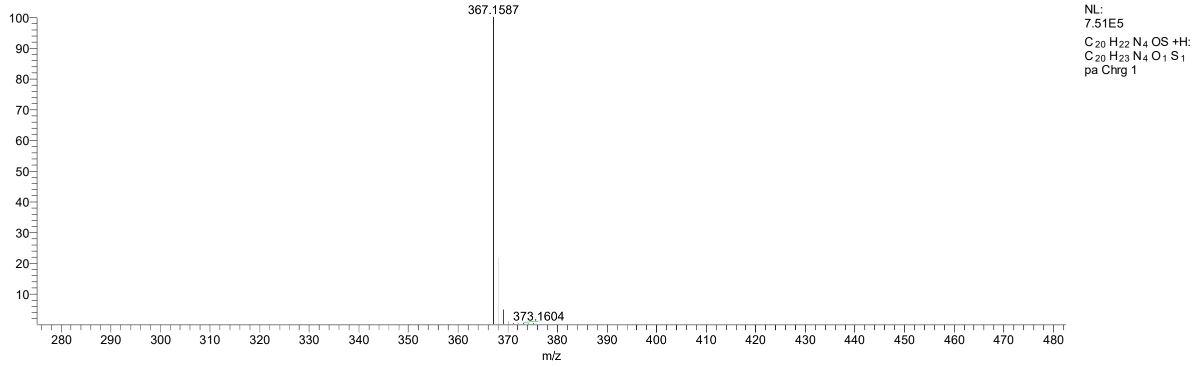

Figure S106 HRMS spectrum of compound **4y**.

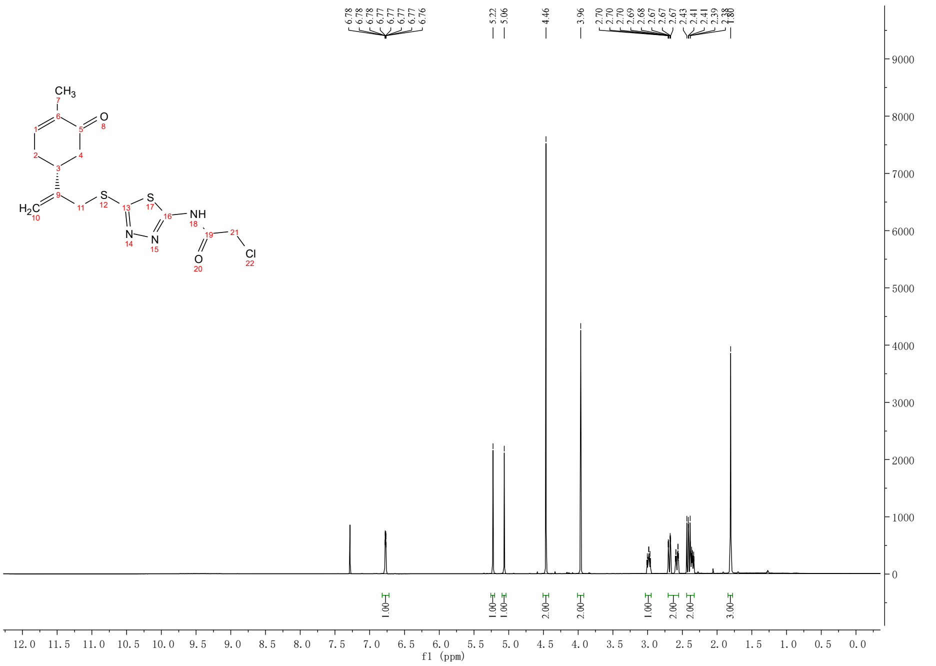

Figure S107 ^{1}H NMR spectrum of compound **6**.

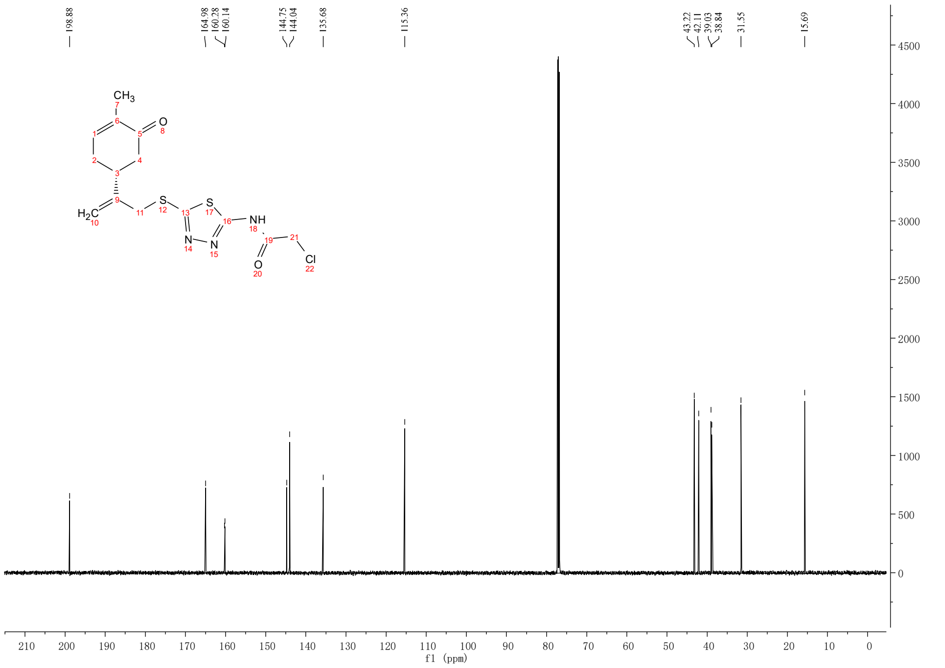

Figure S108 ^{13}C NMR spectrum of compound **6**.

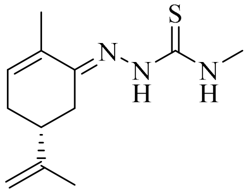

(*R*,*E*)-*N*-methyl-2-(2-methyl-5-(prop-1-en-2-yl)cyclohex-2-en-1-ylidene)hydrazine-1-carbothio-amide (intermediate **2** / *L*-carvone 4-methyl-thiosemicarbazone): white solid; yield, 62.8%; ^{1}H NMR (600 MHz, $CDCl_3$) δ 8.66 (s, 1H, N-H), 7.51 (d, *J* = 2.1 Hz, 1H, N-H), 6.24 – 6.11 (m, 1H, =C-H), 4.81 (dd, *J* = 18.3, 17.1 Hz, 2H, =CH_2), 3.24 (d, *J* = 4.9 Hz, 3H, N-CH_3), 2.69 (dd, *J* = 15.8, 3.9 Hz, 1H, CH), 2.45 – 2.28 (m, 2H, CH_2), 2.15 – 2.06 (m, 2H, CH_2), 1.88 – 1.84 (m, 3H, -CH_3), 1.76 (s, 3H, -CH_3); ^{13}C NMR (151 MHz, $CDCl_3$) δ 178.76, 148.74, 146.92, 134.53, 132.02, 110.71, 40.60, 31.20, 30.05, 29.02, 20.60, 17.80; HRMS (APCI, m/z): calcd. for $C_{12}H_{20}N_3S^+$ ([M+H]$^+$) 238.1378, found 238.1372.

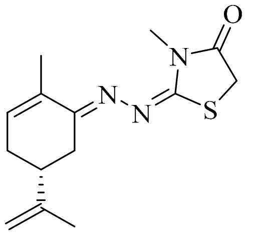

(*E*)-3-methyl-2-(((*R*,*E*)-2-methyl-5-(prop-1-en-2-yl)cyclohex-2-en-1-ylidene)hydrazineylidene) thiazolidin-4-one (intermediate **3**): white solid; yield, 93.0%; ^{1}H NMR (600 MHz, $CDCl_3$) δ 6.26 – 6.18 (m, 1H, =C-H), 4.80 (dd, *J* = 7.3, 0.8 Hz, 2H, =CH_2), 3.74 (s, 2H, O=C-CH_2), 3.42 – 3.38 (m, 1H, CH), 3.30 (s, 3H, N-CH_3), 2.47 – 2.31 (m, 2H, CH_2), 2.21 – 2.14 (m, 2H, CH_2), 1.94 (d, *J* = 1.0 Hz, 3H, -CH_3), 1.78 (s, 3H, -CH_3); ^{13}C NMR (151 MHz, $CDCl_3$) δ 172.22, 164.44, 161.68, 148.12, 135.48, 133.42, 109.97, 41.26, 32.40, 30.99, 30.93, 29.83, 20.62, 17.77; HRMS (APCI, m/z): calcd. for $C_{14}H_{20}N_3OS^+$ ([M+H]$^+$) 278.1327, found 278.1322.

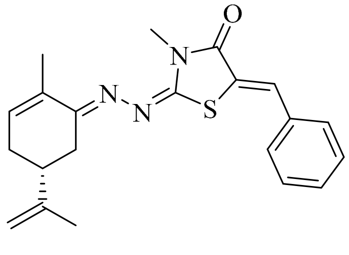

(*E*)-5-((*Z*)-benzylidene)-3-methyl-2-(((*R*,*E*)-2-methyl-5-(prop-1-en-2-yl)cyclohex-2-en-1-ylidene) hydrazineylidene)thiazolidin-4-one (compound **4a**): yellow solid; yield, 91.6%; m.p.: 146.3 – 147.6 °C; IR (KBr, ν/cm^{-1}): 3079.59 (=C-H, Ar-H), 2946.69, 2918.37 (C-H), 1711.85 (C=O), 1597.22 (C=C), 1556.46, 1426.07, 1365.97 (Ar), 689.59 (C-S-C); ^{1}H NMR (600 MHz, $CDCl_3$) δ 7.72 (s, 1H, =C-H), 7.60

(d, J = 7.6 Hz, 2H, Ar-H), 7.48 (t, J = 7.6 Hz, 2H, Ar-H), 7.40 (t, J = 7.3 Hz, 1H, Ar-H), 6.27 (d, J = 4.5 Hz, 1H, =C-H), 4.83 (d, J = 6.8 Hz, 2H, =CH2), 3.51 – 3.41 (m, 4H, N-CH3 & CH), 2.52 – 2.32 (m, 2H, CH2), 2.22 (dt, J = 26.5, 13.4 Hz, 2H, CH2), 2.02 (s, 3H, =C-CH3), 1.80 (s, 3H, =C-CH3); ^{13}C NMR (151 MHz, $CDCl_3$): δ 167.10, 164.93, 157.73, 148.06, 135.94, 134.17, 133.44, 130.21, 130.09, 129.51, 129.00, 122.70, 110.06, 41.30, 31.10, 31.05, 29.95, 20.64, 17.95; HRMS (APCI, m/z): calcd. for $C_{21}H_{24}N_3OS^+$ ($[M+H]^+$) 366.1640, found 366.1635.

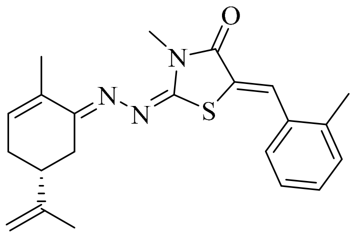

(*E*)-3-methyl-2-(((*R*,*E*)-2-methyl-5-(prop-1-en-2-yl)cyclohex-2-en-1-ylidene)hydrazineylidene)-5-((*Z*)-2-methylbenzylidene)thiazolidin-4-one (compound **4b**): yellow solid; yield, 83.2%; m.p.: 133.3 – 134.5 °C; IR (KBr, ν/cm^{-1}): 3068.90 (=C-H, Ar-H), 2948.07, 2919.64 (C-H), 1704.24 (C=O), 1601.18 (C=C), 1562.09, 1430.60, 1370.19 (Ar), 702.07 (C-S-C); ^{1}H NMR (600 MHz, $CDCl_3$) δ 7.91 (s, 1H, =C-H), 7.62 (d, J = 7.5 Hz, 1H, Ar-H), 7.34 – 7.26 (m, 3H, Ar-H), 6.25 (d, J = 5.6 Hz, 1H, =C-H), 4.82 (d, J = 7.3 Hz, 2H, =CH2), 3.48 – 3.44 (m, 4H, N-CH3 & CH), 2.49 – 2.44 (m, 4H, Ar-CH3 & CH2-a), 2.35 (dt, J = 17.7, 5.0 Hz, 1H, CH2-b), 2.25 – 2.17 (m, 2H, CH2), 1.99 (s, 3H, =C-CH3), 1.80 (s, 3H, =C-CH3); ^{13}C NMR (151 MHz, $CDCl_3$): δ 166.88, 164.81, 157.99, 148.06, 138.54, 135.83, 133.45, 133.22, 130.78, 129.49, 128.13, 127.80, 126.37, 124.02, 110.06, 41.30, 31.08, 31.04, 29.86, 20.64, 20.05, 17.93; HRMS (APCI, m/z): calcd. for $C_{22}H_{26}N_3OS^+$ ($[M+H]^+$) 380.1797, found 380.1791.

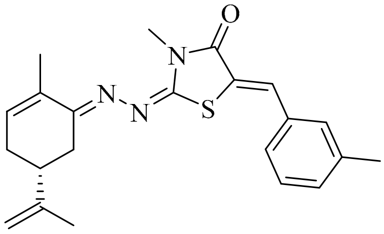

(*E*)-3-methyl-2-(((*R*,*E*)-2-methyl-5-(prop-1-en-2-yl)cyclohex-2-en-1-ylidene)hydrazineylidene)-5-((*Z*)-3-methylbenzylidene)thiazolidin-4-one (compound **4c**): yellow solid; yield, 85.9%; m.p.: 133.2 – 134.4 °C; IR (KBr, ν/cm^{-1}): 3076.01 (=C-H, Ar-H), 2944.52, 2919.64 (C-H), 1700.69 (C=O), 1604.74 (C=C), 1562.09, 1430.60, 1370.19 (Ar), 687.86 (C-S-C); ^{1}H NMR (600 MHz, $CDCl_3$) δ 7.69 (s, 1H, =C-H), 7.43 – 7.36 (m, 3H, Ar-H), 7.22 (d, J = 7.5 Hz, 1H, Ar-H), 6.27 (dd, J = 3.6, 1.4 Hz, 1H, =C-H), 4.82 (d, J = 6.9 Hz, 2H, =CH2), 3.48 – 3.44 (m, 4H, N-CH3 & CH), 2.51 – 2.34 (m, 5H, Ar-CH3 & CH2), 2.26 – 2.18 (m, 2H, CH2), 2.02 (s, 3H, =C-CH3), 1.80 (s, 3H, =C-CH3); ^{13}C NMR (151 MHz, $CDCl_3$): δ 167.17, 164.91, 157.78, 148.07, 138.69, 135.91, 134.12, 133.43, 131.00, 130.50, 130.42, 128.89, 126.97, 122.40,

110.05, 41.30, 31.10, 31.04, 29.92, 21.46, 20.63, 17.90; HRMS (APCI, m/z): calcd. for $C_{22}H_{26}N_3OS^+$ ($[M+H]^+$) 380.1797, found 380.1791.

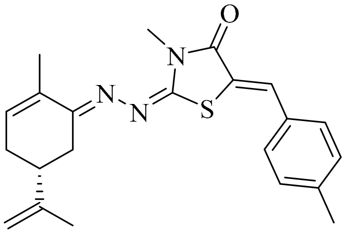

(*E*)-3-methyl-2-(((*R*,*E*)-2-methyl-5-(prop-1-en-2-yl)cyclohex-2-en-1-ylidene)hydrazineylidene)-5-((*Z*)-4-methylbenzylidene)thiazolidin-4-one (compound **4d**): yellow solid; yield, 89.8%; m.p.: 155.3 – 157.0 °C; IR (KBr, ν/cm^{-1}): 3016.33 (=C-H, Ar-H), 2969.39, 2920.41 (C-H), 1716.33 (C=O), 1595.92 (C=C), 1553.06, 1424.49, 1371.43 (Ar), 687.76 (C-S-C); ^{1}H NMR (600 MHz, $CDCl_3$) δ 7.69 (s, 1H, =C-H), 7.49 (d, *J* = 8.1 Hz, 2H, Ar-H), 7.29 (d, *J* = 8.0 Hz, 2H, Ar-H), 6.38 – 6.13 (m, 1H, =C-H), 4.82 (dd, *J* = 5.9, 1.0 Hz, 2H, =CH_2), 3.56 – 3.36 (m, 4H, N-CH_3 & CH), 2.51 – 2.33 (m, 5H, Ar-CH_3 & CH_2), 2.26 – 2.17 (m, 2H, CH_2), 2.02 (s, 3H, =C-CH_3), 1.80 (s, 3H, =C-CH_3); ^{13}C NMR (151 MHz, $CDCl_3$): δ 167.24, 164.78, 157.92, 148.08, 140.00, 135.82, 133.45, 131.41, 130.34, 130.12, 129.75, 121.52, 110.04, 41.30, 31.08, 31.04, 29.90, 21.55, 20.64, 17.94; HRMS (APCI, m/z): calcd. for $C_{22}H_{26}N_3OS^+$ ($[M+H]^+$) 380.1797, found 380.1791.

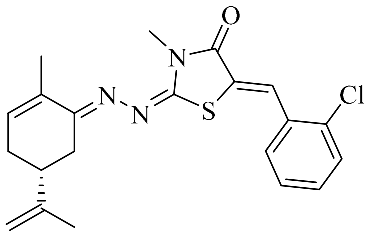

(*E*)-5-((*Z*)-2-chlorobenzylidene)-3-methyl-2-(((*R*,*E*)-2-methyl-5-(prop-1-en-2-yl)cyclohex-2-en-1-ylidene)hydrazineylidene)thiazolidin-4-one (compound **4e**): yellow solid; yield, 78.5%; m.p.: 177.7 – 179.3 °C; IR (KBr, ν/cm^{-1}): 3068.91 (=C-H, Ar-H), 2972.98, 2917.87 (C-H), 1707.52 (C=O), 1599.34 (C=C), 1558.52, 1429.93, 1368.70 (Ar), 678.82 (C-S-C); ^{1}H NMR (600 MHz, $CDCl_3$) δ 8.06 (s, 1H, =C-H), 7.70 (dd, *J* = 7.8, 1.3 Hz, 1H, Ar-H), 7.49 (dd, *J* = 8.0, 1.0 Hz, 1H, Ar-H), 7.41 (dd, *J* = 11.0, 4.1 Hz, 1H, Ar-H), 7.33 (td, *J* = 7.8, 1.5 Hz, 1H, Ar-H), 6.32 – 6.24 (m, 1H, =C-H), 4.85 – 4.80 (m, 2H, =CH_2), 3.49 – 3.43 (m, 4H, N-CH_3 & CH), 2.50 – 2.34 (m, 2H, CH_2), 2.26 – 2.18 (m, 2H, CH_2), 1.98 (s, 3H, =C-CH_3), 1.80 (s, 3H, =C-CH_3); ^{13}C NMR (151 MHz, $CDCl_3$): δ 166.55, 165.17, 157.36, 148.02, 136.13, 135.52, 133.35, 132.60, 130.37, 130.22, 129.12, 127.11, 126.37, 125.67, 110.08, 41.28, 31.13, 31.03, 29.99, 20.62, 17.89; HRMS (APCI, m/z): calcd. for $C_{21}H_{23}ClN_3OS^+$ ($[M+H]^+$) 400.1250, found 400.1245.

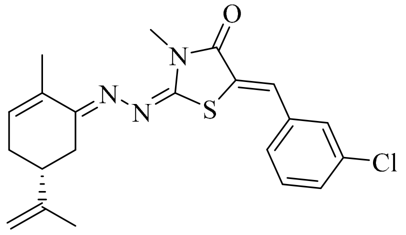

(*E*)-5-((*Z*)-3-chlorobenzylidene)-3-methyl-2-(((*R*,*E*)-2-methyl-5-(prop-1-en-2-yl)cyclohex-2-en-1-ylidene)hydrazineylidene)thiazolidin-4-one (compound **4f**): yellow solid; yield, 80.6%; m.p.: 137.5 – 140.4 °C; IR (KBr, ν/cm^{-1}): 3058.24 (=C-H, Ar-H), 2965.84, 2916.09 (C-H), 1707.80 (C=O), 1597.63 (C=C), 1558.54, 1432.65, 1373.74 (Ar), 679.59 (C-S-C); ^{1}H NMR (600 MHz, $CDCl_3$) δ 7.61 (s, 1H, =C-H), 7.54 (t, *J* = 1.7 Hz, 1H, Ar-H), 7.47 (d, *J* = 7.7 Hz, 1H, Ar-H), 7.41 (t, *J* = 7.8 Hz, 1H, Ar-H), 7.35 (ddd, *J* = 7.9, 1.8, 1.1 Hz, 1H, Ar-H), 6.29 – 6.26 (m, 1H, =C-H), 4.82 (dd, *J* = 7.6, 0.9 Hz, 2H, =CH_2), 3.47 – 3.43 (m, 4H, N-CH_3 & CH), 2.50 – 2.34 (m, 2H, CH_2), 2.25 – 2.18 (m, 2H, CH_2), 2.01 (s, 3H, =C-CH_3), 1.80 (s, 3H, =C-CH_3); ^{13}C NMR (151 MHz, $CDCl_3$): δ 166.75, 165.24, 157.18, 148.00, 136.20, 135.96, 134.98, 133.39, 130.18, 129.93, 129.36, 128.40, 127.70, 124.41, 110.09, 41.28, 31.14, 31.05, 30.03, 20.63, 17.91; HRMS (APCI, m/z): calcd. for $C_{21}H_{23}ClN_3OS^+$ ($[M+H]^+$) 400.1250, found 400.1245.

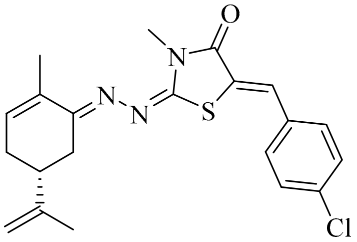

(*E*)-5-((*Z*)-4-chlorobenzylidene)-3-methyl-2-(((*R*,*E*)-2-methyl-5-(prop-1-en-2-yl)cyclohex-2-en-1-ylidene)hydrazineylidene)thiazolidin-4-one (compound **4g**): yellow solid; yield, 89.3%; m.p.: 178.4 – 179.4 °C; IR (KBr, ν/cm^{-1}): 3083.67 (=C-H, Ar-H), 2963.27, 2918.37 (C-H), 1708.16 (C=O), 1600.00 (C=C), 1559.18, 1426.53, 1371.43 (Ar), 706.12 (C-S-C); ^{1}H NMR (600 MHz, $CDCl_3$) δ 7.65 (s, 1H, =C-H), 7.52 (d, *J* = 8.5 Hz, 2H, Ar-H), 7.49 – 7.41 (m, 2H, Ar-H), 6.31 – 6.26 (m, 1H, =C-H), 4.83 – 4.81 (m, 2H, =CH_2), 3.49 – 3.42 (m, 4H, N-CH_3 & CH), 2.51 – 2.34 (m, 2H, CH_2), 2.26 – 2.18 (m, 2H, CH_2), 2.01 (s, 3H, =C-CH_3), 1.80 (s, 3H, =C-CH_3); ^{13}C NMR (151 MHz, $CDCl_3$): δ 166.94, 165.21, 157.29, 148.02, 136.18, 135.43, 133.36, 132.66, 131.15, 129.29, 128.73, 123.39, 110.07, 41.28, 31.13, 31.04, 30.02, 20.62, 17.91; HRMS (APCI, m/z): calcd. for $C_{21}H_{23}ClN_3OS^+$ ($[M+H]^+$) 400.1250, found 400.1245.

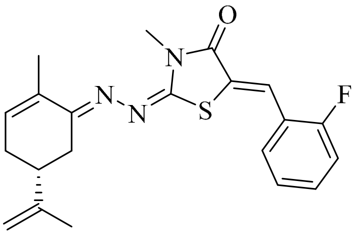

(*E*)-5-((*Z*)-2-fluorobenzylidene)-3-methyl-2-(((*R*,*E*)-2-methyl-5-(prop-1-en-2-yl)cyclohex-2-en-1-ylidene)hydrazineylidene)thiazolidin-4-one (compound **4h**): yellow solid; yield, 82.0%; m.p.: 133.4 – 135.8 °C; IR (KBr, ν/cm^{-1}): 3085.61 (=C-H, Ar-H), 2967.07, 2939.98 (C-H), 1703.86 (C=O), 1625.96 (C=C), 1487.11, 1432.93, 1378.74 (Ar), 684.48 (C-S-C); ^{1}H NMR (600 MHz, $CDCl_3$) δ 7.95 (s, 1H, =C-H), 7.68 (td, *J* = 7.7, 1.4 Hz, 1H, Ar-H), 7.41 – 7.37 (m, 1H, Ar-H), 7.29 (t, *J* = 7.6 Hz, 1H, Ar-H), 7.20 – 7.11 (m, 1H, Ar-H), 6.32 – 6.22 (m, 1H, =C-H), 4.88 – 4.77 (m, 2H, =CH_2), 3.53 – 3.38 (m, 4H, N-CH_3 & CH), 2.51 – 2.34 (m, 2H, CH_2), 2.26 – 2.18 (m, 2H, CH_2), 2.00 (s, 3H, =C-CH_3), 1.80 (s, 3H, =C-CH_3); ^{13}C NMR (151 MHz, $CDCl_3$): δ 166.68, 165.12, 161.23 (d, *J* = 254.2 Hz), 157.38, 148.02, 136.09, 133.37, 131.20 (d, *J* = 8.6 Hz), 129.05, 129.04, 124.73, 124.48 (d, *J* = 3.7 Hz), 122.54 (d, *J* = 12.0 Hz), 121.87, 121.82, 115.94 (d, *J* = 21.7 Hz), 110.07, 41.28, 31.12, 31.04, 30.00, 20.62, 17.91; HRMS (APCI, m/z): calcd. for $C_{21}H_{23}FN_3OS^+$ ($[M+H]^+$) 384.1546, found 384.1540.

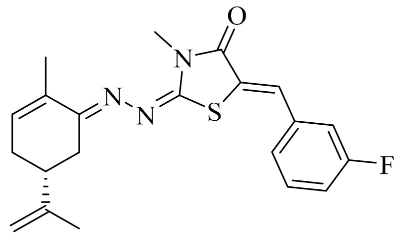

(*E*)-5-((*Z*)-3-fluorobenzylidene)-3-methyl-2-(((*R*,*E*)-2-methyl-5-(prop-1-en-2-yl)cyclohex-2-en-1-ylidene)hydrazineylidene)thiazolidin-4-one (compound **4i**): yellow solid; yield, 90.3%; m.p.: 144.4 – 146.4 °C; IR (KBr, ν/cm^{-1}): 3081.63 (=C-H, Ar-H), 2965.31, 2930.61 (C-H), 1708.16 (C=O), 1606.12 (C=C), 1583.67, 1493.88, 1432.65, 1373.47 (Ar), 681.63 (C-S-C); ^{1}H NMR (600 MHz, $CDCl_3$) δ 7.66 (s, 1H, =C-H), 7.45 (td, *J* = 8.0, 6.0 Hz, 1H, Ar-H), 7.37 (d, *J* = 7.8 Hz, 1H, Ar-H), 7.30 (s, 1H, Ar-H), 7.10 (td, *J* = 8.3, 2.1 Hz, 1H, Ar-H), 6.31 – 6.26 (m, 1H, =C-H), 4.82 (d, *J* = 8.0 Hz, 2H, =CH_2), 3.47 – 3.43 (m, 4H, N-CH_3 & CH), 2.51 – 2.34 (m, 2H, CH_2), 2.26 – 2.19 (m, 2H, CH_2), 2.02 (s, 3H, =C-CH_3), 1.80 (s, 3H, =C-CH_3); ^{13}C NMR (151 MHz, $CDCl_3$): δ 166.84, 165.28, 162.90 (d, *J* = 247.0 Hz), 157.20, 148.02, 136.29 (d, *J* = 7.9 Hz), 136.20, 133.39, 130.52 (d, *J* = 8.3 Hz), 128.68 (d, *J* = 2.7 Hz), 125.80 (d, *J* = 2.8 Hz), 124.31, 116.47, 116.33 (d, *J* = 1.7 Hz), 110.08, 41.29, 31.14, 31.04, 30.03, 20.62, 17.93; HRMS (APCI, m/z): calcd. for $C_{21}H_{23}FN_3OS^+$ ($[M+H]^+$) 384.1546, found 384.1540.

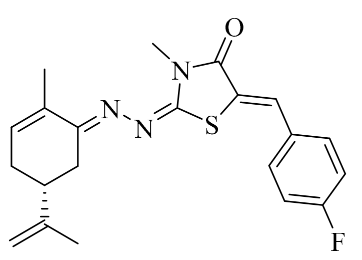

(*E*)-5-((*Z*)-4-fluorobenzylidene)-3-methyl-2-(((*R*,*E*)-2-methyl-5-(prop-1-en-2-yl)cyclohex-2-en-1-ylidene)hydrazineylidene)thiazolidin-4-one (compound **4j**): yellow solid; yield, 87.7%; m.p.: 187.4 –

188.6 ℃; IR (KBr, ν/cm^{-1}): 3083.67 (=C-H, Ar-H), 2938.78, 2912.24 (C-H), 1704.08 (C=O), 1610.20 (C=C), 1510.20, 1436.73, 1375.51 (Ar), 689.80 (C-S-C); ^{1}H NMR (600 MHz, $CDCl_3$) δ 7.67 (s, 1H, =C-H), 7.58 (dd, J = 8.7, 5.4 Hz, 2H, Ar-H), 7.18 (t, J = 8.6 Hz, 2H, Ar-H), 6.29 – 6.26 (m, 1H, =C-H), 4.83 – 4.81 (m, 2H, =CH_2), 3.48 – 3.44 (m, 4H, N-CH_3 & CH), 2.51 – 2.34 (m, 2H, CH_2), 2.26 – 2.18 (m, 2H, CH_2), 2.02 (s, 3H, =C-CH_3), 1.80 (s, 3H, =C-CH_3); ^{13}C NMR (151 MHz, $CDCl_3$): δ 167.03, 165.09, 163.05 (d, J = 252.1 Hz), 157.45, 148.04, 136.09, 133.37, 131.99 (d, J = 8.6 Hz), 130.47 (d, J = 3.4 Hz), 128.95, 122.38, 122.37, 116.24 (d, J = 22.1 Hz), 110.06, 41.29, 31.13, 31.04, 29.98, 20.62, 17.92; HRMS (APCI, m/z): calcd. for $C_{21}H_{23}FN_3OS^+$ ($[M+H]^+$) 384.1546, found 384.1540.

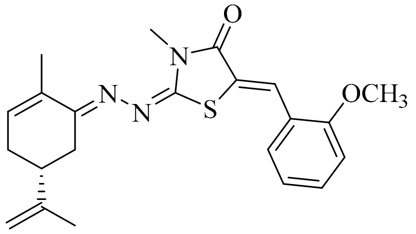

(*E*)-5-((*Z*)-2-methoxybenzylidene)-3-methyl-2-(((*R*,*E*)-2-methyl-5-(prop-1-en-2-yl)cyclohex-2-en-1-ylidene)hydrazineylidene)thiazolidin-4-one (compound **4k**): yellow solid; yield, 82.1%; m.p.: 207.7 – 208.4 ℃; IR (KBr, ν/cm^{-1}): 3071.43 (=C-H, Ar-H), 2971.43, 2918.37 (C-H), 1704.08 (C=O), 1612.24 (C=C), 1593.88, 1428.57, 1371.43 (Ar), 687.76 (C-S-C); ^{1}H NMR (600 MHz, $CDCl_3$) δ 8.12 (s, 1H, =C-H), 7.62 (dd, J = 7.7, 1.5 Hz, 1H, Ar-H), 7.38 (td, J = 8.5, 1.6 Hz, 1H, Ar-H), 7.08 (t, J = 7.4 Hz, 1H, Ar-H), 6.95 (d, J = 8.0 Hz, 1H, Ar-H), 6.27 – 6.23 (m, 1H, =C-H), 4.83 – 4.81 (m, 2H, =CH_2), 3.91 (s, 3H, -OCH_3), 3.48 – 3.44 (m, 4H, N-CH_3 & CH), 2.50 – 2.33 (m, 2H, CH_2), 2.25 – 2.17 (m, 2H, CH_2), 2.00 (s, 3H, =C-CH_3), 1.80 (s, 3H, =C-CH_3); ^{13}C NMR (151 MHz, $CDCl_3$): δ 167.20, 164.67, 158.27, 158.14, 148.11, 135.72, 133.46, 131.14, 129.13, 125.52, 123.36, 122.51, 120.70, 110.92, 110.01, 55.52, 41.30, 31.06, 31.03, 29.84, 20.63, 17.91; HRMS (APCI, m/z): calcd. for $C_{22}H_{26}N_3O_2S^+$ ($[M+H]^+$) 396.1746, found 396.1740.

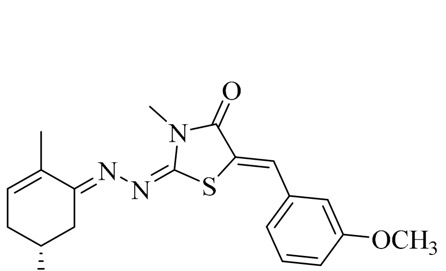

(*E*)-5-((*Z*)-3-methoxybenzylidene)-3-methyl-2-(((*R*,*E*)-2-methyl-5-(prop-1-en-2-yl)cyclohex-2-en-1-ylidene)hydrazineylidene)thiazolidin-4-one (compound **4l**): yellow solid; yield, 90.5%; m.p.: 91.1 – 92.0 ℃; IR (KBr, ν/cm^{-1}): 3004.08 (=C-H, Ar-H), 2963.27, 2938.78 (C-H), 1704.08 (C=O), 1600.00 (C=C), 1500.00, 1434.69, 1375.51 (Ar), 683.67 (C-S-C); ^{1}H NMR (600 MHz, $CDCl_3$) δ 7.68 (s, 1H, =C-H), 7.39 (t, J = 8.0 Hz, 1H, Ar-H), 7.20 (d, J = 7.7 Hz, 1H, Ar-H), 7.14 – 7.08 (m, 1H, Ar-H), 6.99 – 6.92 (m, 1H,

Ar-H), 6.29 – 6.24 (m, 1H, =C-H), 4.83 – 4.81 (m, 2H, =CH_2), 3.87 (s, 3H, -OCH_3), 3.48 – 3.42 (m, 4H, N-CH_3 & CH), 2.51 – 2.33 (m, 2H, CH_2), 2.25 – 2.18 (m, 2H, CH_2), 2.00 (s, 3H, =C-CH_3), 1.80 (s, 3H, =C-CH_3); ^{13}C NMR (151 MHz, $CDCl_3$): δ 167.07, 164.93, 159.87, 157.75, 148.06, 135.98, 135.48, 133.41, 130.17, 129.99, 123.07, 122.51, 115.64, 114.96, 110.05, 55.29, 41.29, 31.09, 31.04, 29.93, 20.63, 17.86; HRMS (APCI, m/z): calcd. for $C_{22}H_{26}N_3O_2S^+$ ([M+H]$^+$) 396.1746, found 396.1740.

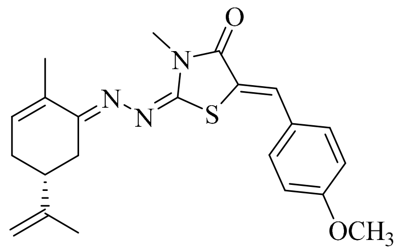

(*E*)-5-((*Z*)-4-methoxybenzylidene)-3-methyl-2-(((*R*,*E*)-2-methyl-5-(prop-1-en-2-yl)cyclohex-2-en-1-ylidene)hydrazineylidene)thiazolidin-4-one (compound **4m**): yellow solid; yield, 91.2%; m.p.: 148.5 – 150.6 °C; IR (KBr, ν/cm^{-1}): 3071.43, 3012.24 (=C-H, Ar-H), 2916.33 (C-H), 1712.24 (C=O), 1600.00 (C=C), 1514.29, 1428.57, 1375.51 (Ar), 687.76 (C-S-C); ^{1}H NMR (600 MHz, $CDCl_3$) δ 7.67 (s, 1H, =C-H), 7.55 (d, *J* = 8.7 Hz, 2H, Ar-H), 7.01 (d, *J* = 8.8 Hz, 2H, Ar-H), 6.33 – 6.19 (m, 1H, =C-H), 4.82 (d, *J* = 6.1 Hz, 2H, =CH_2), 3.88 (s, 3H, -OCH_3), 3.55 – 3.36 (m, 4H, N-CH_3 & CH), 2.50 – 2.33 (m, 2H, CH_2), 2.26 – 2.18 (m, 2H, CH_2), 2.02 (s, 3H, =C-CH_3), 1.80 (s, 3H, =C-CH_3); ^{13}C NMR (151 MHz, $CDCl_3$): δ 167.36, 164.70, 160.65, 157.99, 148.10, 135.76, 133.45, 131.93, 130.14, 126.88, 119.84, 114.54, 110.03, 55.43, 41.31, 31.09, 31.04, 29.89, 20.64, 17.95; HRMS (APCI, m/z): calcd. for $C_{22}H_{26}N_3O_2S^+$ ([M+H]$^+$) 396.1746, found 396.1740.

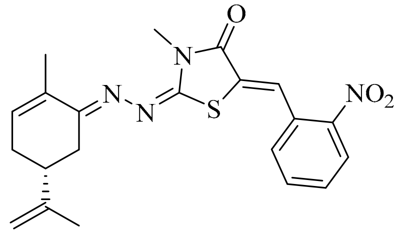

(*E*)-3-methyl-2-(((*R*,*E*)-2-methyl-5-(prop-1-en-2-yl)cyclohex-2-en-1-ylidene)hydrazineylidene)-5-((*Z*)-2-nitrobenzylidene)thiazolidin-4-one (compound **4n**): yellow solid; yield, 88.3%; m.p.: 152.7 – 154.7 °C; IR (KBr, ν/cm^{-1}): 3069.39 (=C-H, Ar-H), 2963.27, 2920.41 (C-H), 1708.16 (C=O), 1608.16 (C=C), 1530.61, 1430.61, 1373.47 (Ar), 700.00 (C-S-C); ^{1}H NMR (600 MHz, $CDCl_3$) δ 8.13 (dd, *J* = 8.2, 0.8 Hz, 1H, Ar-H), 8.03 (s, 1H, =C-H), 7.75 (ddd, *J* = 15.0, 7.8, 3.6 Hz, 2H, Ar-H), 7.59 – 7.53 (m, 1H, Ar-H), 6.29 – 6.20 (m, 1H, =C-H), 4.80 (d, *J* = 10.8 Hz, 2H, =CH_2), 3.51 – 3.39 (m, 4H, N-CH_3 & CH), 2.48 – 2.31 (m, 2H, CH_2), 2.24 – 2.15 (m, 2H, CH_2), 1.92 (s, 3H, =C-CH_3), 1.78 (s, 3H, =C-CH_3); ^{13}C NMR (151 MHz, $CDCl_3$): δ 165.83, 165.34, 156.95, 148.08, 147.92, 136.35, 133.71, 133.23, 130.18, 129.81,

129.73, 127.99, 125.76, 125.34, 110.11, 41.24, 31.14, 31.01, 30.05, 20.61, 17.82; HRMS (APCI, m/z): calcd. for $C_{21}H_{23}N_4O_3S^+$ ($[M+H]^+$) 411.1491, found 411.1485.

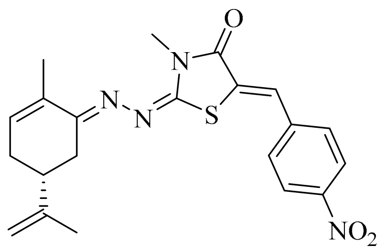

(*E*)-3-methyl-2-(((*R*,*E*)-2-methyl-5-(prop-1-en-2-yl)cyclohex-2-en-1-ylidene)hydrazineylidene)-5-((*Z*)-4-nitrobenzylidene)thiazolidin-4-one (compound **4o**): yellow solid; yield, 78.2%; m.p.: 227.8 – 228.1 °C; IR (KBr, ν/cm^{-1}): 3081.63 (=C-H, Ar-H), 2924.49 (C-H), 1708.16 (C=O), 1608.16 (C=C), 1512.24, 1432.65, 1375.51 (Ar), 685.71 (C-S-C); ^{1}H NMR (600 MHz, $CDCl_3$) δ 8.34 (d, *J* = 8.7 Hz, 2H, Ar-H), 7.84 – 7.60 (m, 3H, =C-H & Ar-H), 6.32 (dd, *J* = 3.5, 1.4 Hz, 1H, =C-H), 4.83 (d, *J* = 10.5 Hz, 2H, =CH_2), 3.65 – 3.34 (m, 4H, N-CH_3 & CH), 2.51 – 2.36 (m, 2H, CH_2), 2.28 – 2.19 (m, 2H, CH_2), 2.02 (s, 3H, =C-CH_3), 1.80 (s, 3H, =C-CH_3); ^{13}C NMR (151 MHz, $CDCl_3$): δ 166.44, 165.83, 156.51, 147.92, 147.37, 140.43, 136.73, 133.26, 130.40, 127.54, 126.91, 124.23, 110.15, 41.27, 31.21, 31.05, 30.22, 20.61, 17.90; HRMS (APCI, m/z): calcd. for $C_{21}H_{23}N_4O_3S^+$ ($[M+H]^+$) 411.1491, found 411.1485.

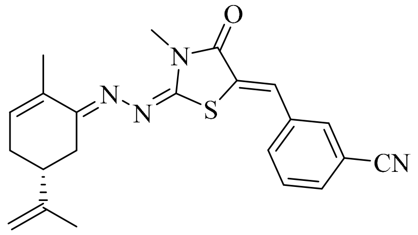

3-((*Z*)-((*E*)-3-methyl-2-(((*R*,*E*)-2-methyl-5-(prop-1-en-2-yl)cyclohex-2-en-1-ylidene)hydrazine-ylidene)-4-oxothiazolidin-5-ylidene)methyl)benzonitrile (compound **4p**): yellow solid; yield, 79.8%; m.p.: 158.6 – 161.7 °C; IR (KBr, ν/cm^{-1}): 3069.39 (=C-H, Ar-H), 2924.49 (C-H), 2230.61 (C≡N), 1712.24 (C=O), 1604.08 (C=C), 1428.57, 1371.43 (Ar), 683.67 (C-S-C); ^{1}H NMR (600 MHz, $CDCl_3$) δ 7.86 – 7.74 (m, 2H, Ar-H), 7.68 – 7.56 (m, 3H, =C-H & Ar-H), 6.29 (d, *J* = 1.4 Hz, 1H, =C-H), 4.83 – 4.80 (m, 2H, =CH_2), 3.48 – 3.41 (m, 4H, N-CH_3 & CH), 2.44 (ddd, *J* = 59.8, 35.6, 10.8 Hz, 2H, CH_2), 2.26 – 2.17 (m, 2H, CH_2), 2.00 (s, 3H, =C-CH_3), 1.79 (s, 3H, =C-CH_3); ^{13}C NMR (151 MHz, $CDCl_3$): δ 166.46, 165.64, 156.52, 147.94, 136.53, 135.50, 133.57, 133.32, 133.01, 132.28, 129.88, 127.04, 127.03, 125.84, 118.16, 113.45, 110.12, 41.26, 31.19, 31.04, 30.17, 20.62, 17.98; HRMS (APCI, m/z): calcd. for $C_{22}H_{23}N_4OS^+$ ($[M+H]^+$) 391.1593, found 391.1587.

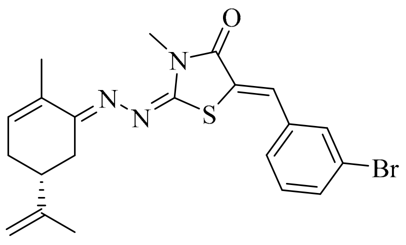

(*E*)-5-((*Z*)-3-bromobenzylidene)-3-methyl-2-(((*R*,*E*)-2-methyl-5-(prop-1-en-2-yl)cyclohex-2-en-1-ylidene)hydrazineylidene)thiazolidin-4-one (compound **4q**): yellow solid; yield, 77.6%; m.p.: 124.9 – 125.7 °C; IR (KBr, ν/cm^{-1}): 3068.28 (=C-H, Ar-H), 2970.39, 2916.01 (C-H), 1712.39 (C=O), 1600.00 (C=C), 1556.50, 1433.23, 1375.23 (Ar), 683.90 (C-S-C); ^{1}H NMR (600 MHz, $CDCl_3$) δ 7.69 (s, 1H, =C-H), 7.60 (s, 1H, Ar-H), 7.51 (t, *J* = 6.4 Hz, 2H, Ar-H), 7.35 (t, *J* = 7.9 Hz, 1H, Ar-H), 6.28 (d, *J* = 4.7 Hz, 1H, =C-H), 4.82 (d, *J* = 8.3 Hz, 2H, =CH_2), 3.47 – 3.42 (m, 4H, N-CH_3 & CH), 2.50 – 2.34 (m, 2H, CH_2), 2.25 – 2.18 (m, 2H, CH_2), 2.01 (s, 3H, =C-CH_3), 1.80 (s, 3H, =C-CH_3); ^{13}C NMR (151 MHz, $CDCl_3$): δ 166.74, 165.26, 157.17, 148.00, 136.24, 136.22, 133.38, 132.92, 132.26, 130.42, 128.30, 128.04, 124.43, 123.08, 110.09, 41.28, 31.14, 31.04, 30.04, 20.63, 17.90; HRMS (APCI, m/z): calcd. for $C_{21}H_{23}BrN_3OS^+$ ($[M+H]^+$) 444.0745, found 444.0740.

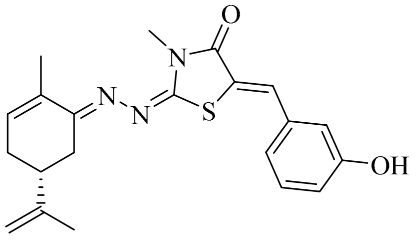

(*E*)-5-((*Z*)-3-hydroxybenzylidene)-3-methyl-2-(((*R*,*E*)-2-methyl-5-(prop-1-en-2-yl)cyclohex-2-en-1-ylidene)hydrazineylidene)thiazolidin-4-one (compound **4r**): yellow solid; yield, 90.4%; m.p.: 198.9 – 205.0 °C; IR (KBr, ν/cm^{-1}): 3348.98 (O-H), 3079.59 (=C-H, Ar-H), 2924.49 (C-H), 1701.20 (C=O), 1598.69 (C=C), 1563.20, 1437.02, 1371.43 (Ar), 679.59 (C-S-C); ^{1}H NMR (600 MHz, DMSO-d_6) δ 9.82 (s, 1H, -OH), 7.52 (s, 1H, =C-H), 7.30 (t, *J* = 7.9 Hz, 1H, Ar-H), 7.06 – 6.98 (m, 2H, Ar-H), 6.85 (dd, *J* = 8.1, 1.8 Hz, 1H, Ar-H), 6.26 (dd, *J* = 3.1, 1.4 Hz, 1H, =C-H), 4.76 (s, 2H, =CH_2), 3.32 – 3.25 (m, 4H, N-CH_3 & CH), 2.38 – 2.24 (m, 2H, CH_2), 2.20 – 2.09 (m, 2H, CH_2), 1.89 (s, 3H, =C-CH_3), 1.71 (s, 3H, =C-CH_3); ^{13}C NMR (151 MHz, DMSO-d_6): δ 166.44, 164.45, 158.25, 158.04, 148.11, 136.70, 135.28, 132.74, 130.63, 130.09, 122.45, 121.56, 117.55, 116.34, 110.58, 41.06, 30.94, 30.76, 30.17, 20.79, 18.06; HRMS (APCI, m/z): calcd. for $C_{21}H_{24}N_3O_2S^+$ ($[M+H]^+$) 382.1589, found 382.1584.

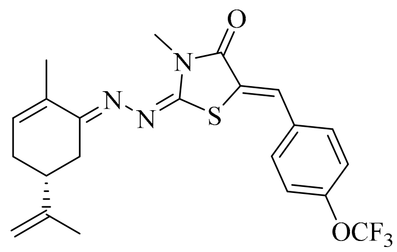

(E)-3-methyl-2-(((R,E)-2-methyl-5-(prop-1-en-2-yl)cyclohex-2-en-1-ylidene)hydrazineylidene)-5-((Z)-4-(trifluoromethoxy)benzylidene)thiazolidin-4-one (compound **4s**): yellow solid; yield, 77.4%; m.p.: 153.2 – 153.8 °C; IR (KBr, ν/cm^{-1}): 3077.33 (=C-H, Ar-H), 2923.55 (C-H), 1713.03 (C=O), 1602.63 (C=C), 1508.16, 1428.57, 1371.43 (Ar), 693.88 (C-S-C); ^{1}H NMR (600 MHz, $CDCl_3$) δ 7.68 (s, 1H, =C-H), 7.65 – 7.61 (m, 2H, Ar-H), 7.33 (d, J = 8.2 Hz, 2H, Ar-H), 6.31 – 6.27 (m, 1H, =C-H), 4.83 (dd, J = 5.6, 4.2 Hz, 2H, =CH_2), 3.48 – 3.44 (m, 4H, N-CH_3 & CH), 2.51 – 2.35 (m, 2H, CH_2), 2.27 – 2.18 (m, 2H, CH_2), 2.02 (s, 3H, =C-CH_3), 1.80 (s, 3H, =C-CH_3); ^{13}C NMR (151 MHz, $CDCl_3$): δ 13C NMR (151 MHz, CDCl3) δ 166.88, 165.26, 157.23, 149.52 (d, J = 1.7 Hz), 148.01, 136.22, 133.35, 132.78, 131.46, 128.34, 123.72, 121.20, 110.08, 41.29, 31.14, 31.04, 30.04, 29.76 (q, J = 77.9 Hz), 20.62, 17.91; HRMS (APCI, m/z): calcd. for $C_{22}H_{23}F_3N_3O_2S^+$ ($[M+H]^+$) 450.1463, found 450.1458.

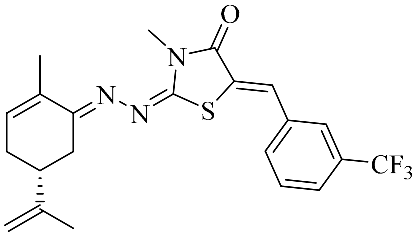

(E)-3-methyl-2-(((R,E)-2-methyl-5-(prop-1-en-2-yl)cyclohex-2-en-1-ylidene)hydrazineylidene)-5-((Z)-3-(trifluoromethyl)benzylidene)thiazolidin-4-one (compound **4t**): yellow solid; yield, 81.5%; m.p.: 121.0 – 122.4 °C; IR (KBr, ν/cm^{-1}): 3018.37 (=C-H, Ar-H), 2924.49 (C-H), 1710.20 (C=O), 1602.04 (C=C), 1563.27, 1432.65, 1371.43 (Ar), 695.92 (C-S-C); ^{1}H NMR (600 MHz, $CDCl_3$) δ 7.81 (s, 1H, =C-H), 7.77 (d, J = 7.3 Hz, 1H, Ar-H), 7.70 (s, 1H, Ar-H), 7.65 – 7.59 (m, 2H, Ar-H), 6.29 (dd, J = 3.4, 1.4 Hz, 1H, =C-H), 4.82 (d, J = 8.7 Hz, 2H, =CH_2), 3.48 – 3.43 (m, 4H, N-CH_3 & CH), 2.50 – 2.34 (m, 2H, CH_2), 2.26 – 2.18 (m, 2H, CH_2), 2.00 (s, 3H, =C-CH_3), 1.80 (s, 3H, =C-CH_3); ^{13}C NMR (151 MHz, $CDCl_3$): δ 13C NMR (151 MHz, CDCl3) δ 166.70, 165.35, 157.10, 147.98, 136.33, 134.99, 133.36, 132.40, 131.51 (q, J = 32.6 Hz), 129.54, 128.12, 126.82 (q, J = 3.9 Hz), 125.77 (q, J = 3.7 Hz), 125.00, 123.73 (d, J = 272.4 Hz), 110.09, 41.27, 31.14, 31.04, 30.06, 20.61, 17.79; HRMS (APCI, m/z): calcd. for $C_{22}H_{23}F_3N_3OS^+$ ($[M+H]^+$) 434.1514, found 434.1508.

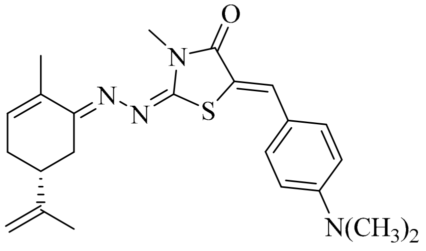

(*E*)-5-((*Z*)-4-(dimethylamino)benzylidene)-3-methyl-2-(((*R*,*E*)-2-methyl-5-(prop-1-en-2-yl) cyclohex-2-en-1-ylidene)hydrazineylidene)thiazolidin-4-one (compound **4u**): yellow solid; yield, 84.2%; m.p.: 195.0 – 195.8 °C; IR (KBr, ν/cm^{-1}): 3065.44 (=C-H, Ar-H), 2916.46 (C-H), 1704.23 (C=O), 1602.19 (C=C), 1579.74, 1522.60, 1426.68, 1369.54 (Ar), 689.95 (C-S-C); ^{1}H NMR (600 MHz, $CDCl_3$) δ 7.66 (s, 1H, =C-H), 7.55 – 7.48 (m, 2H, Ar-H), 6.80 – 6.73 (m, 2H, Ar-H), 6.28 – 6.23 (m, 1H, =C-H), 4.82 (dd, *J* = 3.3, 1.7 Hz, 2H, =CH_2), 3.50 – 3.43 (m, 4H, N-CH_3 & CH), 3.06 (s, 6H, -N(CH_3)$_2$), 2.50 – 2.33 (m, 2H, CH_2), 2.25 – 2.18 (m, 2H, CH_2), 2.03 (s, 3H, =C-CH_3), 1.80 (s, 3H, =C-CH_3); ^{13}C NMR (151 MHz, $CDCl_3$) δ 167.74, 164.21, 158.60, 150.96, 148.19, 135.37, 133.53, 132.11, 131.25, 121.83, 116.23, 111.94, 109.97, 41.33, 40.09, 31.04, 29.78, 20.65, 17.97; HRMS (APCI, m/z): calcd. for $C_{23}H_{29}N_4OS^+$ ([M+H]$^+$) 409.2062, found 409.2057.

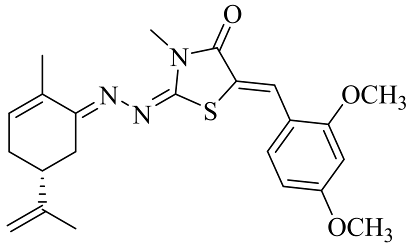

(*E*)-5-((*Z*)-2,4-dimethoxybenzylidene)-3-methyl-2-(((*R*,*E*)-2-methyl-5-(prop-1-en-2-yl)cyclohex-2-en-1-ylidene)hydrazineylidene)thiazolidin-4-one (compound **4v**): yellow solid; yield, 87.8%; m.p.: 246.1 – 247.3 °C; IR (KBr, ν/cm^{-1}): 3046.94 (=C-H, Ar-H), 2957.14 (C-H), 1695.92 (C=O), 1602.04 (C=C), 1587.76, 1557.14, 1504.08, 1436.73, 1371.43 (Ar), 695.92 (C-S-C); ^{1}H NMR (600 MHz, $CDCl_3$) δ 8.07 (s, 1H, =C-H), 7.57 (d, *J* = 8.6 Hz, 1H, Ar-H), 6.63 (dd, *J* = 8.6, 2.3 Hz, 1H, Ar-H), 6.49 (d, *J* = 2.3 Hz, 1H, Ar-H), 6.25 (dd, *J* = 3.5, 1.4 Hz, 1H, =C-H), 4.82 (d, *J* = 5.0 Hz, 2H, =CH_2), 3.88 (d, *J* = 7.8 Hz, 6H, -OCH_3), 3.57 – 3.35 (m, 4H, N-CH_3 & CH), 2.51 – 2.32 (m, 2H, CH_2), 2.26 – 2.15 (m, 2H, CH_2), 2.01 (s, 3H, =C-CH_3), 1.80 (s, 3H, =C-CH_3); ^{13}C NMR (151 MHz, $CDCl_3$): δ 167.46, 164.41, 162.43, 159.86, 158.42, 148.15, 135.52, 133.48, 130.43, 125.36, 119.53, 116.50, 109.98, 105.06, 98.49, 55.55, 55.51, 41.31, 31.03, 29.79, 20.63, 17.92; HRMS (APCI, m/z): calcd. for $C_{23}H_{28}N_3O_3S^+$ ([M+H]$^+$) 426.1851, found 426.1846.

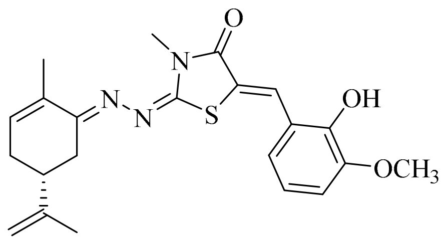

(*E*)-5-((*Z*)-2-hydroxy-3-methoxybenzylidene)-3-methyl-2-(((*R*,*E*)-2-methyl-5-(prop-1-en-2-yl) cyclohex-2-en-1-ylidene)hydrazineylidene)thiazolidin-4-one (compound **4w**): yellow solid; yield, 82.5%; m.p.: 250.4 – 252.9 °C; IR (KBr, ν/cm^{-1}): 3410.33 (O-H), 3030.74 (=C-H, Ar-H), 2965.44, 2912.38 (C-H), 1669.53 (C=O), 1600.15 (C=C), 1569.53, 1479.74, 1422.60, 1373.62 (Ar), 685.87 (C-S-C); ^{1}H NMR (600 MHz, DMSO-d_6) δ 8.25 (s, 1H, =C-H), 6.81 (dd, *J* = 8.1, 1.4 Hz, 1H, Ar-H), 6.46 (dd, *J* = 7.5, 1.4 Hz, 1H, Ar-H), 6.24 (dd, *J* = 3.1, 1.5 Hz, 1H, =C-H), 5.96 (t, *J* = 7.7 Hz, 1H, Ar-H), 4.78 (d, *J* = 8.0 Hz, 2H, =CH_2), 3.61 (s, 3H, -OCH_3), 3.36 – 3.26 (m, 4H, N-CH_3 & CH), 2.41 – 2.27 (m, 2H, CH_2), 2.23 – 2.14 (m, 2H, CH_2), 1.91 (s, 3H, =C-CH_3), 1.74 (s, 3H, =C-CH_3); ^{13}C NMR (151 MHz, DMSO-d_6): δ 167.70, 166.91, 162.27, 160.25, 153.37, 148.33, 135.18, 133.03, 132.54, 122.01, 120.40, 111.79, 110.54, 107.97, 107.39, 55.31, 41.18, 30.81, 30.75, 29.68, 20.88, 18.17; HRMS (APCI, m/z): calcd. for $C_{22}H_{26}N_3O_3S^+$ ([M+H]$^+$) 412.1695, found 412.1689.

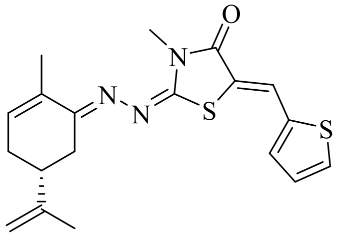

(2*E*,5*Z*)-3-methyl-2-(((*R*,*E*)-2-methyl-5-(prop-1-en-2-yl)cyclohex-2-en-1-ylidene)hydrazineylidene) -5-(thiophen-2-ylmethylene)thiazolidin-4-one (compound **4x**): yellow solid; yield, 84.3%; m.p.: 165.1 – 165.7 °C; IR (KBr, ν/cm^{-1}): 3120.41, 3069.39 (=C-H, Ar-H), 2944.90, 2914.29 (C-H), 1697.96 (C=O), 1620.41, 1597.96 (C=C), 1432.65, 1375.51 (Ar), 714.29 (C-S-C); ^{1}H NMR (600 MHz, $CDCl_3$) δ 7.89 (s, 1H, =C-H), 7.59 (d, *J* = 5.0 Hz, 1H, Ar-H), 7.38 (d, *J* = 3.6 Hz, 1H, Ar-H), 7.18 (dd, *J* = 5.0, 3.7 Hz, 1H, Ar-H), 6.29 – 6.25 (m, 1H, =C-H), 4.82 (dd, *J* = 6.0, 0.9 Hz, 2H, =CH_2), 3.47 – 3.43 (m, 4H, N-CH_3 & CH), 2.50 – 2.34 (m, 2H, CH_2), 2.26 – 2.18 (m, 2H, CH_2), 2.02 (s, 3H, =C-CH_3), 1.80 (s, 3H, =C-CH_3); ^{13}C NMR (151 MHz, $CDCl_3$): δ 166.89, 164.86, 157.65, 148.06, 138.63, 135.94, 133.45, 131.75, 130.32, 128.27, 122.88, 121.01, 110.05, 41.30, 31.14, 31.05, 30.01, 20.63, 17.88; HRMS (APCI, m/z): calcd. for $C_{19}H_{22}N_3OS_2^+$ ([M+H]$^+$) 372.1204, found 372.1199.

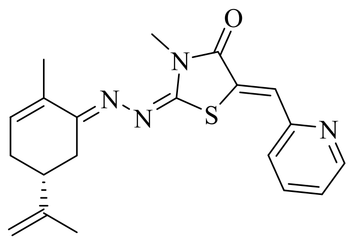

(2*E*,5*Z*)-3-methyl-2-(((*R*,*E*)-2-methyl-5-(prop-1-en-2-yl)cyclohex-2-en-1-ylidene)hydrazineylidene) -5-(pyridin-2-ylmethylene)thiazolidin-4-one (compound **4y**): yellow solid; yield, 86.0%; m.p.: 145.8 – 146.7 ºC; IR (KBr, ν/cm^{-1}): 3081.63, 3030.61 (=C-H, Ar-H), 2963.27, 2942.86, 2914.29 (C-H), 1708.16 (C=O), 1626.53, 1608.16 (C=C), 1432.65, 1377.55 (Ar), 706.12 (C-S-C); ^{1}H NMR (600 MHz, $CDCl_3$) δ 8.83 (d, *J* = 2.2 Hz, 1H, Ar-H), 8.59 (dd, *J* = 4.8, 1.5 Hz, 1H, Ar-H), 7.90 – 7.86 (m, 1H, Ar-H), 7.65 (s, 1H, =C-H), 7.42 (dd, *J* = 8.0, 4.8 Hz, 1H, Ar-H), 6.29 – 6.26 (m, 1H, =C-H), 4.82 – 4.79 (m, 2H, =CH_2), 3.46 – 3.41 (m, 4H, N-CH_3 & CH), 2.49 – 2.33 (m, 2H, CH_2), 2.25 – 2.17 (m, 2H, CH_2), 1.99 (s, 3H, =C-CH_3), 1.79 (s, 3H, =C-CH_3); ^{13}C NMR (151 MHz, $CDCl_3$): δ 166.58, 165.39, 156.98, 151.31, 149.75, 147.96, 136.36, 136.07, 133.33, 130.27, 126.08, 125.36, 123.74, 110.10, 41.26, 31.14, 31.03, 30.11, 20.61, 17.89; HRMS (APCI, m/z): calcd. for $C_{20}H_{23}N_4OS^+$ ([M+H]$^+$) 367.1593, found 367.1587.

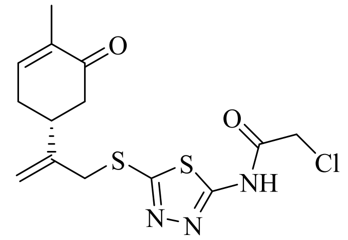

(*R*)-2-chloro-*N*-(5-((2-(4-methyl-5-oxocyclohex-3-en-1-yl)allyl)thio)-1,3,4-thiadiazol-2-yl) acetamide (intermediate **6** / *L*-carvone-derived 1,3,4-thiadiazole-amide): white solid; yield, 71.6%; m.p.: 102.5 – 103.0 ºC; ^{1}H NMR (600 MHz, $CDCl_3$) δ 6.77 (ddd, *J* = 5.6, 2.4, 1.3 Hz, 1H, =C-H), 5.22 (s, 1H, =CH_2-a), 5.06 (s, 1H, =CH_2-b), 4.46 (s, 2H, Cl-CH_2), 3.96 (s, 2H, -S-CH_2), 3.01 – 2.96 (m, 1H, CH), 2.70 – 2.55 (m, 2H, CH_2), 2.44 – 2.33 (m, 2H, CH_2), 1.80 (s, 3H, -CH_3); ^{13}C NMR (151 MHz, $CDCl_3$) δ 198.88, 164.98, 160.28, 160.14, 144.75, 144.04, 135.68, 115.36, 43.22, 42.11, 39.03, 38.84, 31.55, 15.69.
